# cNEK6 induces gemcitabine resistance by promoting glycolysis in pancreatic ductal adenocarcinoma via the SNRPA/PPA2c/mTORC1 axis

**DOI:** 10.1038/s41419-024-07138-y

**Published:** 2024-10-11

**Authors:** Ge Li, Fei-Fei She, Cheng-Yu Liao, Zu-Wei Wang, Yi-Ting Wang, Yong-Din Wu, Xiao-Xiao Huang, Cheng-Ke Xie, Hong-Yi Lin, Shun-Cang Zhu, Yin-Hao Chen, Zhen-heng Wu, Jiang-Zhi Chen, Shi Chen, Yan-Ling Chen

**Affiliations:** 1 Department of Hepatobiliary Surgery and Fujian Institute of Hepatobiliary Surgery, Fujian Medical University Union Hospital, Fuzhou, China; 2 Fujian Medical University Cancer Center, Fuzhou, China; 3 Fujian Key Laboratory of Tumor Microbiology, Department of Medical Microbiology, Fujian Medical University, Fuzhou, China; 4 Key Laboratory of Ministry of Education for Gastrointestinal Cancer, Fujian Medical University, Fuzhou, China; 5 Fuzhou University Affiliated Provincial Hospital, Fuzhou, China; 6 Department of Hepatopancreatobiliary Surgery, Fujian Provincial Hospital, Fuzhou, China; 7 Shengli Clinical Medical College of Fujian Medical University, Fuzhou, China; 8 Fujian Institute of Hematology, Fujian Provincial Key Laboratory on Hematology, Fujian Medical University Union Hospital, Fuzhou, China

**Keywords:** Oncogenes, Ubiquitylation

## Abstract

Resistance to gemcitabine in pancreatic ductal adenocarcinoma (PDAC) leads to ineffective chemotherapy and, consequently, delayed treatment, thereby contributing to poor prognosis. Glycolysis is an important intrinsic reason for gemcitabine resistance as it competitively inhibits gemcitabine activity by promoting deoxycytidine triphosphate accumulation in PDAC. However, biomarkers are lacking to determine which patients can benefit significantly from glycolysis inhibition under the treatment of gemcitabine activity, and a comprehensive understanding of the molecular mechanisms that promote glycolysis in PDAC will contribute to the development of a strategy to sensitize gemcitabine chemotherapy. In this study, we aimed to identify a biomarker that can robustly indicate the intrinsic resistance of PDAC to gemcitabine and guide chemotherapy sensitization strategies. After establishing gemcitabine-resistant cell lines in our laboratory and collecting pancreatic cancer and adjacent normal tissues from gemcitabine-treated patients, we observed that circRNA hsa_circ_0008383 (namely cNEK6) was highly expressed in the peripheral blood and tumor tissues of patients and xenografts with gemcitabine-resistant PDAC. cNEK6 enhanced resistance to gemcitabine by promoting glycolysis in PDAC. Specifically, cNEK6 prevented K48 ubiquitination of small ribonucleoprotein peptide A from the BTRC, a ubiquitin E3 ligase; thus, the accumulated SNRPA stopped PP2Ac translation by binding to its G-quadruplexes in 5′ UTR of mRNA. mTORC1 pathway was aberrantly phosphorylated and activated owing to the absence of PP2Ac. The expression level of cNEK6 in the peripheral blood and tumor tissues correlated significantly and positively with the activation of the mTORC1 pathway and degree of glycolysis. Hence, the therapeutic effect of gemcitabine is limited in patients with high cNEK6 levels, and in combination with the mTORC1 inhibitor, rapamycin, can enhance sensitivity to gemcitabine chemotherapy.

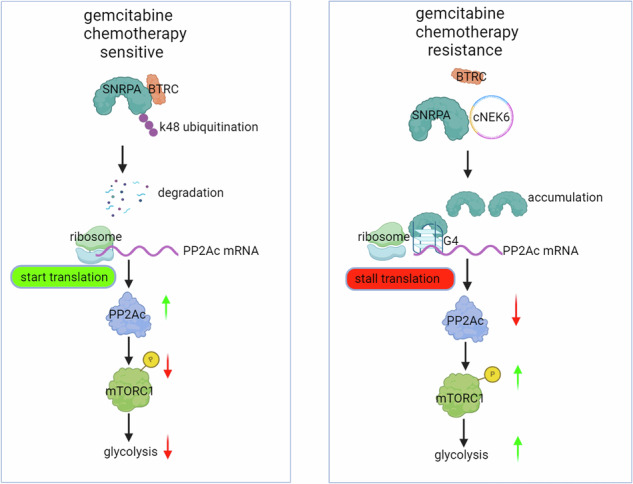

## Introduction

Pancreatic cancer is a highly malignant tumor, and a significant proportion of patients with pancreatic ductal adenocarcinoma (PDAC) lose the opportunity to undergo radical surgery when diagnosed [1]. Even patients with PDAC resection experience local recurrence or distant metastasis after surgery [2]. Therefore, an adjuvant treatment for PDAC is very necessary [3]. However, many patients are resistant to gemcitabine, the most commonly used first-line and cornerstone drug in the combination chemotherapy for PDAC [4, 5], thereby causing insensitivity to chemotherapy and delay in treatment. Presently, clear markers that can accurately indicate whether pancreatic cancer is resistant to gemcitabine are lacking; thus, identifying reliable biomarkers that can precisely indicate the sensitivity of PDAC to gemcitabine and provide an appropriate chemotherapy sensitization strategy is required.

There is growing evidence that tumor metabolism, including glucose, lipid, and amino acid metabolism, can greatly affect the efficacy of PDAC chemotherapy [6]. Tumors largely rely on the energy and metabolites produced by glycolysis [7]. Oxidative stress caused by reactive oxygen species produced by gemcitabine inactivates glycolysis key enzymes, such as pyruvate kinase M2 and glyceraldehyde 3-phosphate dehydrogenase (GAPDH), effectively killing tumors [8, 9]. By enhancing glycolysis, pancreatic cancer cells utilize glycolytic metabolites to resist gemcitabine [7]. Therefore, a full understanding of the molecular mechanisms of PDAC-promoting glycolysis will contribute to the development of a potential strategy for gemcitabine chemotherapy sensitization.

CircRNAs have molecular stability and specificity; thus, they are not easily degraded and can gradually accumulate and be widely distributed in the body, making them valuable biomarkers. Their liquid biopsy in body fluids (including blood, saliva, and urine) is convenient, less invasive, and more feasible [10]. In addition, circRNAs have various functions, including sponge adsorption of microRNAs, interaction with RNA-binding proteins, and encoding of peptides, hence playing important roles in tumor development, metastasis, and drug resistance [11]. Regarding glycolysis regulation, circRNAs promote glycolysis through the PI3K/Akt and Wnt/ β-catenin pathways in ovarian [12] and papillary thyroid cancers [13], respectively. In pancreatic cancer, circRNAs resist chemotherapy by promoting glycolysis [14–16]. However, the above studies are limited to the function of circRNAs as microRNA sponges, and whether circRNAs can regulate glycolysis in ways besides acting as miRNA sponges or serve as biomarkers for indicating the degree of glycolysis is largely unknown. Hence, the circRNAs mechanism of glycolysis regulation requires further investigation.

Small ribonucleoprotein peptide A (SNRPA) is an RNA-binding protein that recognizes splicing sites and facilitates the subsequent assembly of spliceosomes [17]. In addition to serving as a splicing-related regulatory factor, SNRPA can combined with G-quadruplexes (G4) [18], a high-level structure formed by the folding of DNA or RNA sequences that are rich in repetitive guanine (G), and play a crucial role in tumorigenesis and progression [19]. Proteins that bind to the G4 structures upstream of the translation start site can prevent the binding of ribosomes to the target genes, thus blocking translation [20]. SNRPA is abnormally elevated in many cancers and promotes the occurrence, development, and metastasis of tumors [21–23]. Whether SNRPA or the G4 participates in boosting glycolysis and gemcitabine resistance in PDAC is yet to be studied.

This study demonstrates for the first time that cNEK6, a circRNA, can induce gemcitabine resistance through glycolysis. Specifically, cNEK6 prevents the ubiquitination-mediated degradation of SNRPA. The accumulated SNRPA inhibits the translation of PP2Ac, a dephosphorylase of mTORC1, by combining the G4 structures in the 5′ UTR of PP2Ac mRNA, consequently causing abnormal activation of the mTORC1 pathway to promote glycolysis (Figure Abstract). Our research shows that cNEK6 can be a biomarker, robustly indicating gemcitabine intrinsic resistance and suggesting the use of mTORC1 inhibitors to improve chemotherapy sensitization of PDAC to gemcitabine.

## Results

### cNEK6 promotes gemcitabine chemotherapy resistance in PDAC

**Fig. 1:**
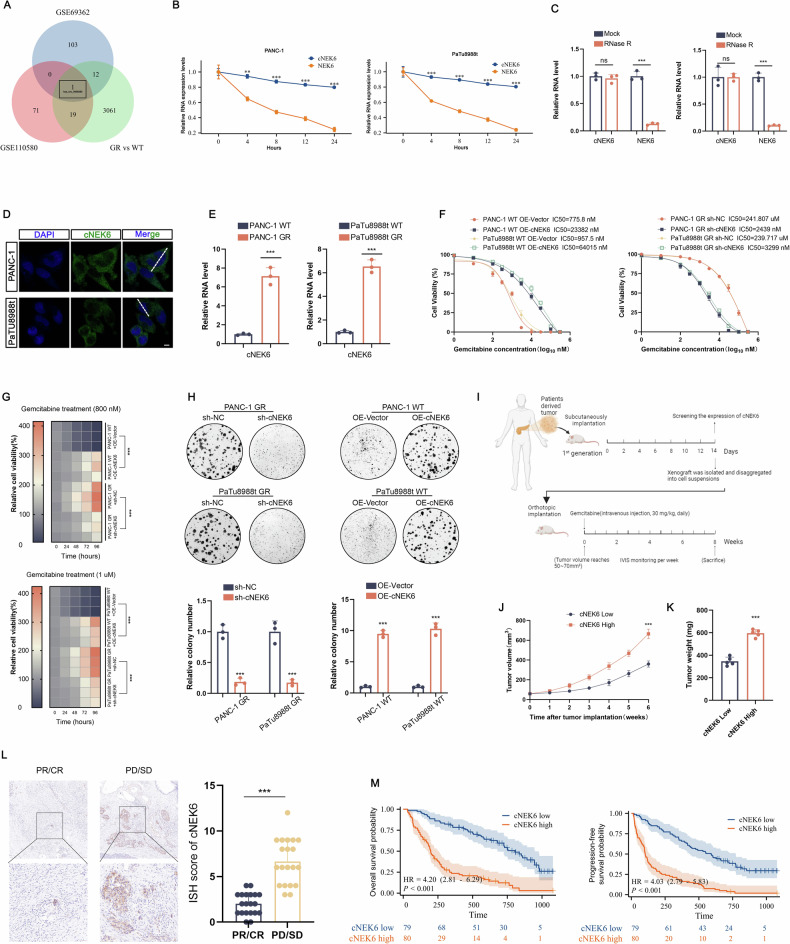
cNEK6 promotes gemcitabine chemotherapy resistance in PDAC. **A** Venn diagram showing the intersection of differentially expressed circRNAs among the three gene sets. **B** Relative RNA levels of cNEK6 and NEK6 after actinomycin D treatment at different time points (*n* = 3) (RT-qPCR). **C** RT-qPCR was used to analyze the expression of cNEK6 and NEK6 after RNase R treatment of PANC-1 and PaTu8988t cells (*n* = 3). **D** Subcellular localization of cNEK6 detected by fluorescence in situ hybridization (FISH). Scale bars = 5 μm. **E**. Expression levels of cNEK6 between gemcitabine-resistant groups (PANC-1 GR and PaTu8988t GR) and their control groups (PANC-1 WT and PaTu8988t WT) (*n* = 3). **F**–**H** Effect of cNEK6 on the proliferative ability (IC50 (**F**), cell viability (**G**), and colony formation (**H**)) of gemcitabine-resistant groups (PANC-1 GR and PaTu8988t GR) and their control groups (PANC-1 WT and PaTu8988t WT) treated with gemcitabine (*n* = 3). **I**–**K** Schematic representation of the treatment regimen for patient-derived tumor xenograft (PDX) models (**I**) (*n* = 5). Xenografts were isolated and measured after euthanasia (**J**, **K**). **L**. CircRNA in situ hybridization **(**ISH) for cNEK6 in PDAC tissues. Scale bars = 100 μm (*n* = 20). **M** Kaplan–Meier analysis of overall survival and progression-free survival of patients with PDAC based on the expression of cNEK6.

To identify the circRNAs related to gemcitabine chemotherapy resistance in PDAC, we constructed gemcitabine-resistant PDAC cell lines, PANC-1 GR, and analyzed the circRNAs differentially expressed between the PANC-1 GR group and the wild-type (WT) PANC-1 group using circRNA sequencing. Simultaneously, we analyzed two GEO datasets: GSE110580 (differential expression of circRNA in three pairs of PANC-1 GR group and wild-type control group) and GSE69362 (differential expression of circRNA in six pairs of PDAC and its adjacent tissues). The volcano map shows differentially expressed circRNAs (|fold change| > 1 and *P* < 0.05) in the three datasets (Suppl. Fig. 1A). The differentially expressed circRNAs were intersected by Venny, and a circRNA, hsa_circ_0008383, which was significantly overexpressed in the gemcitabine-resistant group in all three datasets, was screened out (Fig. 1A). hsa_circ_0008383 is generated by the reverse splicing of exons 2–7 of the human NEK6 gene, which contains 651 nucleotides (Suppl. Fig. 1B). Therefore, we named hsa_circ_0008383 as circ-NEK6 (cNEK6).

To confirm the circular characteristics of cNEK6, we designed divergent and convergent primers and amplified cNEK6 using divergent primers in complementary DNA (cDNA) from PDAC cells (Suppl. Fig. 1C). Compared with the parental linear mRNA NEK6, cNEK6 was more stable when treated with the transcription inhibitor actinomycin D (Fig. 1B) and showed significant resistance to RNase R treatment (Fig. 1C, Suppl. Fig. 1D). Immunofluorescence experiments showed that cNEK6 was localized in the cytoplasm (Fig. 1D, Suppl. Fig. 1E).

Quantitative reverse transcription polymerase chain reaction (RT-qPCR) confirmed that cNEK6 was highly expressed in gemcitabine-resistant PDAC cell lines (PANC-1 GR and PaTu8988t GR) compared to the wild-type control groups (Fig. 1E). After cNEK6 was knocked down in the PANC-1 GR and PaTu8988t GR cell lines (Suppl. Fig. 1F), the gemcitabine-resistant groups were more sensitive to gemcitabine, as the IC50 value (Fig. 1F) and cell activity (Fig. 1G) decreased, and the ability to form clones was weakened by gemcitabine treatment (Fig. 1H). In contrast, cNEK6 overexpression in the control group led to significant gemcitabine resistance (Fig. 1F–H). After collecting tumor samples from patients with PDAC, RT-qPCR was performed to detect the cNEK6 expression, and xenograft models were constructed in nude mice. Gemcitabine was administered after induction of tumorigenesis (Fig. 1I). Tumors with high cNEK6 expression were resistant to gemcitabine as they grew faster, and the nude mice had poor outcomes (Fig. 1J, K). In addition, we collected tumor biopsy samples from patients with PDAC before chemotherapy and divided the tumor samples into partial/complete response (PR/CR) and progressive/stable disease (PD/SD) groups according to the chemotherapy outcomes from their original patients who received gemcitabine single-drug chemotherapy. CircRNA in situ hybridization (ISH) analysis showed that cNEK6 was highly expressed in the PD/SD group (Fig. 1L), with a worse overall and disease-free survival (Fig. 1M). The above in vivo, and in vitro experiments and the clinical data showed cNEK6 was positively associated with gemcitabine chemotherapy resistance in PDAC.

### cNEK6 enhances glycolysis in PDAC to resist gemcitabine

**Fig. 2:**
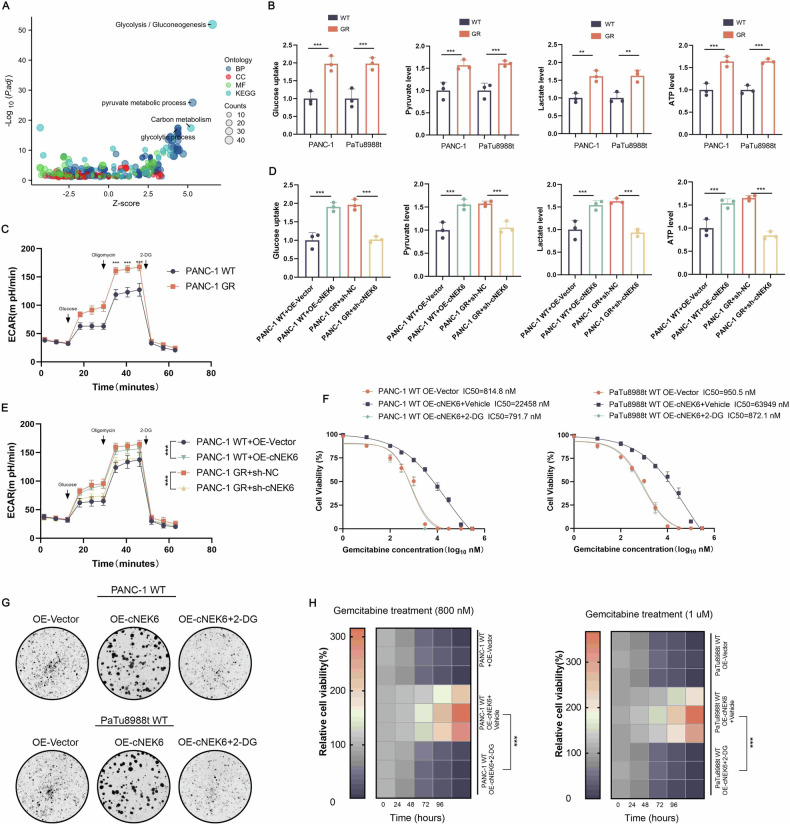
cNEK6 enhances glycolysis in PDAC to resist gemcitabine. **A** Bubble plot showing GO and KEGG analyses of differentially expressed genes between the cNEK6 over-expression and control PANC-1 cells. **B** Glucose uptake, pyruvate levels, lactate production, and ATP levels were compared between gemcitabine-resistant groups (PANC-1 GR and PaTu8988t GR) and their control groups (PANC-1 WT and PaTu8988t WT) (*n* = 3). **C** ECAR was determined between the PANC-1 GR and PANC-1 WT cell lines (*n* = 3). **D** Effect of cNEK6 on glucose uptake, pyruvate levels, lactate production, and ATP levels in PANC-1 GR cells and their control, PANC-1 WT cells (*n* = 3). **E** ECAR showing the effect of cNEK6 on glycolysis in PANC-1 GR cells and control PANC-1 WT cells (*n* = 3). **F**–**H** Effect of the glycolysis inhibitor, 2-DG, on cNEK6 induced changes on proliferative ability (IC50 (**F**), colony formation (**G**), and cell viability (**H**)) of PANC-1 WT and PaTu8988t WT cell lines after gemcitabine treatment (*n* = 3).

We sequenced the differentially expressed genes between the OE-cNEK6 PANC-1 and control groups to explore how cNEK6 promotes gemcitabine resistance in PDAC (Suppl. Fig. 2A) and performed Gene Ontology analysis. The glycolytic pathway was significantly activated in the OE-cNEK6 PANC-1 group (Fig. 1A). Gene Sequence enrichment analysis also showed that glycolysis was activated in the OE-cNEK6 PANC-1 group (Suppl. Fig. 2B). As glycolysis has been reported to play an important role in PDAC gemcitabine resistance [7], we hypothesized that cNEK6 contributes to gemcitabine resistance by regulating glycolysis. Therefore, we tested the glycolysis level in gemcitabine-resistant PDAC cell lines and control groups. The results showed that glucose uptake the levels of pyruvate, lactate, and ATP, and glycolysis levels detected by extracellular acidification rate (ECAR), were significantly enhanced in the gemcitabine-resistant groups (Fig. 2B, C). We confirmed the effect of cNEK6 on glycolysis regulation, we found that cNEK6 overexpression promoted glucose uptake and pyruvate, lactate, ATP, and glycolysis levels in PANC-1 and PaTu8988t cell lines (Fig. 2D, E). In contrast, the opposite was observed in PANC-1 GR and PaTu8988t GR cell lines when cNEK6 was knocked down (Fig. 2D, E). These results indicated that glycolysis enhanced gemcitabine-resistant PDAC and cNEK6-promoted glycolysis. To investigate whether cNEK6 facilitated gemcitabine resistance through glycolysis, we added the glycolysis inhibitor, 2-DG, to PANC-1 and PaTu8988t cell lines that overexpressed cNEK6 and treated them with gemcitabine. The results showed that after the 2-DG application, the IC50 values, cloning ability, and cell activity were significantly attenuated in the cENK6 overexpression group (Fig. 2F, H, Suppl. Fig. 2C). The above experiments demonstrated that cNEK6 could lead to gemcitabine resistance by promoting glycolysis in PDAC.

### cNEK6 promotes glycolysis in PDAC through SNRPA

**Fig. 3:**
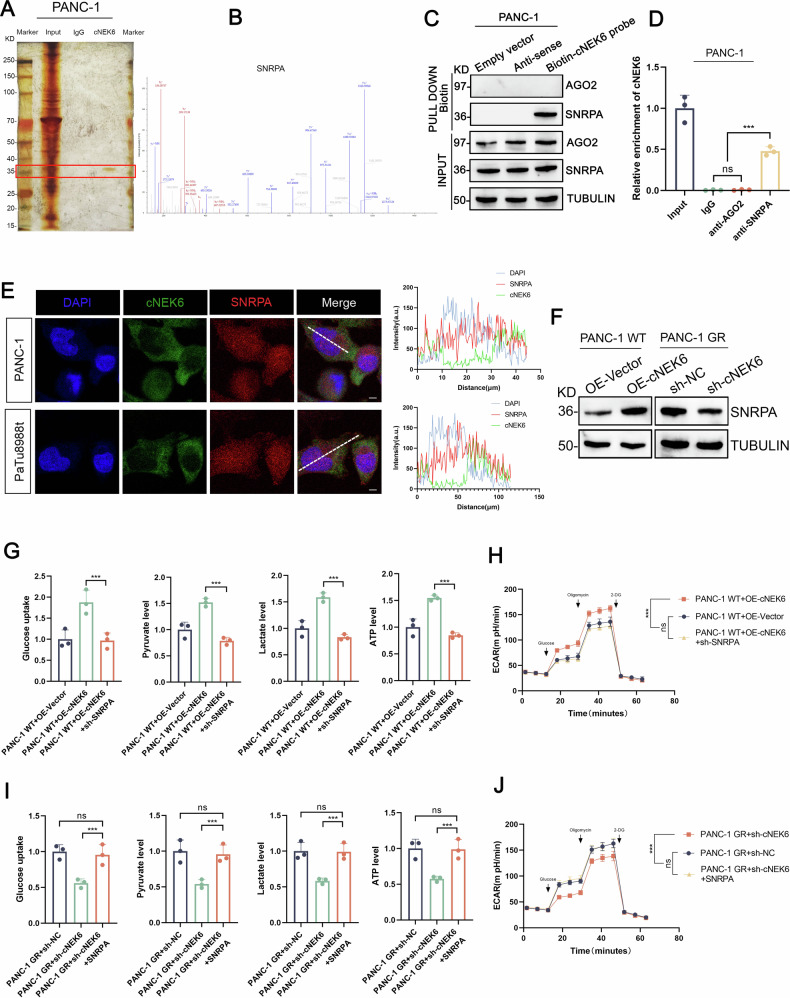
cNEK6 promotes glycolysis in PDAC through SNRPA. **A**, **B** Cell lysate from PNC-1 cells was incubated with the cNEK6 probe. Potential cNEK6-binding proteins were pulled down, followed by RAP assay, visualized by silver staining (**A**), and identified by mass spectrometry (**B**). **C** Binding of cNEK6 to AGO2 or SNRPA was determined using an RNA pull-down assay. **D** Binding of AGO2 or SNRPA to cNEK6 was determined using the RIP assay (*n* = 3). **E** Subcellular localization of cNEK6 and SNRPA detected by FISH and immunofluorescence, respectively. **F** Protein levels of SNRPA in PANC-1 GR cell lines and their control, PANC-1 WT cells, transfected with sh-cNEK6 and OE-cNEK6, respectively. **G**, **H** Effect of SNRPA knockdown on cNEK6 induced changes in glucose uptake, pyruvate levels, lactate production, ATP levels (**G**), and ECAR (**H**) in PANC-1 WT cell lines (*n* = 3). **I**, **J**. Effect of SNRPA supplementation on sh-cNEK6 induced changes in glucose uptake, pyruvate levels, lactate production, ATP levels (**I**), and ECAR (**J**) in PANC-1 GR cell lines (*n* = 3).

CircRNA can act as a microRNA sponge, interact with RBP, or encode peptides; therefore, we explored how cNEK6 could promote glycolysis. First, we found that cNEK6 did not have the open reading frame region (Suppl. Fig. 3A), which is necessary for encoding amino acids. Next, we performed RNA immunoprecipitation (RIP) using biotin-labeled cENK6 and control antibodies, followed by silver staining and mass spectroscopy to identify potential proteins that could interact with cNEK6. According to the highest binding potential protein of cNEK6 predicted in the RBPDB database (rbpdb.ccbr.utoronto.ca/) (Suppl. Fig. 3B), we found a clear band at 36 kd by silver staining (Fig. 3A), and mass spectroscopy analysis identified the protein to be SNRPA (Fig. 3B). We further performed RNA pull-down analysis and confirmed that SNRPA could interact with cNEK6 and not with AGO2 (Fig. 3C), which is crucial for circRNA to act as a microRNA sponge. Using SNRPA and AGO2 antibodies, RIP analysis confirmed that SNRPA, rather than AGO2, binds to cENK6 (Fig. 3D). Immunofluorescence showed the co-localization of SNRPA and cNEK6 in the cytoplasm (Fig. 3E).

We investigated whether SNRPA participated in cNEK6-regulated glycolysis. Western blot and RT-qPCR analysis showed that cNEK6 promoted SNRPA expression at the protein level while knocking down cNEK6 decreased SNRPA protein level (Fig. 3F); however, cNEK6 could not affect the mRNA level of SNRPA (Suppl. Fig. 3C). SNRPA knockdown in PANC-1-OE-cNEK6 cells attenuated the cNEK6-enhanced glycolysis level (Fig. 3G, H). In contrast, supplementation with SNRPA in PANC-1-GR-sh-cNEK6 cells could enhance glycolysis inhibited by cENK6 knockdown (Fig. 3I, J). The above results indicated that cNEK6 could enhance glycolysis by increasing the expression of SNRPA.

### cNEK6 stabilizes SNRPA by suppressing the K48 ubiquitination

**Fig. 4:**
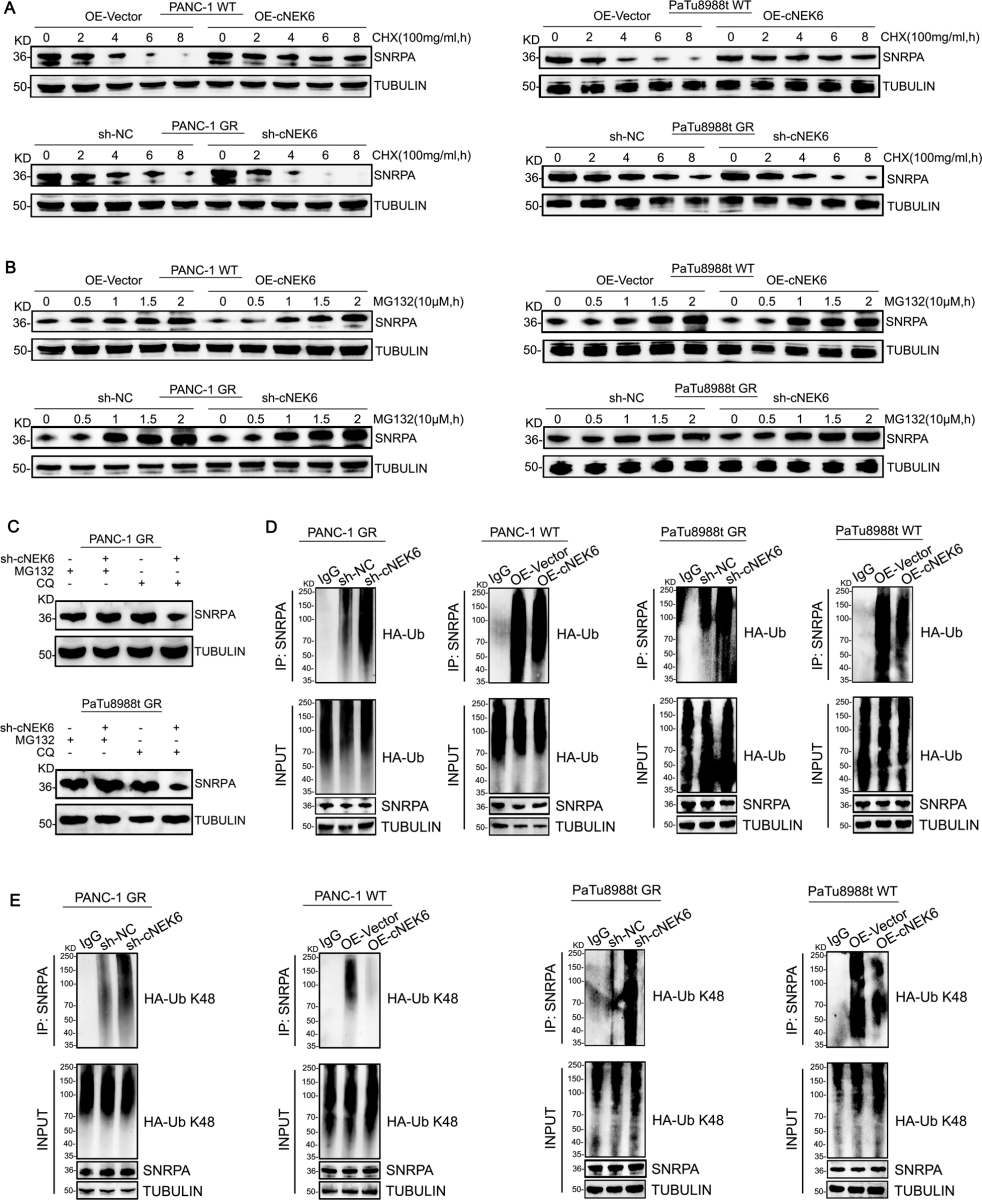
cNEK6 stabilizes SNRPA by suppressing the K48 ubiquitination. **A**, **B** After cycloheximide (CHX, 100 μg/mL) (A) or MG132 treatment (10 μM) (**B**), temporal changes in SNRPA protein level in gemcitabine-resistance (PANC-1 GR and PaTu8988t GR) and control groups (PANC-1 WT and PaTu8988t WT) transfected with sh-cNEK6 and OE-cNEK6, respectively. **C** Effect of Chloroquine (CQ, 25 μM) or MG132 (10 μM) on cNEK6 induced changes of SNRPA protein level. **D, E**. Ubiquitination of SNRPA in gemcitabine-resistant (PANC-1 GR and PaTu8988t GR) and control groups (PANC-1 WT and PaTu8988t WT) transfected with sh-cNEK6 and OE-cNEK6, respectively. Western blot analysis was performed using antibodies against Ub (**D**), and K48-Ub (**E**).

To investigate how cNEK6 increases the protein levels of SNRPA, we applied cycloheximide (CHX) or MG132 to the PANC-1/PaTu8988t-OE-cNEK6 and PANC-1/PaTu8988t-GR-sh-cNEK6 groups and their control groups to inhibit protein synthesis or degradation. The results showed that after applying CHX, cNEK6 increased the protein stability of SNRPA, resulting in a slower degradation of SNRPA, whereas cNEK6 knockdown caused the opposite effect (Fig. 4A, Suppl. Fig. 4A). However, after using MG132 to block protein degradation, no difference was observed in the SNRPA levels of the above groups (Fig. 4B, Suppl. Fig. 4B), indicating that cNEK6 did not affect SNRPA synthesis. Further, we applied MG132 or chloroquine to PANC-1/PaTu8988t-GR-sh-cNEK6 cells and their control groups to block proteasome ubiquitination or protein autophagy degradation. The results showed that inhibiting ubiquitination degradation instead of autophagy could inhibit the accelerated degradation of SNRPA caused by cNEK6 knockdown (Fig. 4C). This indicated that cNEK6 might suppress SNRPA ubiquitination and degradation. To verify this hypothesis, we tested the effect of cNEK6 on SNRPA ubiquitination. The co-immunoprecipitation (CO-IP) results showed that cNEK6 inhibited SNRPA ubiquitination, whereas cNEK6 knockdown enhanced SNRPA ubiquitination (Fig. 4D). cNEK6 mainly regulated K48 ubiquitination (Fig. 4E) rather than K63 ubiquitination of SNRPA (Suppl. Fig. 4C). These results indicated that cNEK6 stabilized SNRPA by suppressing K48 ubiquitination.

### cNEK6 competitively binds with ubiquitin E3 ligase BTRC to SNRPA to inhibit its K48 ubiquitination

**Fig. 5:**
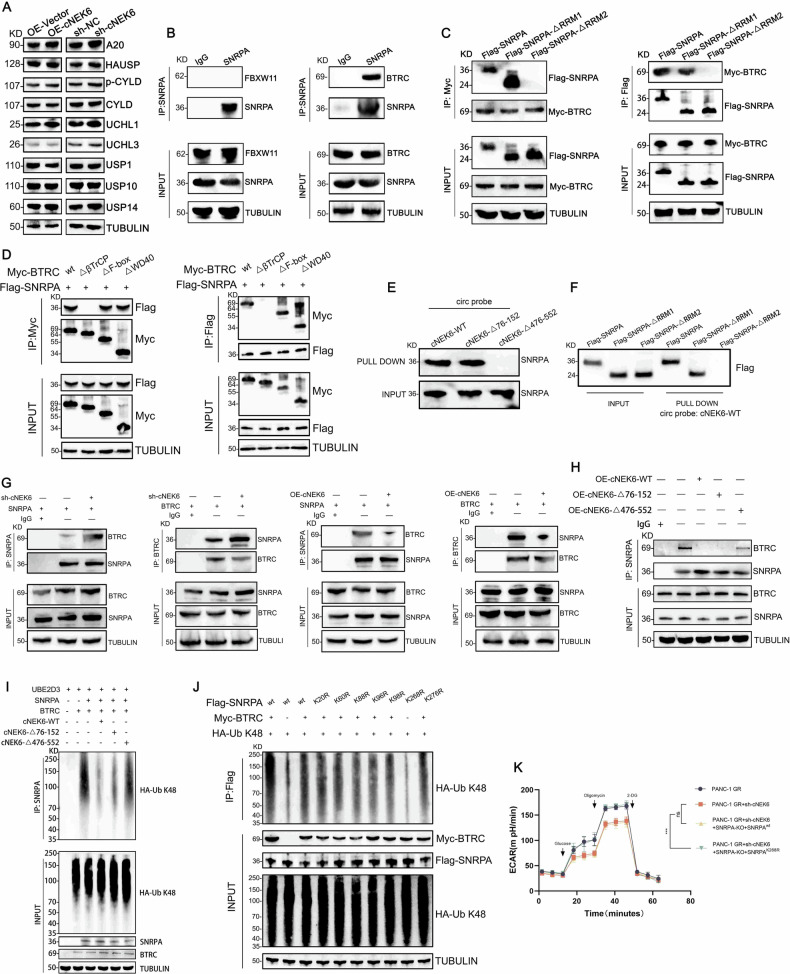
cNEK6 competitively binds with ubiquitin E3 ligase BTRC to SNRPA to inhibit its K48 ubiquitination. **A** The protein levels of deubiquitinases in PANC-1 GR cell lines and control PANC-1 WT cells transfected with sh-cNEK6 and OE-cNEK6. **B** Binding of FBXW11 or BTRC with SNRPA was conducted by COIP in PANC-1 cells. **C** After co-transfecting of Myc-tagged full-length BTRC and full-length or different truncations of SNRPA with FLAG, COIP experiments were performed. **D** After co-transfecting FLAG-tagged full-length SNRPA and full-length or different truncations of BTRC with Myc, COIP experiments were performed. **E** RNA pull-down assay was performed using the wild-type or mutated probe of cNEK6 and wild-type SNRPA. **F** RNA pull-down assay was performed using the wild-type probe of cNEK6 and full-length or different truncations of SNRPA with FLAG. **G** Binding of BTRC with SNRPA was conducted by COIP experiment in PANC-1 GR and PANC-1 WT cell lines transfected with sh-cNEK6 and OE-cNEK6, respectively. **H** Binding of BTRC to SNRPA was conducted by COIP experiment in PANC-1 cells transfected with wild-type or mutant cNEK6. **I** Ubiquitination activities of BTRC, UBE2D3, and SNRPA with wild-type or mutant cNEK6. **J** Ubiquitination of Flag-SNRPA (WT or mutant) in 293 T cells transfected with Myc-BTRC or HAUb-K48. **K** Effect of wild-type or mutant SNRPA on cNEK6 induced changes in ECAR in PANC-1 GR cell lines (*n* = 3).

We tested the expression of deubiquitinating enzymes to explore the mechanism of cNEK6 regulating K48 ubiquitination of SNRPA. The results showed that the protein and mRNA levels of the deubiquitinating enzymes were not affected by cNEK6 (Fig. 5A, Suppl. Fig. 5A). Ubiquitin E3 ligases are crucial for regulating ubiquitination modifications; thus, we focused on E3 ubiquitin ligases that might bind to SNRPA. Using the UbiBrowser database (ubibrowser.ncpsb.org.cn), we identified two E3 ligases, BTRC and FBXW11, that could potentially bind to SNRPA (Suppl. Fig. 5B). After incubating the cell lysates with SNRPA antibodies, we performed CO-IP. The results showed that BTRC, not FBXW11, is bound to SNRPA (Fig. 5B). Next, we constructed a domain-deleted truncated SNRPA and BTRC. CO-IP experiments showed that SNRPA bound to BTRC through the RNA recognition motif (RRM) 2 domain, and BTRC bound to SNRPA through the β-TrCP domain (Fig. 5C, D).

Because BTRC can bind to SNRPA, we measured its expression in OE-cNEK6 and sh-cNEK6 groups. Western blotting and RT-qPCR revealed that the BTRC expression level was not affected by cNEK6 (Suppl. Fig. 5C, D). Because E3 ubiquitin ligases must recognize and bind to substrates to exert their functions, we investigated whether cNEK6 affects the binding of BTRC to SNRPA. According to the CatRabbit database (s.tartaglialab.com/page/catrapid_group), cNEK6 can bind to the RRM2 domain (Suppl. Fig. 5E, F), the same domain BTRC interacts with in SNRPA. We used nucleotide region-deficient cNEK6 mutation probes and previously constructed domain-deletion-truncated SNRPA to verify the binding site of cNEK6 on SNRPA. Through an RNA pull-down assay, we confirmed that cNEK6 binds to the RRM2 domain of SNRPA through the 476-552 nucleotides region (Fig. 5E, F). We considered whether cNEK6 affects the interaction between BTRC and SNRPA since the binding positions of cNEK6 and BTRC on SNRPA were the same. Through CO-IP, we found that OE-cNEK6 inhibited the binding of BTRC to SNRPA, whereas sh-cNEK6 had opposite effects (Fig. 5G). Moreover, only the 476-552 nucleotide region of cNEK6 inhibited the binding of BTRC to SNRPA (Fig. 5H). In vitro ubiquitination experiments further confirmed that BTRC could mediate SNRPA K48 ubiquitination, while cNEK6 could inhibit SNRPA K48 ubiquitination through its 476-552 nucleotide region (Fig. 5I). To explore the specific sites on SNRPA that could be ubiquitinated, we mutated the potential ubiquitinated sites on SNRPA according to UbiBrowser (Suppl. Fig. 5G) and performed CO-IP. The results showed that BTRC mainly led to K48 ubiquitination at the K268 site of SNRPA (Fig. 5J). After knocking out endogenous SNRPA in the PANC-1-GR-sh-cNEK6 group, we supplemented wild-type SNRPA or K268 mutated SNRPA and measured the glycolysis levels. The results showed that cNEK6 deficiency could not inhibit glycolysis in PANC-1-GR cells when K268 was mutated in SNRPA (Fig. 5K, Suppl. Fig. 5H). These findings prove that cNEK6 can competitively bind with BTRC to SNRPA, thereby inhibiting K48 ubiquitination at the K268 site in SNRPA and enhancing glycolysis in PDAC.

### SNRPA activates the mTROC1 pathway by recognizing G-quadruplex structures in PP2Ac

**Fig. 6:**
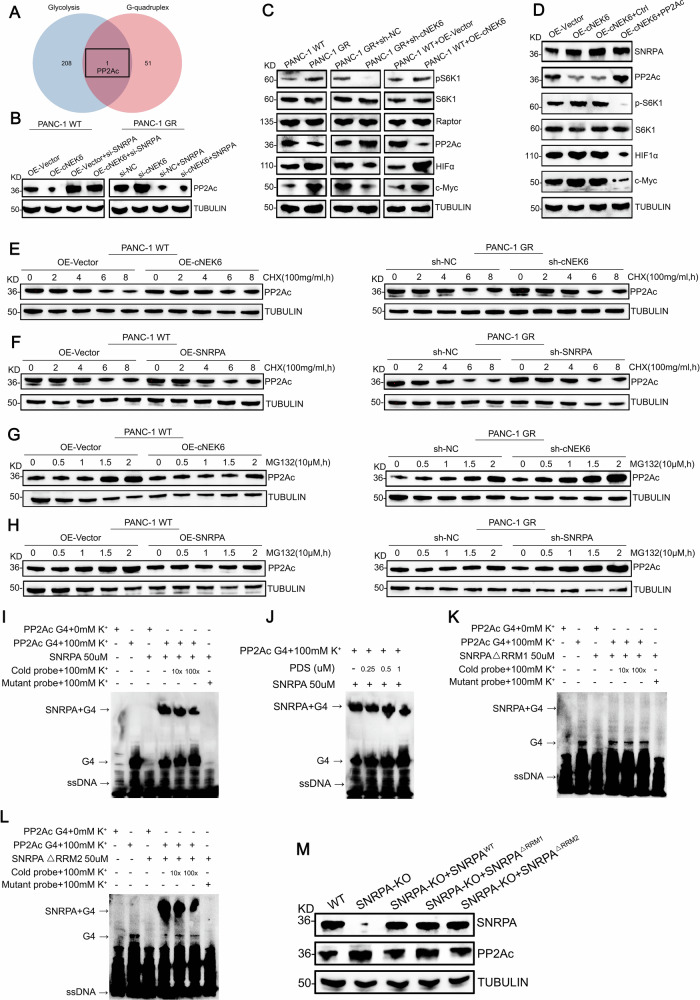
SNRPA activates the mTROC1 pathway by recognizing G-quadruplex structures in PP2Ac. **A** Venn diagram showing the intersection of glycolysis-related genes and genes containing G-quadruplex structures. **B** Protein levels of PP2Ac in OE-Vector/OE-cNEK6 PANC-1 WT cells transfected with or without sh-SNRPA and in sh-NC/sh-cNEK6 PANC-1 GR cells transfected with or without SNRPA. **C** Protein levels of PP2Ac and several downstream genes in the mTORC1 pathway. **D** Protein levels of SNRPA and several downstream genes in the mTORC1 pathway in OE-Vector/OE-cNEK6 PANC-1 WT cells transfected with or without PP2Ac. **E**–**H** After cycloheximide treatment (CHX, 100 μg/mL) (**E**, **F**) or MG132 (10 μM) treatment (**G**, **H**), temporal changes in PP2Ac protein level in PANC-1 GR cell lines, and their control, PANC-1 WT cell lines, with the indicated treatment. **I** EMSA of recombinant SNRPA binding the G4 structure. **J** EMSA shows the effect of PDS at different concentrations on the binding of SNRPA to the G4 structures. **K**, **L** EMSA of different truncations of SNRPA binding to the G4 structure. **M** Protein levels of PP2Ac in PANC-1 cells transfected with wild-type or different truncations of SNRPA.

However, the mechanism by which SNRPA regulates glycolysis remains unclear. SNRPA recognizes and binds to G4 structures [18], which can exist at the 5′ mRNA in several genes and lead to the translational inhibition of the target genes. Therefore, we investigated whether SNRPA regulated the expression of glycolysis-related genes through G4 structures. We collected two gene sets; one involves the genes related to the glycolysis pathway in the Kyoto Encyclopedia of Genes and Genomes database, while the other contains genes identified to have G4 structures [19]. Through the Venny intersection, we screened out a gene, PP2Ac, with a G4 structure and participates in glycolysis regulation (Fig. 6A).

PP2Ac is a key dephosphorylase that regulates glycolysis, and its inhibition can lead to abnormal activation of the mTORC1 pathway and promote glycolysis [24–26]. We tested the effect of the cNEK6-SNRPA axis on PP2Ac expression. Western blotting showed that cNEK6 decreased the protein levels of PP2Ac, whereas SNRPA knockdown reversed this effect (Fig. 6B). cNEK6 deficiency increased the protein level of PP2Ac, while the overexpression of SNRPA attenuated this result (Fig. 6B). RT-qPCR results showed that neither cNEK6 nor SNRPA affect the mRNA level of PP2Ac (Suppl. Fig. 6A). Since the cNEK6-SNRPA axis can regulate PP2Ac expression, it is possible that the cNEK6-SNRPA axis modulates glycolysis through the mTORC1 pathway. Western blotting confirmed that the mTORC1 pathway was abnormally activated in the gemcitabine-resistant groups, whereas cNEK6 knockdown inhibited the activation of the mTORC1 pathway. In contrast, cNEK6 overexpression activated the mTROC1 pathway in wild-type control groups (Fig. 6C). Furthermore, cNEK6 could not activate the mTORC1 pathway after PP2Ac was supplemented in the cNEK6 over-expressed groups (Fig. 6D).

We used CHX and MG132 to clarify the regulatory mechanism of the cNEK6-SNRPA axis in PP2Ac protein expression. The results showed no difference in PP2Ac degradation after the application of CHX, regardless of the expression levels of cNEK6 or SNRPA, indicating that the cNEK6-SNRPA axis did not affect PP2Ac degradation (Fig. 6E, F, Suppl. Fig. 6B, C). However, after inhibiting protein degradation with MG132, cNEK6 or SNRPA knockdown led to a significant accumulation of PP2Ac (Fig. 6G, H, Suppl. Fig. 6D, E), whereas cNEK6 or SNRPA overexpression led to a notable decrease in PP2Ac accumulation (Fig. 6G, H, Suppl. Fig. 6D, E), indicating that the cNEK6-SNRPA axis regulates PP2Ac protein synthesis. Since SNRPA could bind to G4 structures, thereby preventing ribosomes from translating the downstream mRNA of target genes, we designed wild-type probes and mutant probes based on the G4 structures in the 5′mRNA of PP2Ac and performed electrophoretic mobility shift assay (EMSA). The results showed that SNRPA could form a complex with the wild-type G4 of PP2Ac and not with the mutant G4; this complex could be competitively inhibited by cold probes (Fig. 6I). By adding pyridostatin, a compound that specifically recognizes and binds to G4 structures, the complex of SNRPA and the G4 of PP2Ac was competitively inhibited with increasing pyridostatin concentrations (Fig. 6J).

Next, we explored the specific SNRPA domain that could bind to the G4 of PP2Ac. Using the domain-deleted truncated SNRPA, we added G4 probes and performed EMSA. The results showed that SNRPA binds to G4 through the RRM1 domain (Fig. 6K, L). We confirmed this by over-expressing wild-type or domain-deleted truncated SNRPA in the endogenous SNRPA knocked-out cells and found that only the RRM1 domain-deleted SNRPA could not inhibit the protein level of PP2Ac (Fig. 6M).

The above results suggest that the cNEK6-SNRPA axis inhibits the translation of PP2Ac mainly through the binding of SNRPA to the G4 structures of PP2Ac and, therefore, activates the mTORC1 pathway.

### cNEK6 can serve as a biomarker for applying mTORC1 inhibitor to sensitize gemcitabine chemotherapy

**Fig. 7:**
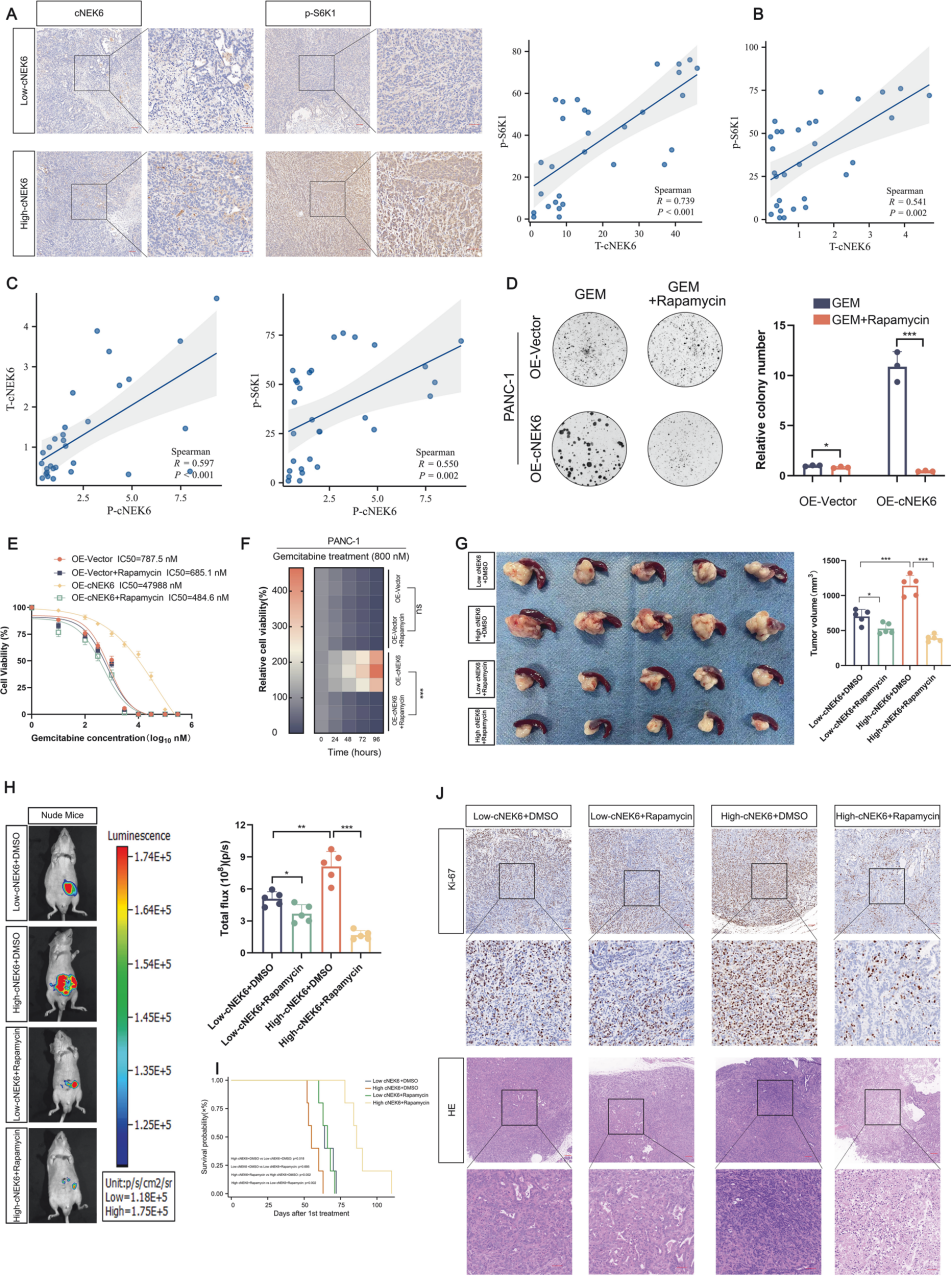
cNEK6 can serve as a biomarker for applying mTORC1 inhibitor to sensitize gemcitabine chemotherapy. **A** Representative ISH images of cNEK6 and immunohistochemistry (IHC) images of p-S6K1 in pancreatic cancer tissues (scale bar: 100 μm). The correlation between the expression levels of cNEK6 and p-S6K1 was calculated based on ISH and IHC scores, respectively (*n* = 30). **B** Correlation between the expression level of cNEK6 as measured by RT-qPCR and the IHC score of p-S6K1 in pancreatic cancer tissues (*n* = 30). **C** Correlation between the expression level of cNEK6 in peripheral blood (P-cNEK6) (RT-qPCR), cNEK6 in pancreatic cancer tissues (T-cNEK6) (RT-qPCR), and p-S6K1 in pancreatic cancer tissues (IHC score) (*n* = 30). **D**–**F** Effect of the mTORC1 inhibitor, rapamycin, on the cNEK6 enhanced proliferative ability (colony formation (**D**), IC50 (**E**), and cell viability (**F**)) of PANC-1 WT lines under gemcitabine treatment. **G** Tumor growth in orthotopic models of nude mice treated with the indicated treatments (*n* = 5). **H** Representative images and statistical analysis of in vivo bioluminescence in orthotopic tumor models subjected to the indicated treatments (*n* = 5). **I** Overall survival of nude mice subjected to the indicated treatments (*n* = 5). **J** Representative images of IHC and hematoxylin and eosin staining in nude mice administered the indicated treatments. Scale bar: 100 µm.

Considering that cNEK6 can activate the mTORC1 pathway in vitro, we further clarified the correlation between cNEK6 and the mTORC1 pathway by collecting PDAC tissues from xenograft tumor models and clinical patients. The expression of cNEK6 was detected by RT-qPCR and ISH, and p-S6K1 was measured by immunohistochemistry (IHC). The results showed that a high cNEK6 expression in PDAC indicated a higher level of p-S6K1 (Fig. 7A, B). Considering the stability of circRNA and its potential as a diagnostic marker, we examined cNEK6 expression in the peripheral blood of clinical patients. cNEK6 isolated from plasma was confirmed as cNEK6 that could be transcribed by random6-mer but not oligo(dT) primers (Suppl. Fig. 7A). We found that the high expression of cNEK6 in peripheral blood also clearly indicated high levels of p-S6K1 and cNEK6 in PDAC tissues (Fig. 7C).

Given that cNEK6 promotes glycolysis by activating the mTORC1 pathway, we used a mTORC1 inhibitor in PDAC cell lines to explore whether it could sensitize gemcitabine chemotherapy in PDAC cells with high cNEK6 expression. As expected, the commercially available mTORC1 inhibitor, rapamycin, significantly inhibited glycolysis in PDAC cells with high cNEK6 expression (Suppl. Fig. 7B, C) and effectively sensitized the cells to gemcitabine chemotherapy (Fig. 7D–F). Furthermore, by detecting the level of cNEK6 in peripheral blood, xenograft tumor models were divided into high- and low-expression cNEK6 groups and gemcitabine significantly reduced tumor size and volume (Fig. 7G, H), and prolonged survival (Fig. 7I) in mice with high cNEK6 expression when the mTORC1 inhibitor was applied. H&E staining and immunohistochemical staining for Ki-67 confirmed that rapamycin enhanced the tumoricidal effect of gemcitabine in high-expression cNEK6 tumors (Fig. 7J). Given that inhibiting SNRPA significantly suppresses glycolysis and PP2Ac supplementation inhibits the mTORC1 pathway in vitro, we investigated whether targeting SNRPA-PP2Ac could elicit chemotherapy sensitization effects similar to those observed with rapamycin in vivo. As a commercial medicament targeting SNRPA or PP2Ac is unavailable, we adopted SNRPA antibodies or supplemented PP2Ac recombinant protein in our in vivo experiments, but the effects were insignificant (Suppl. Fig. 7D, E). Compared to rapamycin, which effectively inhibited the activation of the mTORC1 pathway in tumors (Suppl. Fig. 7F), SNRPA antibodies or PP2Ac recombinant proteins could not effectively modulate the expression of SNRPA or PP2Ac and the mTORC1 pathway in tumors (Suppl. Fig. 7G, H), indicating that unsatisfactory results may partially be due to the degradation or ineffective delivery of antibodies and recombinant proteins in vivo.

The above in vitro and in vivo experimental results revealed that cNEK6 could activate the mTORC1 pathway and serve as a biomarker, suggesting the addition of a mTORC1 inhibitor to gemcitabine chemotherapy to effectively suppress PDAC.

## Discussion

Resistance of pancreatic cancer cells to gemcitabine severely limits chemotherapy efficacy [27]. To overcome chemotherapy resistance, a reliable molecular marker that can indicate the sensitivity of PDAC to gemcitabine and guide the therapeutic schedule for chemotherapy sensitization is urgently required. Based on the stability and convenience of circRNA detection, we discovered that the circRNA cNEK6 can be detected in tumor tissues and peripheral blood of patients with PDAC and that highly expressed cNEK6 accurately indicates PDAC resistance to gemcitabine. Furthermore, cNEK6 enhances glycolysis in PDAC by activating the mTORC1 pathway to confer resistance to gemcitabine. By applying mTORC1 inhibitor, PDAC with high cNEK6 levels was effectively suppressed by treatment with gemcitabine.

Glycolysis is an important intrinsic reason for gemcitabine resistance [7, 28–30]. It competitively inhibits gemcitabine activity by promoting deoxycytidine triphosphate accumulation in PDAC. Thus, glycolysis inhibition in PDAC can be a potential therapeutic strategy for sensitizing tumors to gemcitabine chemotherapy. However, biomarkers are lacking in determining which patients can benefit significantly from glycolysis inhibition. In this study, we confirmed that cNEK6 can serve as a stable indicator of patients with gemcitabine resistance needing glycolysis inhibitors. We discovered that cNEK6 promoted glycolysis by activating the mTORC1 pathway. mTORC1 can enhance glycolysis by regulating two critical transcription factors, HIF1a and Myc, which are responsible for the expression of several key proteins in the glycolytic pathway, such as GLUT1, HK1, HK2, and pyruvate kinase M2 [31–34]. mTORC1 inhibitors can effectively block glycolysis and sensitize patients with high cNEK6 levels in the tumor tissues or peripheral blood to gemcitabine chemotherapy.

Molecules upstream of the mTORC1 pathway, including PI3K/Akt and Mek/Erk, are often abnormally activated in tumors, leading to phosphorylation and activation of the mTORC1 pathway [31]. However, the phosphorylation of mTORC1 is usually reversible; TSC2/TSC1 can dephosphorylate mTORC1 and inhibit its activity [31]. PP2Ac is an important inhibitor of the mTORC1 pathway. Knocking down PP2Ac in colorectal cancer enhances mTORC1 phosphorylation [35]. In T cells and liver cancer, inhibiting PP2Ac activates mTORC1, thereby facilitating glycolysis [24, 25]. In this study, we demonstrated the important regulatory role of PP2Ac on mTORC1 and glycolysis in pancreatic cancer. In pancreatic cancer with high cNEK6 expression, PP2Ac is significantly inhibited, underlying the main reason for mTORC1 activation.

PP2Ac is an important tumor suppressor but the regulatory mechanism of PP2Ac expression, especially in pancreatic cancer, is poorly explored. Protein expression of PP2Ac was significantly decreased in gemcitabine-resistant PDAC groups. PP2Ac can be degraded by MID1-mediated ubiquitination modification [36], but we found that the decrease in PP2Ac protein caused by cNEK6 does not depend on the proteasome degradation pathway but rather inhibits the protein translation of PP2Ac. The 5′ UTR of PP2Ac mRNA contains the G4 structure, characterized by a folded four-stranded conformation formed by G-rich DNA or RNA sequences [37]. G4 in the mRNA can participate in RNA post-transcriptional regulation, including mRNA translation [38, 39]. Aberrant expression of RNA G4-binding proteins can result in inappropriate regulation of target genes containing G4s, thus promoting the proliferation and metastasis of tumors [40]. In this study, we demonstrated that SNRPA, a G4-binding protein abnormally elevated by cNEK6, inhibited the translation of PP2Ac through binding to G4 at the 5′ UTR of PP2Ac mRNA. We identified a novel regulatory mechanism of PP2Ac expression and unveiled the involvement of the G4 structure in gemcitabine resistance, further expanding the role of G4s in cancer.

We also found that cNEK6 regulated glycolysis through its binding protein, SNRPA, rather than as a microRNA sponge or encoding peptide. SNRPA was first discovered as a splicing-related factor. SNRPA, an oncogene, is closely related to tumor progression [21–23]. However, whether SNRPA can facilitate chemotherapy resistance or regulate glycolysis remains unclear. In this study, for the first time, we demonstrated the important role of SNRPA as a G4-binding protein in promoting gemcitabine resistance through glycolysis. We uncovered a new pathway for regulating the ubiquitination of SNRPA. Specifically, we identified BTRC as an E3 ubiquitin ligase for the K48 ubiquitination modification of SNRPA, whereas cNEK6, as a competitor of BTRC binding to SNRPA, prevented BTRC-induced ubiquitination and protein degradation of SNRPA.

Given the importance of the cNEK6-SNRPA-PP2Ac axis for mTORC1 activation, using high cNEK6 expression as an interventional indicator, we confirmed that targeting of SNRPA/mTORC1 or supplementation of PP2Ac in vitro can effectively enhance the sensitivity of PDAC to gemcitabine chemotherapy. The mTORC1 inhibitor, rapamycin, consistently revealed a robust gemcitabine sensitization effect in xenografts with high cNEK6 expression. However, due to the lack of reliable drugs inhibiting SNRPA or supplementing PP2Ac, it is difficult to effectively target the SNRPA-PP2Ac-mTORC1 axis in vivo merely through SNRPA antibodies or PP2Ac recombinant proteins. Designing efficient SNRPA inhibitors or PP2Ac supplementation carriers is a promising task for gemcitabine sensitization. Nanoparticles can effectively deliver siRNAs or DNA/mRNA gene constructs encoding proteins in vivo [41, 42]. Specialized nanoparticles are needed for further clarification of the therapeutic efficacy of inhibiting SNRPA and PP2Ac supplementation in vivo.

In conclusion, this study provides a potential biomarker and therapeutic strategy for sensitizing patients with PDAC to gemcitabine chemotherapy. The high expression of cNEK6 in the tumor tissue and peripheral blood of patients with PDAC strongly suggests gemcitabine resistance and can serve as an indicator of the effectiveness of the combination of a mTORC1 inhibitor and gemcitabine in suppressing PDAC.

## Materials and methods

### Cell culture

Human PDAC cell lines, PANC-1 and PaTu8988t, were purchased from the American Type Culture Collection (ATCC, VA, USA), and gemcitabine-resistant PANC-1 (PANC-1-GR) and gemcitabine-resistant PaTu8988t (PaTu8988t-GR) cells were established in our laboratory by culturing with repeated gemcitabine induction. All cell lines were cultured in Dulbecco’s Modified Eagle Medium (DMEM) supplemented with 10% fetal bovine serum and were free of mycoplasma based on testing using the Universal Mycoplasma Detection Kit.

### Patient information and tissue specimens

Histopathologically diagnosed pancreatic cancer tissues from gemcitabine-treated patients of PADC were obtained from Fujian Union Hospital.

### Cell viability assay

Cell proliferation was assessed using Cell Counting Kit-8 Dojindo, Kumamoto, Japan), and absorbance was measured spectrophotometrically at 450 nm using a microplate reader (Tecan Trading AG, Switzerland). All absorbance values were normalized to those of the blank wells, and the cell viability was normalized to those wells treated with dimethyl sulfoxide (carrier). The half-maximal inhibitory concentration (IC50) was calculated 48 h after treatment.

### Colony formation assay

Cells (800 cells/well) were seeded in 6-well plates. After 24 h, the IC50 of gemcitabine for the respective cell lines was applied for 48 h. The cells were cultured for 10 days. Cell colonies were fixed in 4% formaldehyde and stained with 0.1% crystal violet (Sigma, St. Louis, MO, USA). The number of colonies was determined using the ImageJ software.

### Seahorse analyses

PANC-1 and PaTu8988t cells (10,000 cells/well) were seeded in 24-well cell culture plates (Seahorse Biosciences, North Billerica, MA) in DMEM supplemented with 4.5 g/L glucose (Gibco) and 2 mM glutamine (Gibco), and incubated at 37 °C, overnight in a 5% CO_2_ incubator. For the glycolysis stress test, the ECAR was measured in response to sequential injections of glucose (10 mM), oligomycin (1 μM), and oligomycin (2-DG, 1 μM).

### Glucose uptake assay

Cells (10,000 cells/well) were seeded into a 96-well plate. After 10 h incubation, cells were washed thrice with PBS and were glucose-starved by incubating with 100 μL Krebs–Ringer–Phosphate–HEPES buffer containing 2% BSA for 40 min. Next, 2-DG was added and the cells were incubated for 20 min. The cells were lysed with an extraction buffer and heated at 85 °C for 40 min. The cell lysate was neutralized using a neutralization buffer. After centrifugation, glucose uptake was measured in the supernatant using a microplate reader at 412 nm (Biovision). Data were normalized to the cell number.

### Pyruvate activity assay

Cells (5 × 10^5^) were collected and extracted using the Pyruvate Assay Buffer (Biovision). After centrifugation, the supernatant was analyzed using a Pyruvate Colorimetric Assay kit (Biovision) at 570 nm in a microplate reader. The results were normalized to the cell number.

### Lactate production

Cells (1 × 10^5^) were seeded into a 12-well plate in DMEM containing 10% fetal bovine serum overnight, and the medium was replaced with DMEM without fetal bovine serum. After 1 h of incubation, the supernatant was collected for lactate production measurement (Biovision) via a microplate reader at 450 nm and normalized to the cell number.

### ATP level detection

Cells (5 × 10^5^) were collected and extracted using an ATP Assay Buffer (Biovision). After centrifugation, the supernatant was analyzed using an ATP Colorimetric Assay Kit (Biovision) at 570 nm in a microplate reader. Data were normalized to the cell number.

### RNA extraction and qRT-PCR analysis

Total RNA from PDAC tissues or cell lines was isolated using TRIzol Reagent (Invitrogen, Carlsbad, CA, USA), according to the manufacturer’s protocol. Reverse transcription was performed using the Prime Script RT Reagent Kit (Takara, Dalian, China). Bulge-loop miRNA RT-qPCR primers were used to determine the miRNA levels. Real-time PCR was performed using the StepOnePlus Real-Time PCR System (Thermo Fisher Scientific). The program settings for the temperature cycling were as instructed by the manufacturer. The relative circRNA and mRNA expression levels were normalized to those of TUBULIN using the 2^−DDCT^ method.

### Sample preparation and RNA isolation

We collected and detected circRNA from peripheral blood following a previously described protocol [43, 44]. In brief, 4 mL of peripheral blood were collected in BD Vacutainer tubes (EDTA-K2 acted as anticoagulation) (BD, New Jersey, USA), then plasma samples were isolated and centrifuged following the two-step protocol: the tubes were centrifuged at 800×*g* for 10 min, and 1 mL of the supernatant plasma were transferred to RNase-free tubes (AXYGEN, JIANGSU, China) and centrifuged again at 16,000×*g* for 3 min. The supernatant was transferred to new RNase-free tubes and stored at −80 °C as the prepared sample until use. CircRNA was isolated from 400 μL of the prepared sample using a miRNeasy® Serum/Plasma Advanced Kit (Qiagen) following the manufacturer’s instructions.

### RNA-seq analysis

The treated PDAC cells were subjected to RNA-Seq analysis using SeqHealth Technology (Wuhan, China). Total RNAs were extracted from PDAC cells using the TRIzol reagent (Invitrogen, Carlsbad, CA, USA) and underwent stranded RNA sequencing library preparation using the KC-Digital Stranded mRNA Library Prep Kit and the circRNA library for Illumina (Seq Health Technology, Wuhan, China), according to the manufacturer’s instructions. The kit eliminated duplication bias in the PCR and sequencing steps using a unique molecular identifier of eight random bases to label the pre-amplified cDNA molecules. Library products corresponding to 200–500 bp were enriched, quantified, and sequenced on a DNBSEQ-T7 sequencer (MGI Tech, Shenzhen, China) using the PE150 model.

### Screening for differentially expressed genes (DEGs) and enrichment analysis

The “limma” R package was used to infilter DEGs. A fold change >2 or <0.5 and a *P*-value < 0.05 was set as the threshold for a significant differential expression. Gene Ontology functional annotation and Kyoto Encyclopedia of Genes and Genomes pathway analysis of DEGs were conducted using the “clusterProfiler” R package.

### RNase R treatment

Total RNA was incubated for 30 min at 37 °C with or without 3 U/μg of RNase R (Geenseed, Guangzhou, China), according to the manufacturer’s instructions, and the expression levels of indicated RNA were determined using qRT-PCR.

### Actinomycin D assay

The cells were treated with 2 μg/mL of actinomycin D (Sigma–Aldrich) for different durations. RNA expression levels were determined using RT-qPCR.

### Fluorescence in situ hybridization (FISH)

The specific fluorescently labeled circ-NEK6 FISH probes were designed and synthesized by Servicebio (Wuhan, China). After fixation and permeabilization, samples were hybridized with the probes in a hybridization buffer at 37 °C overnight. The hybridization buffer was then gradually washed off with 4× SSC (including 0.1% Tween-20), 2× SSC, and 1× SSC at 42 °C. Nuclei were counterstained with DAPI. All images were obtained using a Nikon A1Si Laser Scanning confocal microscope (Nikon Instruments Inc., Tokyo, Japan).

### Agarose gel electrophoresis

Nucleic acid samples were loaded into 2% (w/v) agarose gels and separated by electrophoresis in a tris-acetate–EDTA running buffer at 120 V for 30 min. The gel images were visualized using a ChemiDoc MP Imaging System (Bio-Rad, CA, USA).

### Western blot analysis

Proteins were extracted from PDAC cells and tumor tissues using RIP assay buffer (Solarbio, Beijing, China) supplemented with proteinase and phosphatase inhibitors. Protein concentration was determined using the bicinchoninic acid reagent (Beyotime, Beijing, China). The proteins were separated on sodium dodecyl sulfate-polyacrylamide gels and transferred into polyvinylidene difluoride membranes (Merck Millipore). After blocking in 5% skim powdered milk for 1 h, the membranes were incubated with primary antibodies overnight at 4 °C, then with secondary antibodies at room temperature for 1 h. Targeted proteins were measured using the Pierce ECL Western Blotting Kit (Thermo Fisher Scientific, MA, US) with a ChemiDoc MP Imaging System (Bio-Rad, CA, USA). The following primary antibodies were used: anti-SNRPA (ET7107-98), anti-PP2Ac (13482-1-AP), anti-AGO2 (ET1702-39), anti-BTRC (EM1706-79), anti-Ubiquitin (ab19247), anti-K48 Ubiquitin (ab140601), anti-K63 Ubiquitin (ab179434), anti-HIF1a (ab1), anti-c-Myc (ab32072). anti-S6K1 (ab32529), anti-p-S6K1 (ab59208), anti-Raptor (ab40768), anti-Tubulin (ab6046), anti-Flag tag (ab205606), anti-Myc tag (ab32), anti-FBXW11 (DF13009).

### RIP assay

RIP assay was performed using the Magna RIP RNA-binding protein immunoprecipitation kit (Merck Millipore, MA, USA) according to the manufacturer’s protocol. Cells (5 × 10^7^) were lysed in a RIP lysate buffer containing protease and RNase inhibitors. The lysates were incubated with IgG (Abclonal Rabbit Control IgG AC005), anti-SNRPA, and anti-AGO2 antibody-coated beads (Millipore) at 4 °C overnight. Next, the RNA-protein complexes were isolated by incubating cell lysates with the protein A/G magnetic beads at 4 °C for 1 h. After proteinase digestion, the RNAs bound to specific proteins were extracted using phenol/chloroform/isoamyl alcohol (125:24:1) (Solarbio) and reverse-transcribed to cDNA. RNA levels were detected using qRT-PCR.

### RNA pull-down assay

The BersinBio RNA Antisense Purification kit (catalog No. Bes5103; BersinBio, China) was used for the RNA pull-down assay. A biotin-labeled circNEK6 probe was synthesized by Bersin Bio (Guangzhou, China). After, cross-linked cells were lysed, sonicated, and hybridized with the probe for 4 h at 37 °C. The hybridization mixture was then treated with magnetic beads for 1 h. Bound proteins were eluted, collected, and prepared for Western blotting, silver staining, and mass spectrometry.

### Immunofluorescence

Cells were fixed using 4% paraformaldehyde for 15 min, permeabilized with 0.2% Triton X-100 for 10 min, and blocked with 5% bovine serum albumin for 1 h. Primary antibodies with cells were incubated overnight at 4 °C, followed by incubation with fluorescent secondary antibodies at room temperature for 1 h. Cells were mounted after staining with DAPI. All images were obtained using a Nikon A1Si Laser Scanning confocal microscope (Nikon Instruments Inc., Tokyo, Japan).

### Immunohistochemistry (IHC)

Tumor tissue sections were incubated with the indicated primary antibodies overnight at 4 °C, followed by incubation with secondary antibodies at room temperature for 30 min. Next, the sections were stained with DAB solution for 10 min. The staining intensity score was classified into 0 (negative), 1 (weak), 2 (moderate), or 3 (strong). The score for the number of positive tumor cells was classified as 1 (0–25%), 2 (26–50%), 3 (51–75%), or 4 (76–100%). The total IHC score was calculated by multiplying the staining intensity and positivity scores. The positive percentage was calculated using the ‘Trainable Weka Segmentation’ in Image J software, a plugin that can recognize positively or negatively stained cells, according to the previous study.^45^ IHC staining of tissue sections was independently evaluated by two experienced pathologists.

### CircRNA in situ hybridization (RNA-ISH)

A specific digoxin-labeled circRNA-NEK6 probe was designed and synthesized by Servicebio (Wuhan, China). The tumor samples were fixed with formalin, embedded in paraffin, and sectioned into 6-µm slides. ISH was conducted using the Enhanced Sensitive ISH Detection Kit I (BOSTER, Wuhan, China) according to the manufacturer’s protocol. Deparaffinization and rehydration of the sections were performed as described for IHC. The criteria for the staining intensity score and calculation of the total ISH score of circNEK6 were the same as those described for the calculation of the IHC score above.

### Animal experiments

We housed and fed 4–6-week-old male athymic BALB/c nude mice (SLAC Laboratory Animal Co., Ltd., Shanghai, China) under standard pathogen-free conditions. For the patient-derived tumor xenograft model, tumor tissues from patients with PDAC were isolated, disaggregated into approximately 1–2 mm^3^ tissue blocks, and subcutaneously implanted into the right flank of the cultured mice. After 2 weeks, the tumor tissues from the mice were isolated, the expression level of circNEK6 was measured, and the tumors were divided into high/low circNEK6 groups. After dissociating the tumor tissues into cell suspensions, 50 µL of the tumor cells (1 × 10^8^ cells/mL) were implanted orthotopically in the pancreatic duct of the 4–6-week-old male BALB/c nude mice (stably expressing luciferase).

For the cell line-derived xenograft (CDX) model, 50 µL PANC-1 cells (1 × 10^8^ cells/mL), transfected with vector or circNEK6, were orthotopically injected into the pancreatic duct of 4–6-week-old male BALB/c nude mice (stably expressing luciferase).

After the tumor volume reached 70–100 mm^3^, the mice were treated with gemcitabine (30 mg/kg/mouse daily via tail vein injection). The mice were sacrificed after 4 weeks of treatment. Tumor growth was visualized using the in vivo imaging system after d-luciferin injection, and the fluorescence intensity was quantified using the total photon flux (photons/s). Tumor volume (mm^3^) was calculated as (*L* × *W*^2^)/2, where *L* and *W* represent the longest and perpendicular axis, respectively.

### Statistical analysis

The Student’s *t*-test and one-way analysis of variance were used in comparing the differences among the groups. Pearson correlation analysis was applied for the measurement of correlations. The overall survival and progression-free survival rates was calculated using Kaplan–Meier method, the significance was evaluated with the log-rank test. *P*-value < 0.05 was considered to indicate a statistically significant result. *P* values are presented as ns *P* > 0.05, ** *P* < 0.01, and ***P < 0.001. GraphPad Prism 9.0 and SPSS 23.0 were applied for statistical analysis.

## Supplementary information

SUPPLEMENTAL FIGURE

Original Western Blot

## Data Availability

The datasets used in the current study are available from the corresponding author on reasonable request.

## References

[1] Rahib L, Smith BD, Aizenberg R, Rosenzweig AB, Fleshman JM, Matrisian LM. Projecting cancer incidence and deaths to 2030: the unexpected burden of thyroid, liver, and pancreas cancers in the United States. Cancer Res. 2014;74:2913–21. 24840647 10.1158/0008-5472.CAN-14-0155

[2] Chen S, Yang C, Wang Z-W, Hu J-F, Pan J-J, Liao C-Y, et al. CLK1/SRSF5 pathway induces aberrant exon skipping of METTL14 and cyclin L2 and promotes growth and metastasis of pancreatic cancer. J Hematol Oncol. 2021;14:60. 33849617 10.1186/s13045-021-01072-8 PMC8045197

[3] Li G, Liao C, Chen J, Wang Z, Zhu S, Lai J, et al. Targeting the MCP‐GPX4/HMGB1 axis for effectively triggering immunogenic ferroptosis in pancreatic ductal adenocarcinoma. Adv Sci. 2024;11:e2308208. 10.1002/advs.202308208 PMC11151063 38593415

[4] Wang ZW, Pan JJ, Hu JF, Zhang JQ, Huang L, Huang Y, et al. SRSF3-mediated regulation of N6-methyladenosine modification-related lncRNA ANRIL splicing promotes resistance of pancreatic cancer to gemcitabine. Cell Rep. 2022;39:110813. 35545048 10.1016/j.celrep.2022.110813

[5] Chen ZW, Hu JF, Wang ZW, Liao CY, Kang FP, Lin CF, et al. Circular RNA circ-MTHFD1L induces HR repair to promote gemcitabine resistance via the miR-615-3p/RPN6 axis in pancreatic ductal adenocarcinoma. J Exp Clin Cancer Res. 2022;41:153. 35459186 10.1186/s13046-022-02343-z PMC9034615

[6] Yu T, Wang Y, Fan Y, Fang N, Wang T, Xu T, et al. CircRNAs in cancer metabolism: a review. J Hematol Oncol. 2019;12:90. 31484561 10.1186/s13045-019-0776-8 PMC6727394

[7] Shukla SK, Purohit V, Mehla K, Gunda V, Chaika NV, Vernucci E, et al. MUC1 and HIF-1alpha signaling crosstalk induces anabolic glucose metabolism to impart gemcitabine resistance to pancreatic cancer. Cancer Cell. 2017;32:71–87.e7. 28697344 10.1016/j.ccell.2017.06.004 PMC5533091

[8] Ghanbari Movahed Z, Rastegari-Pouyani M, Mohammadi MH, Mansouri K. Cancer cells change their glucose metabolism to overcome increased ROS: one step from cancer cell to cancer stem cell? Biomed Pharmacother. 2019;112:108690. 30798124 10.1016/j.biopha.2019.108690

[9] Nakao K, Minato N, Uemoto S, editors. Innovative medicine: basic research and development. Tokyo: Springer; 2015. 29787043

[10] Pisignano G, Michael DC, Visal TH, Pirlog R, Ladomery M, Calin GA. Going circular: history, present, and future of circRNAs in cancer. Oncogene. 2023;42:2783–2800. 37587333 10.1038/s41388-023-02780-w PMC10504067

[11] Ma S, Kong S, Wang F, Ju S. CircRNAs: biogenesis, functions, and role in drug-resistant tumours. Mol Cancer. 2020;19:119. 32758239 10.1186/s12943-020-01231-4 PMC7409473

[12] Zhang X, Wang S, Wang H, Cao J, Huang X, Chen Z, et al. Circular RNA circNRIP1 acts as a microRNA-149-5p sponge to promote gastric cancer progression via the AKT1/mTOR pathway. Mol Cancer. 2019;18:20. 30717751 10.1186/s12943-018-0935-5 PMC6360801

[13] Bi W, Huang J, Nie C, Liu B, He G, Han J, et al. CircRNA circRNA_102171 promotes papillary thyroid cancer progression through modulating CTNNBIP1-dependent activation of β-catenin pathway. J Exp Clin Cancer Res. 2018;37:275. 30424816 10.1186/s13046-018-0936-7 PMC6234664

[14] Zeng Z, Zhao Y, Chen Q, Zhu S, Niu Y, Ye Z, et al. Hypoxic exosomal HIF-1α-stabilizing circZNF91 promotes chemoresistance of normoxic pancreatic cancer cells via enhancing glycolysis. Oncogene. 2021;40:5505–17. 34294845 10.1038/s41388-021-01960-w

[15] Zhao Q, Zhu Z, Xiao W, Zong G, Wang C, Jiang W, et al. Hypoxia-induced circRNF13 promotes the progression and glycolysis of pancreatic cancer. Exp Mol Med. 2022;54:1940–54. 36369467 10.1038/s12276-022-00877-y PMC9723180

[16] Guan H, Luo W, Liu Y, Li M. Novel circular RNA circSLIT2 facilitates the aerobic glycolysis of pancreatic ductal adenocarcinoma via miR-510-5p/c-Myc/LDHA axis. Cell Death Dis. 2021;12:645. 34168116 10.1038/s41419-021-03918-y PMC8225611

[17] Guiro J, O’Reilly D. Insights into the U1 small nuclear ribonucleoprotein complex superfamily. WIREs RNA. 2014;6:79–92. 25263988 10.1002/wrna.1257

[18] Bolduc F, Turcotte M-A, Perreault J-P. The small nuclear ribonucleoprotein polypeptide A (SNRPA) binds to the G-quadruplex of the BAG-1 5′UTR. Biochimie. 2020;176:122–7. 32629040 10.1016/j.biochi.2020.06.013

[19] Cammas A, Millevoi S. RNA G-quadruplexes: emerging mechanisms in disease. Nucleic Acids Res. 2017;45:1584–95. 28013268 10.1093/nar/gkw1280 PMC5389700

[20] Niu K, Xiang L, Jin Y, Peng Y, Wu F, Tang W, et al. Identification of LARK as a novel and conserved G-quadruplex binding protein in invertebrates and vertebrates. Nucleic Acids Res. 2019;47:7306–20. 31165881 10.1093/nar/gkz484 PMC6698653

[21] Dou N, Yang D, Yu S, Wu B, Gao Y, Li Y. SNRPA enhances tumour cell growth in gastric cancer through modulating NGF expression. Cell Prolif. 2018;51:e12484. 30039889 10.1111/cpr.12484 PMC6528855

[22] Liu J, Li J, Su Y, Ma Z, Yu S, He Y. Circ_0009910 sServes as miR-361-3p sponge to promote the proliferation, metastasis, and glycolysis of gastric cancer via regulating SNRPA. Biochem Genet. 2022;60:1809–24. 35098410 10.1007/s10528-021-10168-2

[23] Mo Z, Li R, Cao C, Li Y, Zheng S, Wu R, et al. Splicing factor SNRPA associated with microvascular invasion promotes hepatocellular carcinoma metastasis through activating NOTCH1/Snail pathway and is mediated by circSEC62/miR‐625‐5p axis. Environ Toxicol. 2023;38:1022–37. 36715182 10.1002/tox.23745

[24] Yao L, Xuan Y, Zhang H, Yang B, Ma X, Wang T, et al. Reciprocal REGγ-mTORC1 regulation promotes glycolytic metabolism in hepatocellular carcinoma. Oncogene. 2020;40:677–92. 33230243 10.1038/s41388-020-01558-8

[25] Yu X, Teng X-L, Wang F, Zheng Y, Qu G, Zhou Y, et al. Metabolic control of regulatory T cell stability and function by TRAF3IP3 at the lysosome. J Exp Med. 2018;215:2463–76. 30115741 10.1084/jem.20180397 PMC6122976

[26] Chen X, Feng L, Li S, Long D, Shan J, Li Y. TGF-β1 maintains Foxp3 expression and inhibits glycolysis in natural regulatory T cells via PP2A-mediated suppression of mTOR signaling. Immunol Lett. 2020;226:31–37. 32598969 10.1016/j.imlet.2020.06.016

[27] Conroy T, Castan F, Lopez A, Turpin A, Ben Abdelghani M, Wei AC, et al. Five-year outcomes of FOLFIRINOX vs gemcitabine as adjuvant therapy for pancreatic cancer. JAMA Oncol. 2022;8:1571–8. 36048453 10.1001/jamaoncol.2022.3829 PMC9437831

[28] Xu F, Huang M, Chen Q, Niu Y, Hu Y, Hu P, et al. LncRNA HIF1A-AS1 promotes gemcitabine resistance of pancreatic cancer by enhancing glycolysis through modulating the AKT/YB1/HIF1α pathway. Cancer Res. 2021;81:5678–91. 34593522 10.1158/0008-5472.CAN-21-0281

[29] Xi Y, Yuan P, Li T, Zhang M, Liu M-F, Li B. hENT1 reverses chemoresistance by regulating glycolysis in pancreatic cancer. Cancer Lett. 2020;479:112–22. 32200037 10.1016/j.canlet.2020.03.015

[30] Feng M, Xiong G, Cao Z, Yang G, Zheng S, Qiu J, et al. LAT2 regulates glutamine-dependent mTOR activation to promote glycolysis and chemoresistance in pancreatic cancer. J Exp Clin Cancer Res. 2018;37:274. 30419950 10.1186/s13046-018-0947-4 PMC6233565

[31] Saxton RA, Sabatini DM. mTOR signaling in growth, metabolism, and disease. Cell. 2017;168:960–76. 28283069 10.1016/j.cell.2017.02.004 PMC5394987

[32] Szwed A, Kim E, Jacinto E. Regulation and metabolic functions of mTORC1 and mTORC2. Physiol Rev. 2021;101:1371–426. 33599151 10.1152/physrev.00026.2020 PMC8424549

[33] Orozco JM, Krawczyk PA, Scaria SM, Cangelosi AL, Chan SH, Kunchok T, et al. Dihydroxyacetone phosphate signals glucose availability to mTORC1. Nat Metab. 2020;2:893–901. 32719541 10.1038/s42255-020-0250-5 PMC7995735

[34] Li L, Liang Y, Kang L, Liu Y, Gao S, Chen S, et al. Transcriptional regulation of the Warburg effect in cancer by SIX1. Cancer Cell. 2018;33:368–.e7. 29455928 10.1016/j.ccell.2018.01.010

[35] Zhang Y, Wang X, Qin X, Wang X, Liu F, White E, et al. PP2AC level determines differential programming of p38-TSC-mTOR signaling and therapeutic response to p38-targeted therapy in colorectal cancer. EBioMedicine. 2015;2:1944–56. 26844273 10.1016/j.ebiom.2015.11.031 PMC4703732

[36] Liu E, Knutzen CA, Krauss S, Schweiger S, Chiang GG. Control of mTORC1 signaling by the Opitz syndrome protein MID1. Proc Natl Acad Sci USA. 2011;108:8680–5. 21555591 10.1073/pnas.1100131108 PMC3102420

[37] Huppert JL, Balasubramanian S. Prevalence of quadruplexes in the human genome. Nucleic Acids Res. 2005;33:2908–16. 15914667 10.1093/nar/gki609 PMC1140081

[38] Haeusler AR, Donnelly CJ, Periz G, Simko EAJ, Shaw PG, Kim M-S, et al. C9orf72 nucleotide repeat structures initiate molecular cascades of disease. Nature. 2014;507:195–200. 24598541 10.1038/nature13124 PMC4046618

[39] Reddy K, Zamiri B, Stanley SYR, Macgregor RB, Pearson CE. The disease-associated r(GGGGCC) repeat from the C9orf72 gene forms tract length-dependent uni- and multimolecular RNA G-quadruplex structures. J Biol Chem. 2013;288:9860–6. 23423380 10.1074/jbc.C113.452532 PMC3617286

[40] Lucá R, Averna M, Zalfa F, Vecchi M, Bianchi F, Fata GL, et al. The fragile X protein binds mRNAs involved in cancer progression and modulates metastasis formation. EMBO Mol Med. 2013;5:1523–36. 24092663 10.1002/emmm.201302847 PMC3799577

[41] Luo M, Lee LKC, Peng B, Choi CHJ, Tong WY, Voelcker NH. Delivering the promise of gene therapy with nanomedicines in treating central nervous system diseases. Adv Sci. 2022;9:e2201740. 10.1002/advs.202201740 PMC9475540 35851766

[42] Chen ZW, Kang FP, Xie CK, Liao CY, Li G, Wu YD, et al. A novel trojan horse nanotherapy strategy targeting the cPKM‐STMN1/TGFB1 axis for effective treatment of intrahepatic cholangiocarcinoma. Adv Sci. 2023;10:e2303814. 10.1002/advs.202303814 PMC10646249 37789644

[43] Nanishi K, Konishi H, Shoda K, Arita T, Kosuga T, Komatsu S, et al. Circulating circERBB2 as a potential prognostic biomarker for gastric cancer: an investigative study. Cancer Sci. 2020;111:4177–86. 32896032 10.1111/cas.14645 PMC7648027

[44] Lin J, Cai D, Li W, Yu T, Mao H, Jiang S, et al. Plasma circular RNA panel acts as a novel diagnostic biomarker for colorectal cancer. Clin Biochem. 2019;74:60–68. 31669510 10.1016/j.clinbiochem.2019.10.012

[45] Ignacio A-C, Verena K, Curtis R, Kevin WE, Johannes S, Albert C, et al. Trainable Weka Segmentation: a machinelearning tool for microscopy pixel classification. Bioinformatics. 2017;33(15):2424–6. 10.1093/bioinformatics/btx180 28369169

