# Supplementary material for: cNEK6 induces gemcitabine resistance by promoting glycolysis in pancreatic ductal adenocarcinoma via the SNRPA/PPA2c/mTORC1 axis
Source: Cell Death Dis. 2024 Oct 11;15(10):742. doi: 10.1038/s41419-024-07138-y (PMC11470042; doi:10.1038/s41419-024-07138-y)
Supplement: Supplementary file 1 — SUPPLEMENTAL FIGURE [file 41419_2024_7138_MOESM1_ESM.pdf]

Supplementary Figure Legends

Supplementary FIG. 1.

A. Volcano diagram of differentially expressed genes (DEGs) in the indicated gene set. **B.** Schematic representation of the genomic region of cNEK6 and its cyclization. **C.** Agarose gel electrophoresis images showing the amplification of cNEK6 and NEK6 in both complementary (cDNA) and genomic DNA (gDNA) from PANC-1 and PaTu8988t cells using convergent (blue) and divergent (red) primers. **D.** Agarose gel electrophoresis shows the expression of cNEK6 and NEK6 after RNase R treatment of PANC-1 and PaTu8988t cells. **E.** Curves of fluorescence intensities at the position marked by a white line in the merged image in Figure 1D. **F.** cNEK6 RNA levels in gemcitabine-resistant groups (PANC-1 GR and PaTu8988t GR) transfected with sh-NC or sh-cNEK6, and in their control groups (PANC-1 WT and PaTu8988t WT) after transfection with OE-Vector or OE-cNEK6 (n = 3).

Supplementary FIG. 2.

A. DEGs between OE-cNEK6 and OE-Vector PANC-1 cells. **B**. GSEA based on DEGs between OE-cNEK6 and OE-Vector PANC-1 cells. **C.** Statistical analysis of colony formation is shown in Figure 2G.

Supplementary FIG. 3.

A. IRES elements in cNEK6 predicted using the CircBANK database (www.circbank.cn/). **B.** The most potent binding protein of cNEK6 predicted using the RBPDB database (rbpdb.ccbr.utoronto.ca/). **C**. mRNA levels of SNRPA in PANC-1 GR cell lines, and PANC-1 WT cells, transfected with sh-cNEK6 and OE-cNEK6, respectively (n = 3).

Supplementary FIG. 4.

A. Changes in SNRPA protein levels measured in Figure 4A were quantified using line charts (n = 3). **B.** Changes in SNRPA protein levels measured in Figure 4B were quantified using line charts (n = 3). **C.** Ubiquitination of SNRPA in gemcitabine-resistant (PANC-1 GR and PaTu8988t GR) and control groups (PANC-1 WT and PaTu8988t WT) transfected with sh-cNEK6 and OE-cNEK6, respectively. Western blot analysis was performed using antibodies against K63-Ub.

Supplementary FIG. 5.

A. mRNA levels of deubiquitinases in PANC-1 GR cell lines and control PANC-1 WT cells transfected with sh-cNEK6 and OE-cNEK6 (n = 3). **B**. Potential ubiquitin E3 ligases of SNRPA predicted in the UbiBrowser database (ubibrowser.ncpsb.org.cn). **C**. Protein levels of BTRC under the treatment of OE-cNEK6 or sh-cNEK6. **D.** mRNA levels of BTRC under the treatment of OE-cNEK6 or sh-cNEK6 (n = 3). **E-F.**

Potential binding sites of SNRPA in cNEK6 (E) and cNEK6 in SNRPA (F) were predicted using the catRAPID database (s.tartaglialab.com/page/catrapid_group). **G.** Potential ubiquitination sites regulated by BTRC in SNRPA were predicted using the UbiBrowser database. **H.** Effect of wild-type or mutant SNRPA on cNEK6-induced changes in glucose uptake, pyruvate levels, lactate production, and ATP levels in PANC-1 GR cell lines (n = 3).

Supplementary FIG. 6.

A. mRNA levels of PP2Ac in OE-Vector/OE-cNEK6 PANC-1 WT cells transfected with or without sh-SNRPA and in sh-NC/sh-cNEK6 PANC-1 GR cells transfected with or without SNRPA. **B.** Changes in PP2Ac protein levels measured in Figure 6E were quantified using line charts (n = 3). **C.** Changes in PP2Ac protein levels measured in Figure 6F were quantified using line charts (n = 3). **D.** Changes in PP2Ac protein levels measured in Figure 6G were quantified using line charts (n = 3). **E.** Changes in the PP2Ac protein levels measured in Figure 6H were quantified using line charts (n = 3).

Supplementary FIG. 7.

A. cDNA of the plasma cNEK6 and GAPDH was transcribed with random 6-mer or oligo (dT) primers and then detected by qRT-PCR

(n = 3). **B**. Effect of rapamycin on OE-cNEK6 induced changes in ECAR in PANC-1 WT cell lines (n = 3). **C**. Effect of rapamycin on OE-cNEK6 induced changes in glucose uptake, pyruvate levels, lactate production, and ATP levels in PANC-1 WT cell lines (n = 3). **D**. Tumor growth in orthotopic models of nude mice treated with the IgG or anti-SNRPA (n = 5). **E**. Tumor growth in orthotopic models of nude mice treated with the Trehalose or PP2Ac recombinant proteins (n = 5). **F-H**. The effects of rapamycin (F), anti-SNRPA (G), and PP2Ac recombinant proteins (H), on the expression of SNRPA, PP2Ac, and mTORC1 relative pathway.

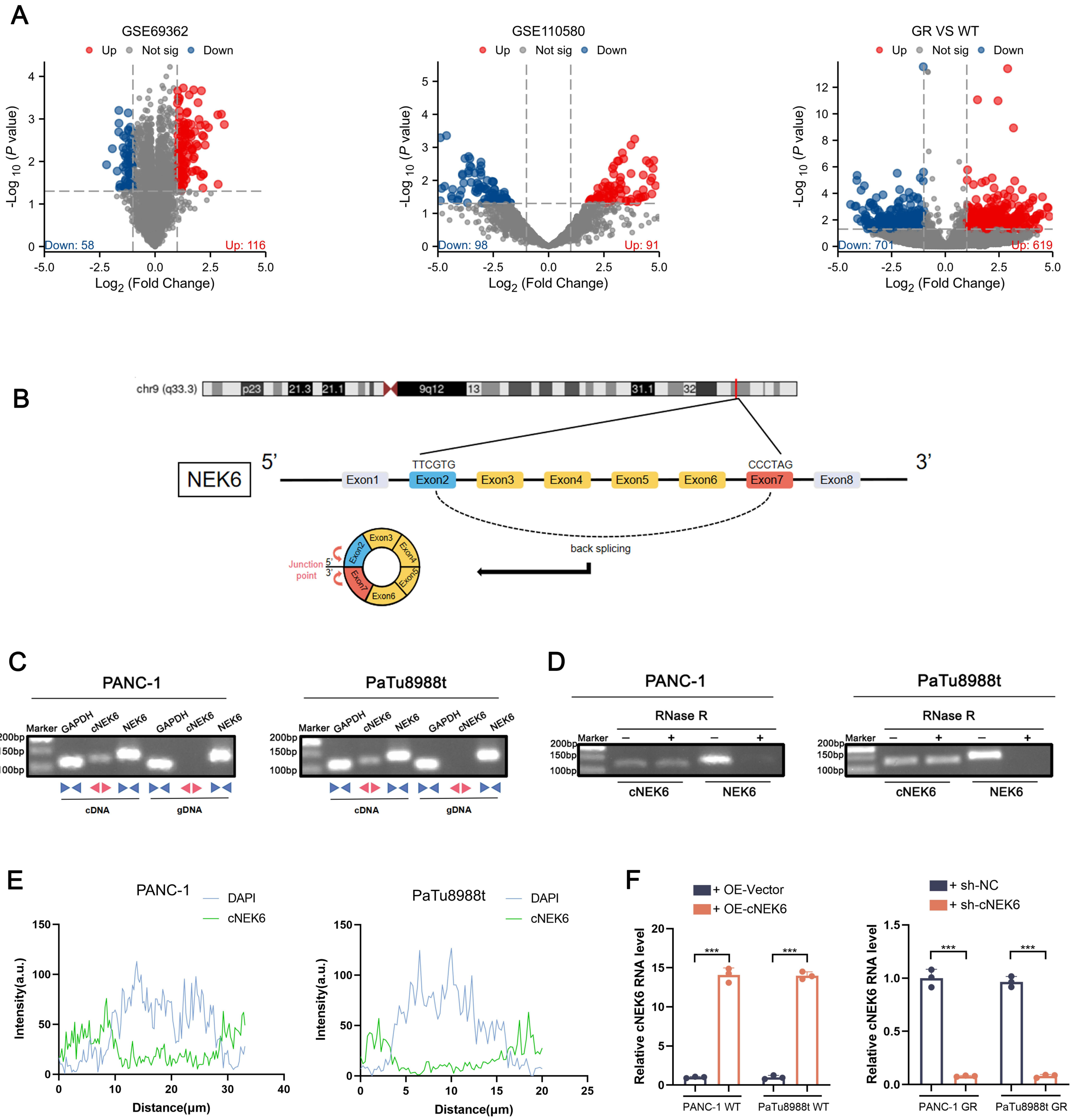
A
GSE69362
Up Not sig Down
-Log 10 (P value)
Log2 (Fold Change)
Down: 58
Up: 116
GSE110580
Down: 98
Up: 91
GR VS WT
Down: 701
Up: 619
B
chr9 (q33.3)
NEK6
5'
3'
TTCGTG
CCCTAG
Exon1
Exon2
Exon3
Exon4
Exon5
Exon6
Exon7
Exon8
back splicing
Junction point
C
PANC-1
PaTu8988t
Marker
GAPDH
cNEK6
NEK6
200bp
150bp
100bp
cDNA
gDNA
D
RNase R
E
DAPI
cNEK6
Intensity(a.u.)
Distance(μm)
F
+ OE-Vector
+ OE-cNEK6
Relative cNEK6 RNA level
PANC-1 WT
PaTu8988t WT
+ sh-NC
+ sh-cNEK6
PANC-1 GR
PaTu8988t GR

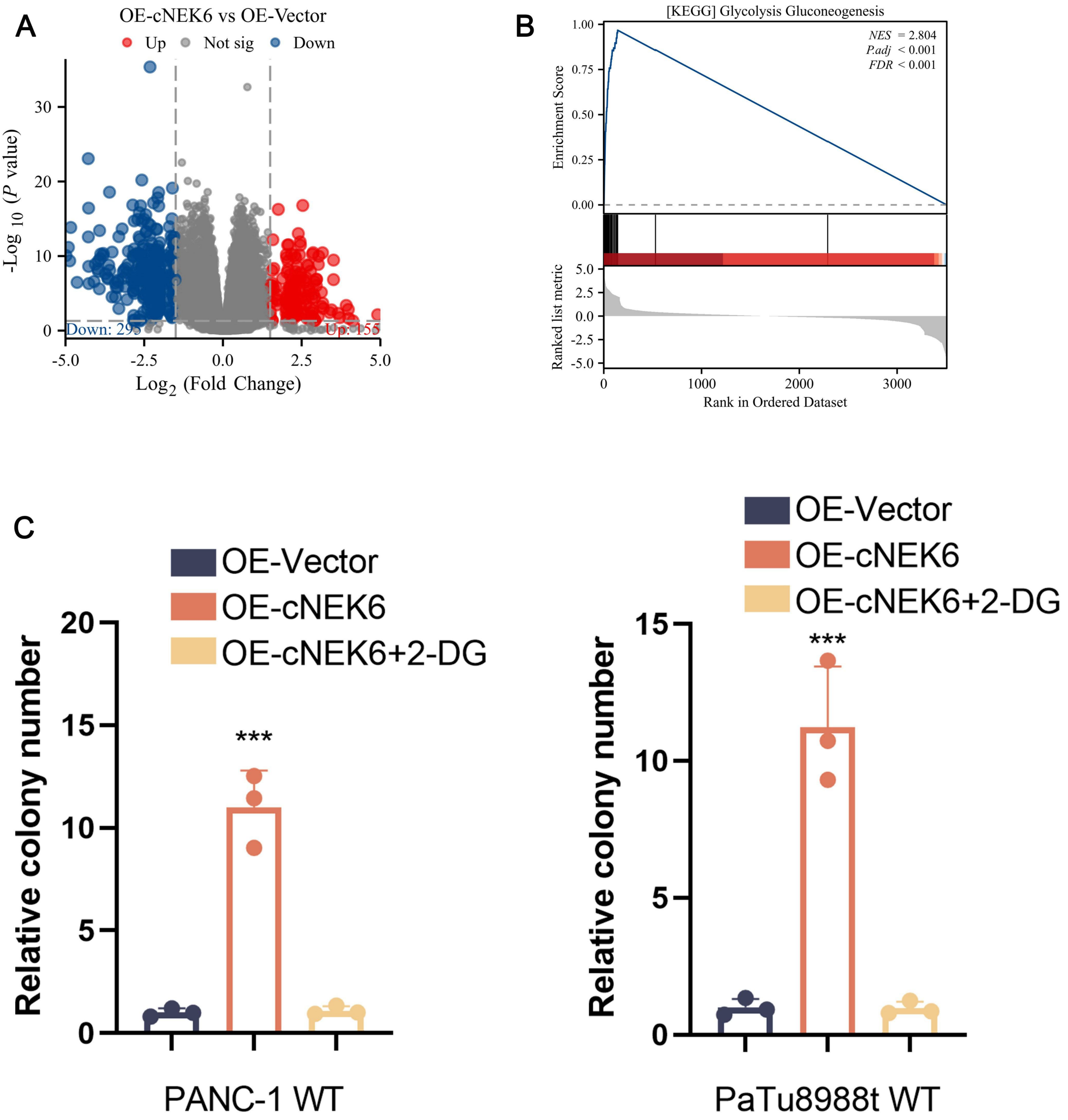
A
OE-cNEK6 vs OE-Vector
Up
Not sig
Down
-Log 10 (P value)
Log2 (Fold Change)
Down: 293
Up: 155
B
[KEGG] Glycolysis Gluconeogenesis
NES = 2.804
P.adj < 0.001
FDR < 0.001
Enrichment Score
Ranked list metric
Rank in Ordered Dataset
C
OE-Vector
OE-cNEK6
OE-cNEK6+2-DG
Relative colony number
***
PANC-1 WT
PaTu8988t WT

A

coding_potential_assessment

circBank ID: hsa_circNEK6_008

circRNA_size: 1953

ORF_size: 1923

Fickett_score: 1.3108

Hexamer_score: 0.4854

coding_prob: 0.9999

IRES elements

ID	start	end	score
No matching records found			

B

Sequence scan results

Your sequence:

```
1 UUCGUGCCCU CGUGAGGCUG GCAUGCAGGA UGGCAGGACA GCCCGGCCAC AUGCCCCAUG GAGGGAGUUC CAACAACCUC UGCCACACCC UGGGGCCUGU
101 GCAUCCUCCU GACCCACAGA GGCAUCCCAA CACGCUGUCU UUUCGCUGCU CGCUGGCGGA CUUCCAGAUC GAAAAGAAGA UAGGCCGAGG ACAGUUCAGC
201 GAGGUGUACA AGGCCACCUG CCUGCUGGAC AGGAAGACAG UGGCUCUGAA GAAGGUGCAG AUCUUUGAGA UGAUGGACGC CAAGGCGAGG CAGGACUGUG
301 UCAAGGAGAU CGGCCUCUUG AAGCAACUGA ACCACCCAAA UAUCAUCAAG UAUUUGGACU CGUUUAUCGA AGACAACGAG CUGAACAUUG UGCUGGAGUU
401 GGCUGACGCA GGGGACCUCU CGCAGAUGAU CAAGUACUUU AAGAAGCAGA AGCGGCUCAU CCCGGAGAGG ACAGUAUGGA AGUACUUUGU GCAGCUGUGC
501 AGCGCCGUGG AGCACAUGCA UUCACGCCGG GUGAUGCACC GAGACAUCAA GCCUGCCAAC GUGUUCAUCA CAGCCACGGG CGUCGUGAAG CUCGGUGACC
601 UUGGUCUGGG CCGCUUCUUC AGCUCUGAGA CCACCGCAGC CCACUCCCUA G
```

Score	Relative Score	RBP Name	Start	End	Matching sequence	Matrix ID	Download PWM	Download PFM
10.1470304	100%	SNRPA	304	310	AGGAGAU	949_10094314	Download PWM	Download PFM

C

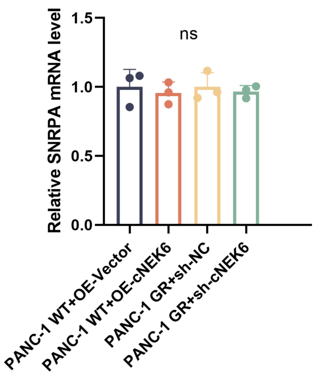

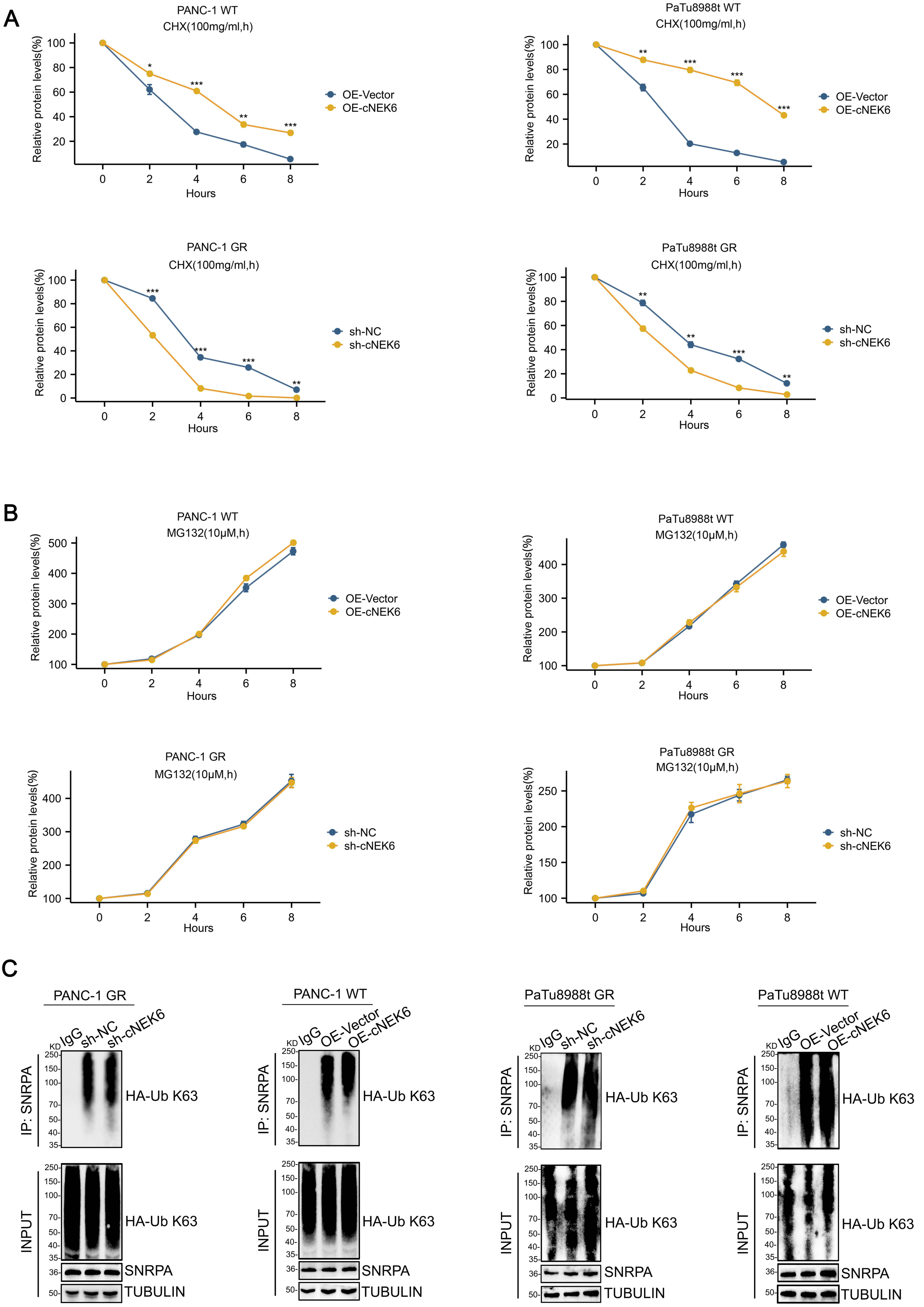

A
PANC-1 WT
CHX(100mg/ml,h)
Relative protein levels(%)
Hours
OE-Vector
OE-cNEK6
PaTu8988t WT
CHX(100mg/ml,h)
PANC-1 GR
CHX(100mg/ml,h)
sh-NC
sh-cNEK6
PaTu8988t GR
CHX(100mg/ml,h)
B
PANC-1 WT
MG132(10μM,h)
PaTu8988t WT
MG132(10μM,h)
PANC-1 GR
MG132(10μM,h)
PaTu8988t GR
MG132(10μM,h)
C
PANC-1 GR
IgG sh-NC sh-cNEK6
PANC-1 WT
IgG OE-Vector OE-cNEK6
PaTu8988t GR
IgG sh-NC sh-cNEK6
PaTu8988t WT
IgG OE-Vector OE-cNEK6
KD
IP: SNRPA
INPUT
HA-Ub K63
SNRPA
TUBULIN

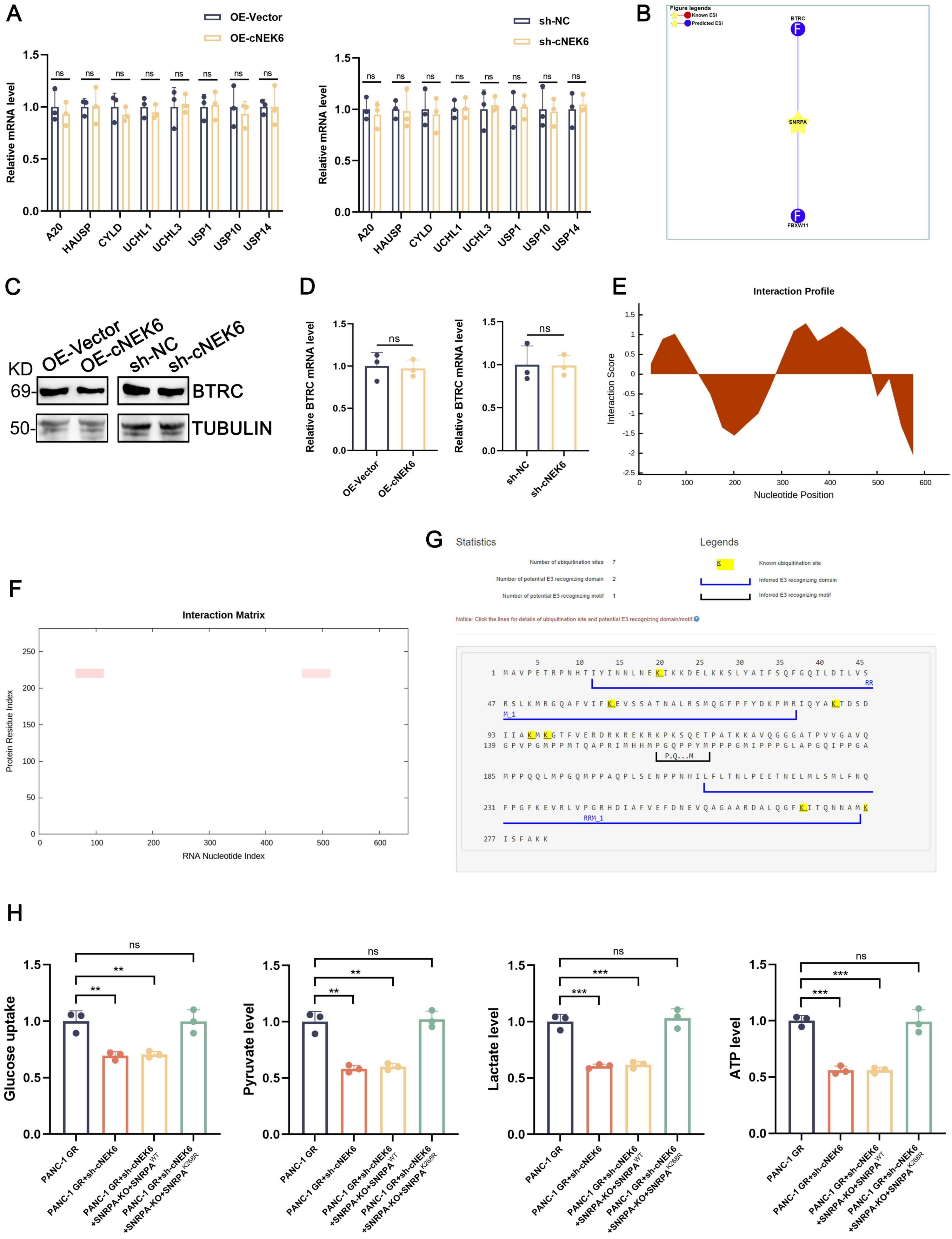
A
OE-Vector
OE-cNEK6
Relative mRNA level
A20
HAUSP
CYLD
UCHL1
UCHL3
USP1
USP10
USP14
ns
sh-NC
sh-cNEK6
B
Figure legends
Known ESI
Predicted ESI
BTRC
SNRPA
FBXW11
C
KD
OE-Vector
OE-cNEK6
sh-NC
sh-cNEK6
69-
50-
BTRC
TUBULIN
D
Relative BTRC mRNA level
E
Interaction Profile
Interaction Score
Nucleotide Position
F
Interaction Matrix
Protein Residue Index
RNA Nucleotide Index
G
Statistics
Legends
Number of ubiquitination sites 7
Known ubiquitination site
Number of potential E3 recognizing domain 2
Inferred E3 recognizing domain
Number of potential E3 recoginzing motif 1
Inferred E3 recognizing motif
Notice: Click the lines for details of ubiquitination site and potential E3 recognizing domain/motif.
1 M A V P E T R P N H T I Y I N N L N E K I K K D E L K K S L Y A I F S Q F G Q I L D I L V S
RR
47 R S L K M R G Q A F V I F K E V S S A T N A L R S M Q G F P F Y D K P M R I Q Y A K T D S D
M_1
93 I I A K M K G T F V E R D R K R E K R K P K S Q E T P A T K K A V Q G G G A T P V V G A V Q
139 G P V P G M P P M T Q A P R I M H H M P G Q P P Y M P P P G M I P P P G L A P G Q I P P G A
P.Q...M
185 M P P Q Q L M P G Q M P P A Q P L S E N P P N H I L F L T N L P E E T N E L M L S M L F N Q
231 F P G F K E V R L V P G R H D I A F V E F D N E V Q A G A A R D A L Q G F K I T Q N N A M K
RRM_1
277 I S F A K K
H
Glucose uptake
Pyruvate level
Lactate level
ATP level
PANC-1 GR
PANC-1 GR+sh-cNEK6
PANC-1 GR+sh-cNEK6 +SNRPA-KO+SNRPA WT
PANC-1 GR+sh-cNEK6 +SNRPA-KO+SNRPA K268R
ns
**
***

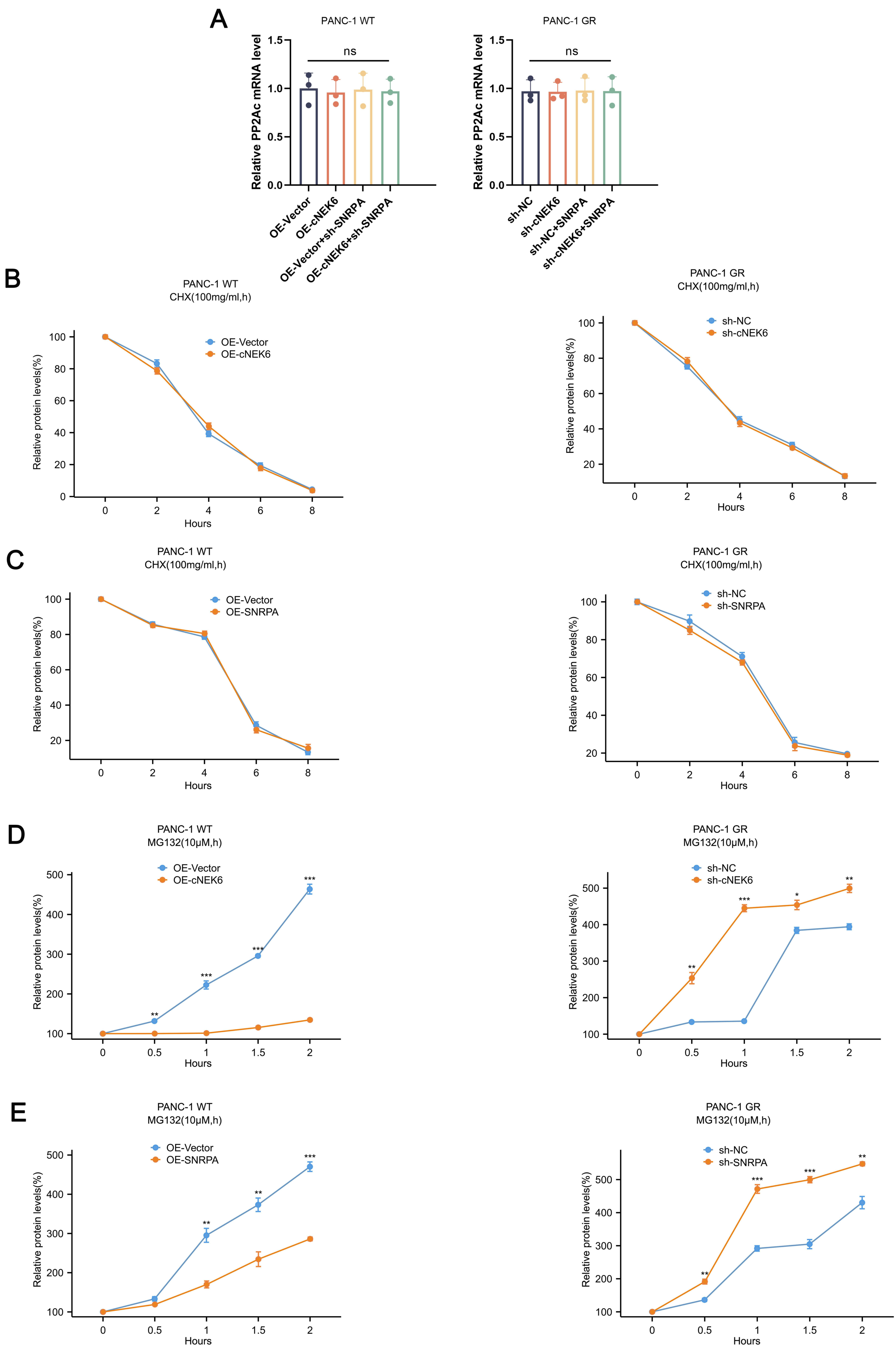
A
PANC-1 WT
Relative PP2Ac mRNA level
1.5
1.0
0.5
0.0
ns
OE-Vector
OE-cNEK6
OE-Vector+sh-SNRPA
OE-cNEK6+sh-SNRPA
PANC-1 GR
Relative PP2Ac mRNA level
ns
sh-NC
sh-cNEK6
sh-NC+SNRPA
sh-cNEK6+SNRPA
B
PANC-1 WT
CHX(100mg/ml,h)
Relative protein levels(%)
OE-Vector
OE-cNEK6
Hours
PANC-1 GR
CHX(100mg/ml,h)
Relative protein levels(%)
sh-NC
sh-cNEK6
Hours
C
PANC-1 WT
CHX(100mg/ml,h)
Relative protein levels(%)
OE-Vector
OE-SNRPA
Hours
PANC-1 GR
CHX(100mg/ml,h)
Relative protein levels(%)
sh-NC
sh-SNRPA
Hours
D
PANC-1 WT
MG132(10μM,h)
Relative protein levels(%)
OE-Vector
OE-cNEK6
Hours
PANC-1 GR
MG132(10μM,h)
Relative protein levels(%)
sh-NC
sh-cNEK6
Hours
E
PANC-1 WT
MG132(10μM,h)
Relative protein levels(%)
OE-Vector
OE-SNRPA
Hours
PANC-1 GR
MG132(10μM,h)
Relative protein levels(%)
sh-NC
sh-SNRPA
Hours

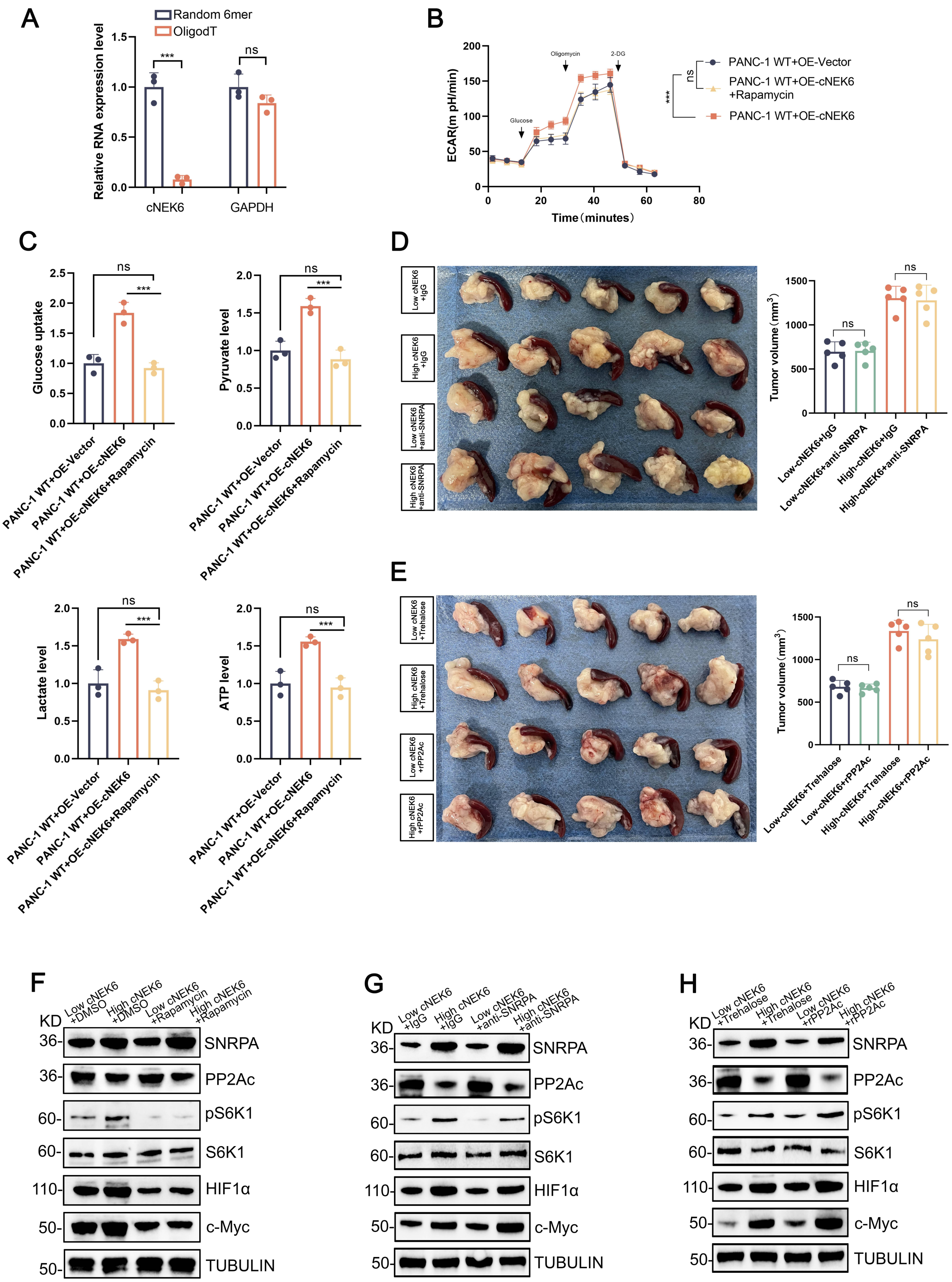
A
Random 6mer
OligodT
Relative RNA expression level
***
ns
cNEK6
GAPDH
B
ECAR(m pH/min)
Glucose
Oligomycin
2-DG
Time（minutes）
ns
***
PANC-1 WT+OE-Vector
PANC-1 WT+OE-cNEK6 +Rapamycin
PANC-1 WT+OE-cNEK6
C
Glucose uptake
Pyruvate level
Lactate level
ATP level
PANC-1 WT+OE-Vector
PANC-1 WT+OE-cNEK6
PANC-1 WT+OE-cNEK6+Rapamycin
D
Low cNEK6 +IgG
High cNEK6 +IgG
Low cNEK6 +anti-SNRPA
High cNEK6 +anti-SNRPA
Tumor volume (mm3)
Low-cNEK6+IgG
Low-cNEK6+anti-SNRPA
High-cNEK6+IgG
High-cNEK6+anti-SNRPA
E
Low cNEK6 +Trehalose
High cNEK6 +Trehalose
Low cNEK6 +rPP2Ac
High cNEK6 +rPP2Ac
Low-cNEK6+Trehalose
Low-cNEK6+rPP2Ac
High-cNEK6+Trehalose
High-cNEK6+rPP2Ac
F
Low cNEK6 +DMSO
High cNEK6 +DMSO
Low cNEK6 +Rapamycin
High cNEK6 +Rapamycin
G
Low cNEK6 +IgG
High cNEK6 +IgG
Low cNEK6 +anti-SNRPA
High cNEK6 +anti-SNRPA
H
Low cNEK6 +Trehalose
High cNEK6 +Trehalose
Low cNEK6 +rPP2Ac
High cNEK6 +rPP2Ac
KD
SNRPA
PP2Ac
pS6K1
S6K1
HIF1α
c-Myc
TUBULIN
