# Supplementary material for: cNEK6 induces gemcitabine resistance by promoting glycolysis in pancreatic ductal adenocarcinoma via the SNRPA/PPA2c/mTORC1 axis
Source: Cell Death Dis. 2024 Oct 11;15(10):742. doi: 10.1038/s41419-024-07138-y (PMC11470042; doi:10.1038/s41419-024-07138-y)
Supplement: Supplementary file 2 — Original Western Blot [file 41419_2024_7138_MOESM2_ESM.pdf]

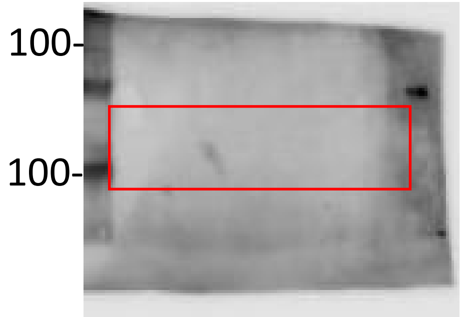

(FIg. 3C AGO2)

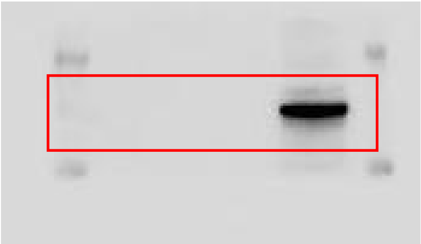
(FIg. 3C SNRPA)

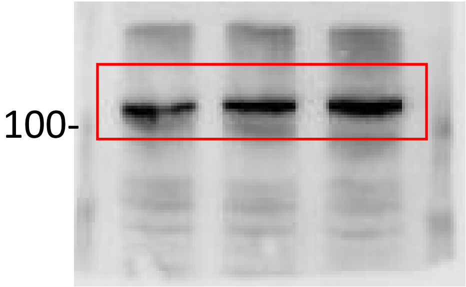

(FIg. 3C AGO2)

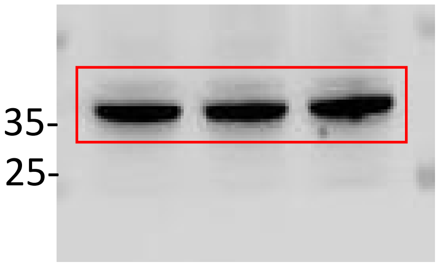

(FIg. 3C SNRPA)

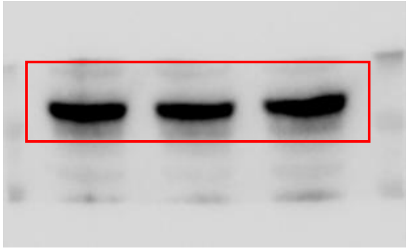
(FIg. 3C TUBULIN)

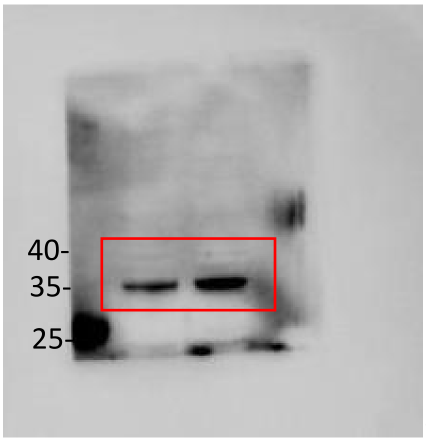

(FIg. 3F SNRPA)

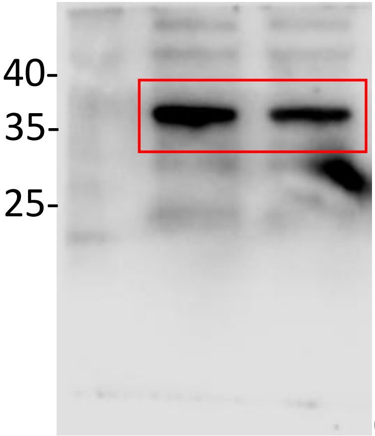

(FIg. 3F SNRPA)

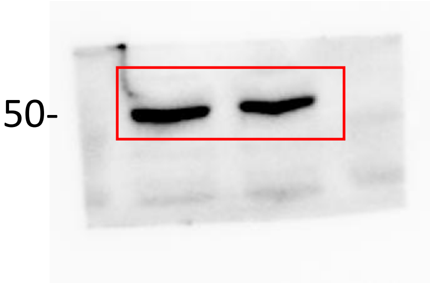

(FIg. 3F TUBULIN)

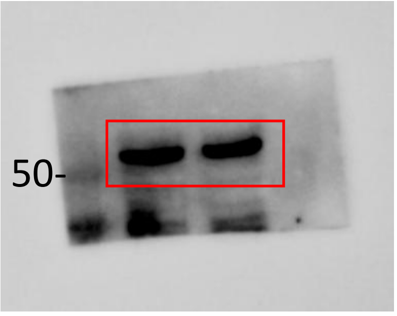

(FIg. 3F TUBULIN)

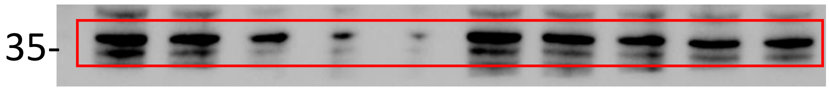

(FIg. 4A SNRPA PANC-1 WT)

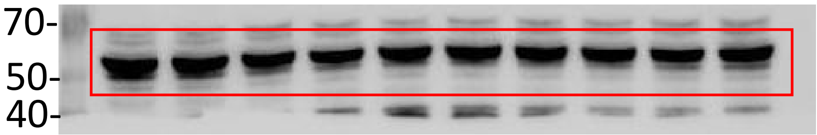

(FIg. 4A TUBULIN PANC-1 WT)

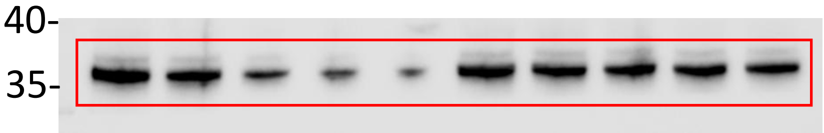

(FIg. 4A SNRPA 8988 WT)

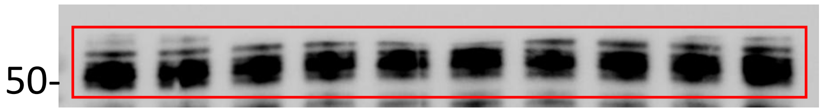

(FIg. 4A TUBULIN 8988 WT)

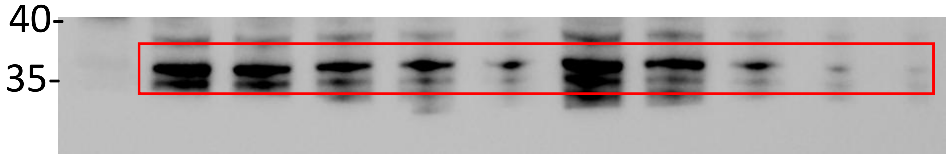

(FIg. 4A SNRPA PANC-1 GR)

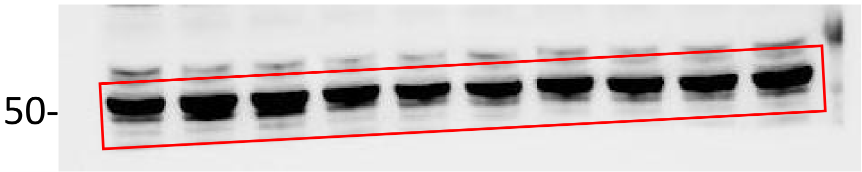

(FIg. 4A TUBULIN PANC-1 GR)

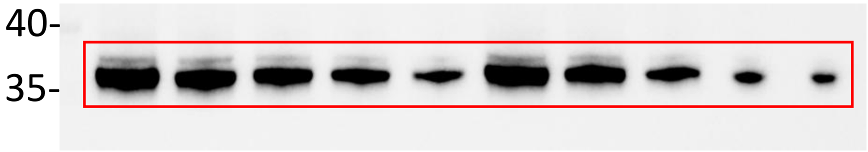

(FIg. 4A SNRPA 8988 GR)

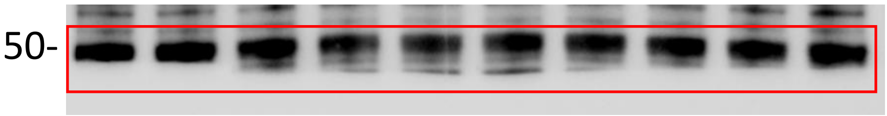

(FIg. 4A TUBULIN 8988 GR)

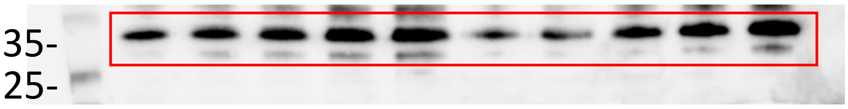

(FIg. 4B SNRPA PANC-1 WT)

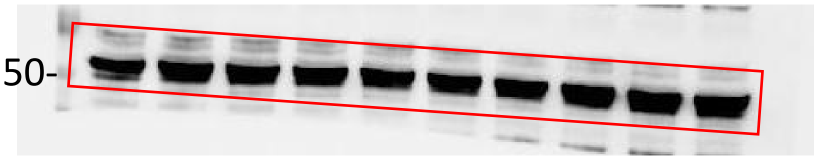

(FIg. 4B TUBULIN PANC-1 WT)

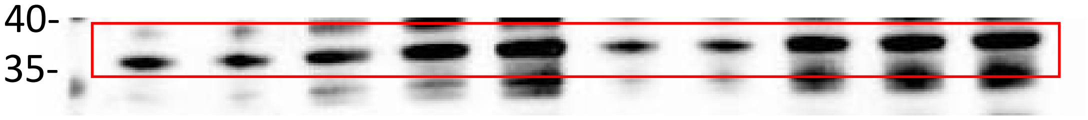

(FIg. 4B SNRPA 8988 WT)

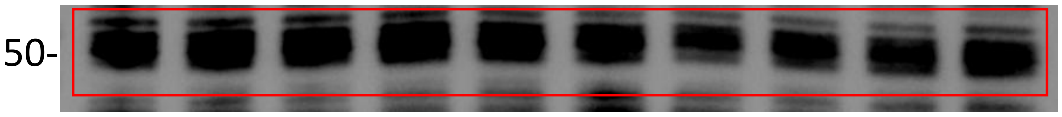

(FIg. 4B TUBULIN 8988 WT)

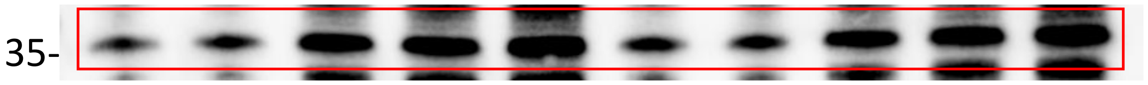

(FIg. 4B SNRPA PANC-1 GR)

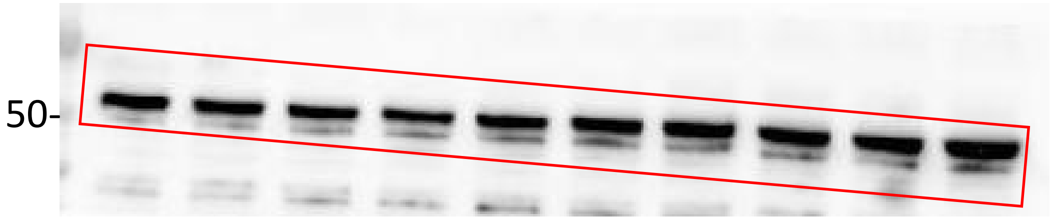

(FIg. 4B TUBULIN PANC-1 GR)

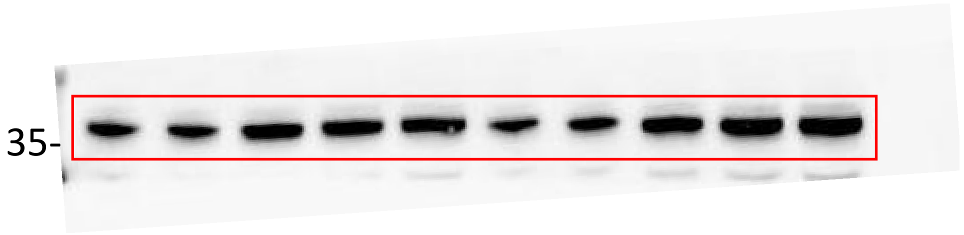

(FIg. 4B SNRPA 8988 GR sh-NC)

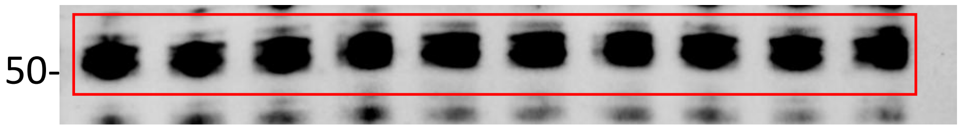

(FIg. 4B TUBULIN 8988 GR)

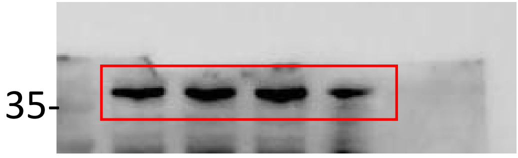

(FIg. 4C SNRPA PANC-1 GR)

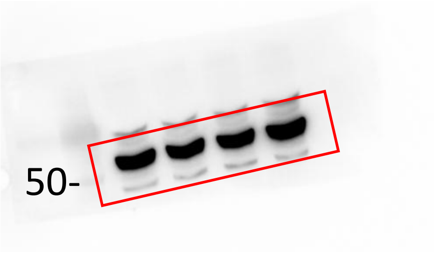

(FIg. 4C TUBULIN PANC-1 GR)

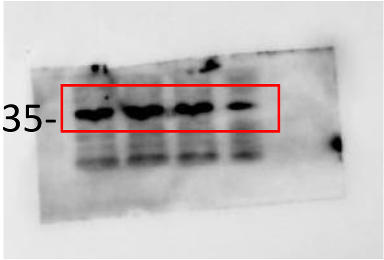

(FIg. 4C SNRPA 8988 GR)

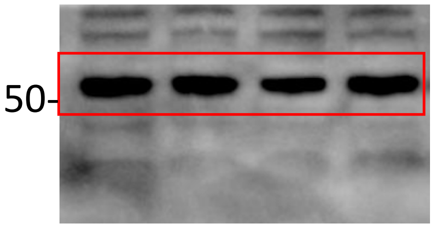

(FIg. 4C TUBULIN 8988 GR)

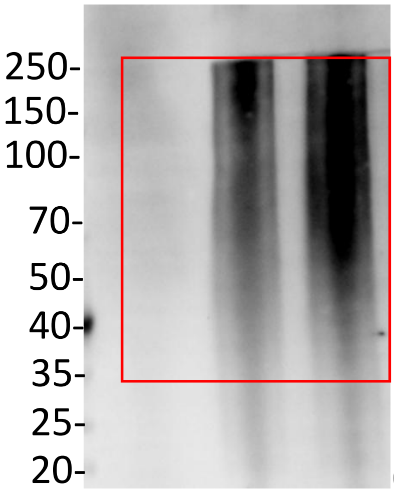

(FIg. 4D HA-Ub PANC-1 GR IP)

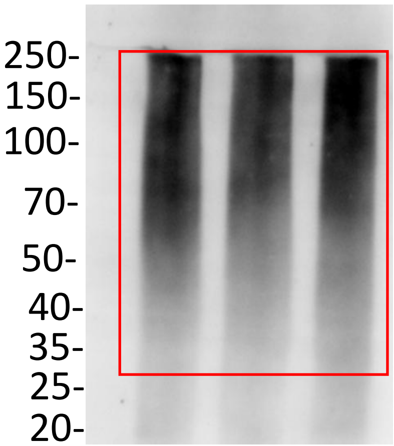

(FIg. 4D HA-Ub PANC-1 GR INPUT)

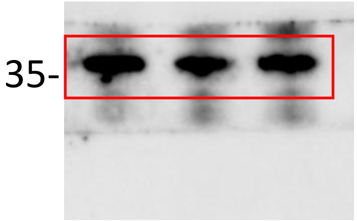

(FIg. 4D SNRPA PANC-1 GR INPUT)

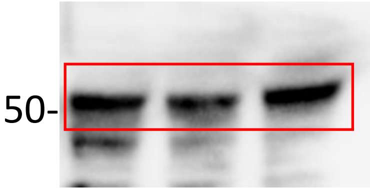

(FIg. 4D TUBULIN PANC-1 GR INPUT)

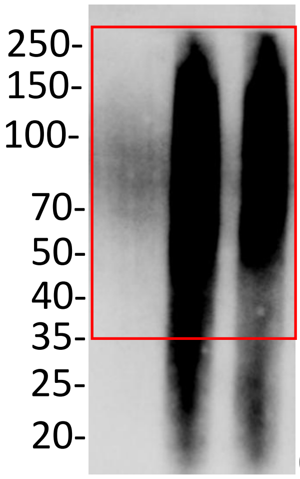

(FIg. 4D HA-Ub PANC-1 WT IP)

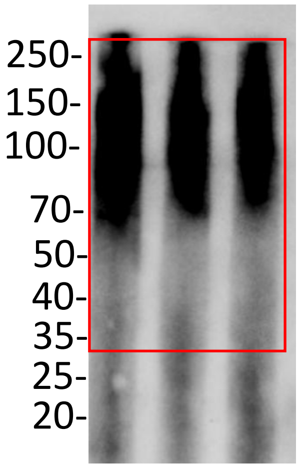

(FIg. 4D HA-Ub PANC-1 WT INPUT)

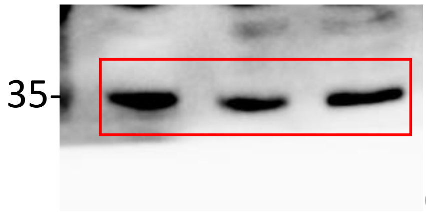

(FIg. 4D SNRPA PANC-1 WT INPUT)

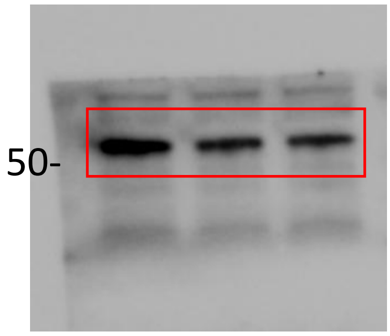

(FIg. 4D TUBULIN PANC-1 WT INPUT)

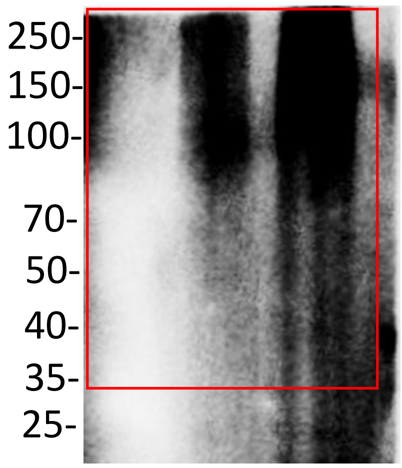

(FIg. 4D HA-Ub 8988 GR IP)

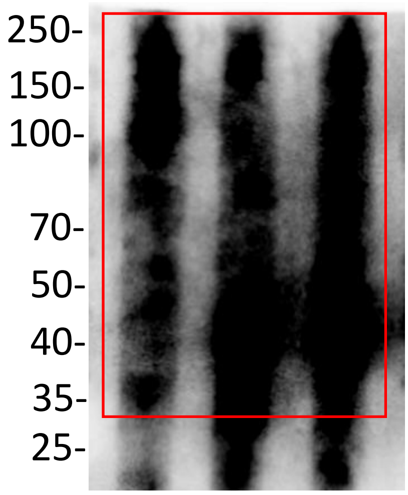

(FIg. 4D HA-Ub 8988 GR INPUT)

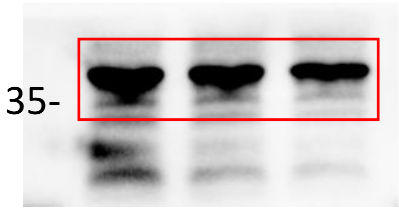

(FIg. 4D SNRPA 8988 GR INPUT)

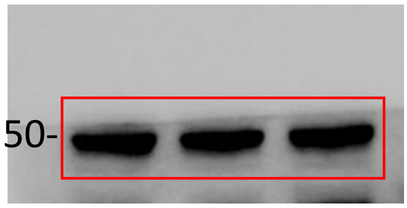

(FIg. 4D TUBULIN 8988 GR INPUT)

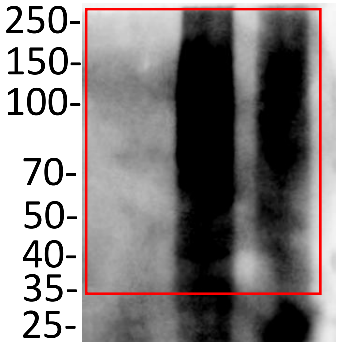

(FIg. 4D HA-Ub 8988 WT IP)

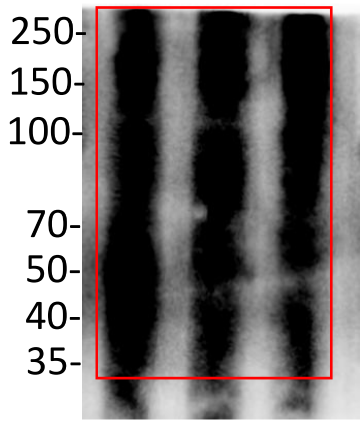

(FIg. 4D HA-Ub 8988 WT INPUT)

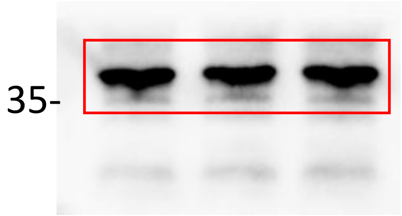

(FIg. 4D SNRPA 8988 WT INPUT)

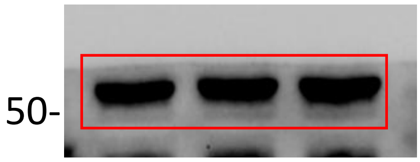

(FIg. 4D TUBULIN 8988 WT INPUT)

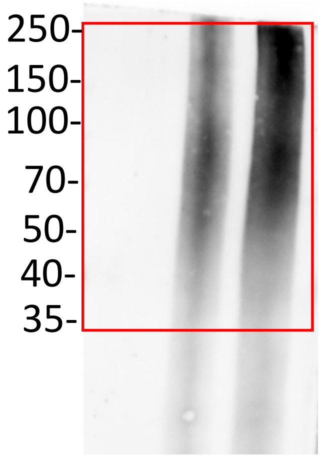

(FIg. 4E HA-Ub K48 PANC-1 GR IP)

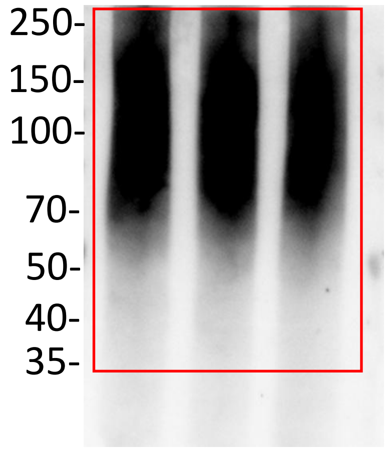

(FIg. 4E HA-Ub K48 PANC-1 GR INPUT)

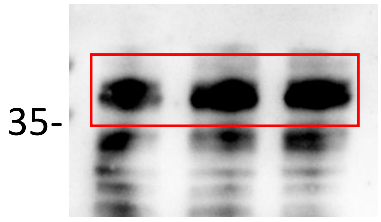

(FIg. 4E SNRPA PANC-1 GR INPUT)

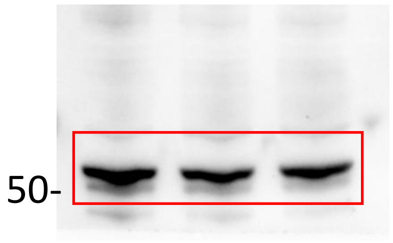

(FIg. 4E TUBULIN PANC-1 GR INPUT)

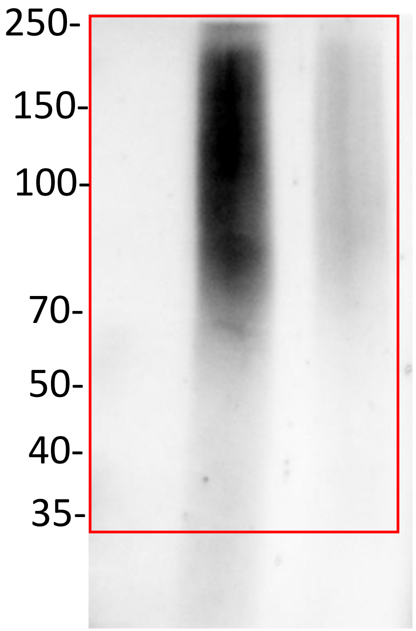

(FIg. 4E HA-Ub K48 PANC-1 WT IP)

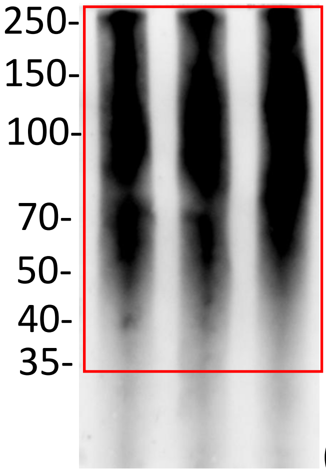

(FIg. 4E HA-Ub K48 PANC-1 WT INPUT)

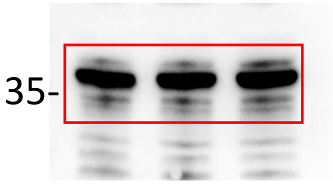

(FIg. 4E SNRPA PANC-1 WT INPUT)

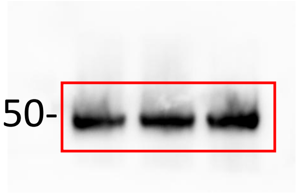

(FIg. 4E TUBULN PANC-1 WT INPUT)

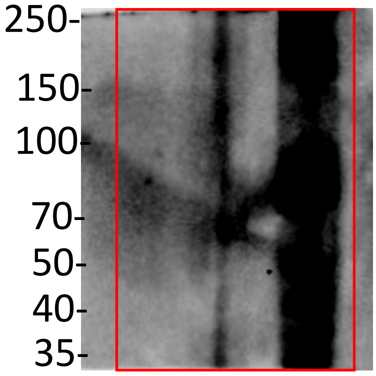

(FIg. 4E HA-Ub K48 8988 GR IP)

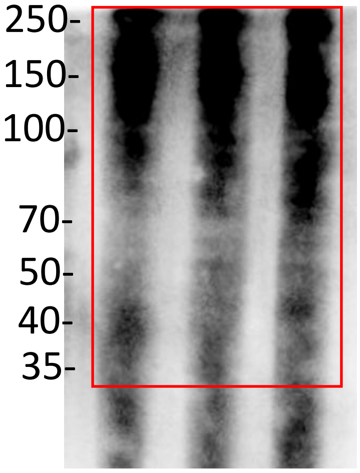

(FIg. 4E HA-Ub K48 8988 GR INPUT)

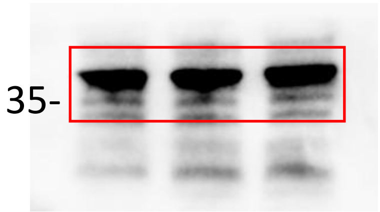

(FIg. 4E SNRPA 8988 GR INPUT)

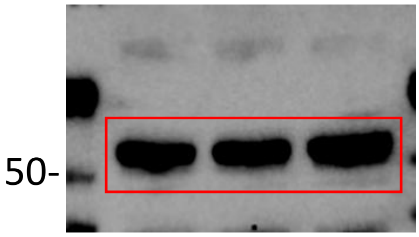

(FIg. 4E TUBULIN 8988 GR INPUT)

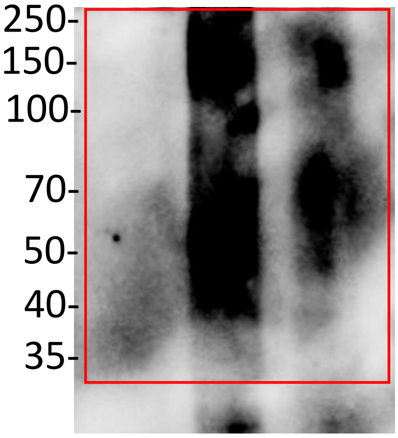

(FIg. 4E HA-Ub K48 8988 WT IP)

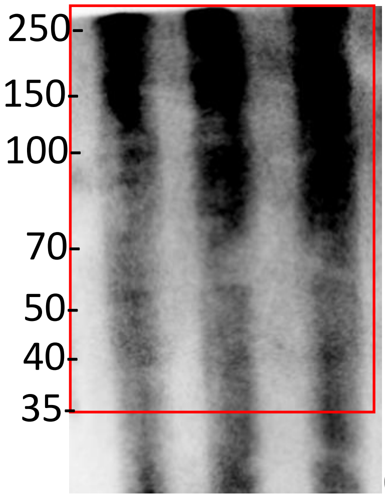

(FIg. 4E HA-Ub K48 8988 WT INPUT)

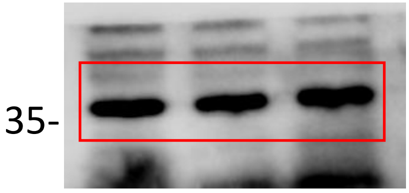

(FIg. 4E SNRPA 8988 WT INPUT)

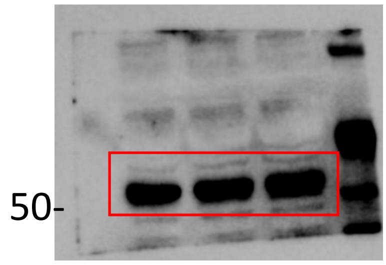

(FIg. 4E TUBULIN 8988 WT INPUT)

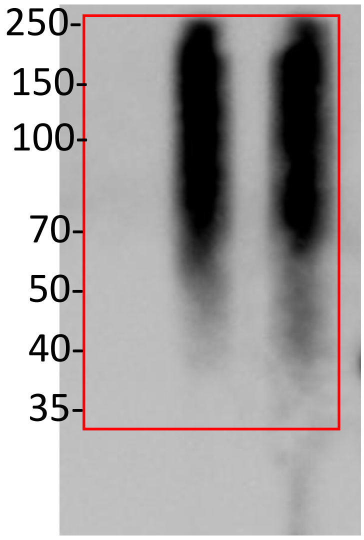

(FIg. S4C HA-Ub K63 PANC-1 GR IP)

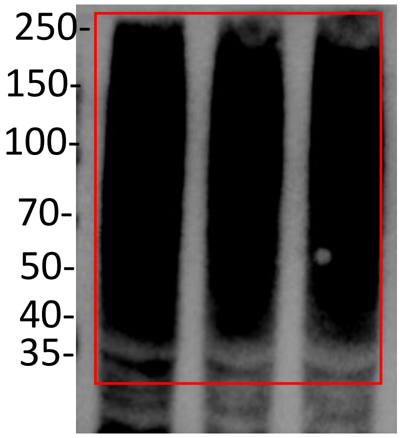

(FIg. S4C HA-Ub K63 PANC-1 GR INPUT)

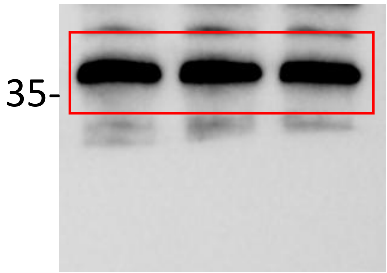

(FIg. S4C SNRPA PANC-1 GR INPUT)

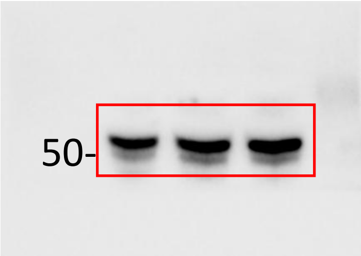

(FIg. S4C TUBULIN PANC-1 GR INPUT)

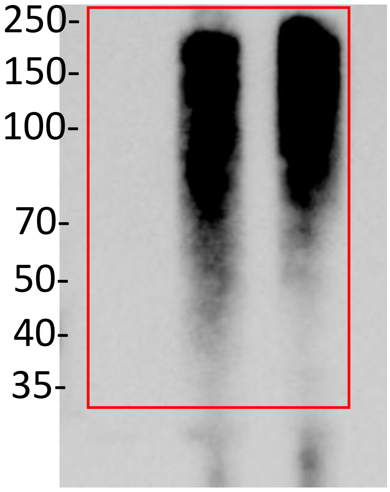

(FIg. S4C HA-Ub K63 PANC-1 WT IP)

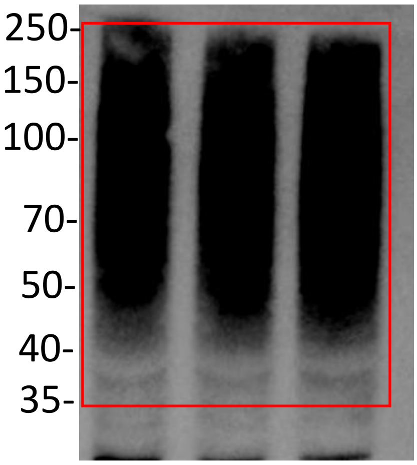

(FIg. S4C HA-Ub K63 PANC-1 WT INPUT)

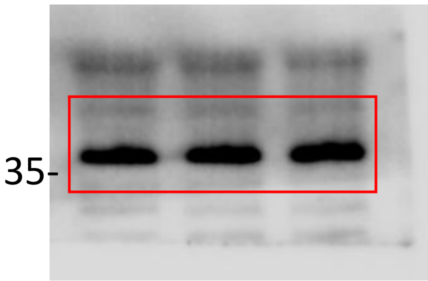

(FIg. S4C SNRPA PANC-1 WT INPUT)

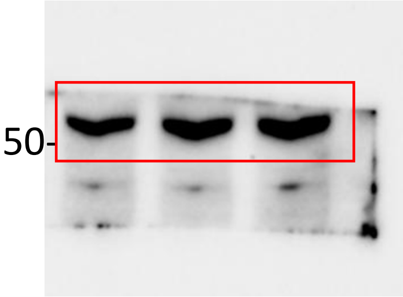

(FIg. S4C TUBULIN PANC-1 WT INPUT)

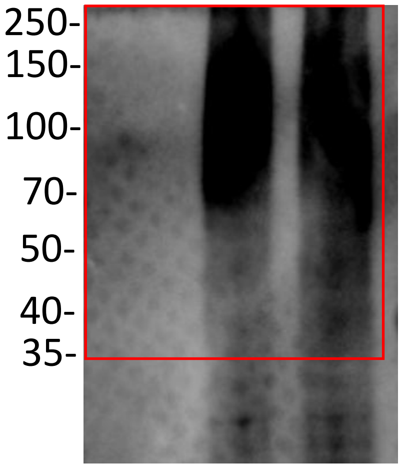

(FIg. S4C HA-Ub K63 8988 GR IP)

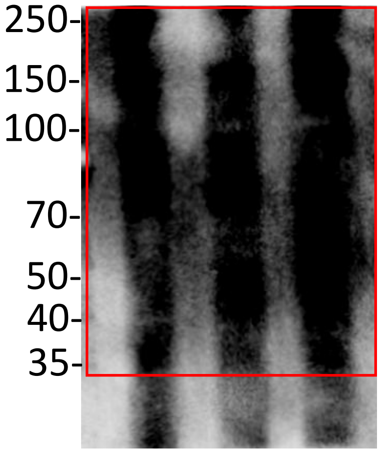

(FIg. S4C HA-Ub K63 8988 GR INPUT)

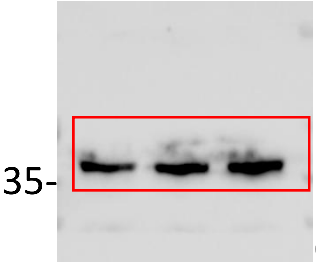

(FIg. S4C SNRPA 8988 GR INPUT)

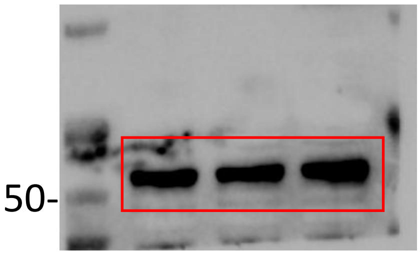

(FIg. S4C TUBULIN 8988 GR INPUT)

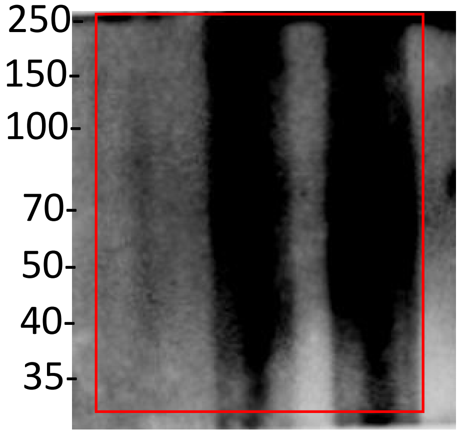

(FIg. S4C HA-Ub K63 8988 WT IP)

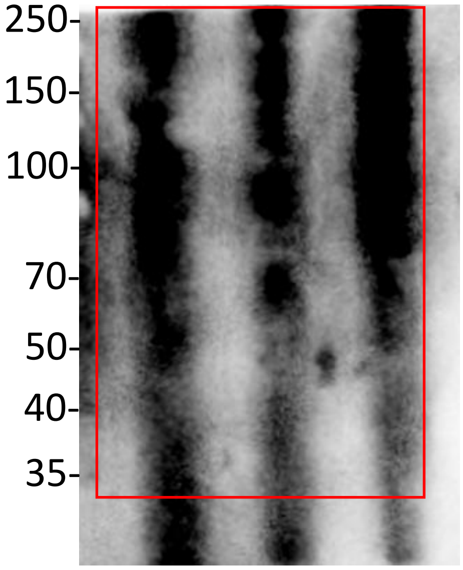

(FIg. S4C HA-Ub K63 8988 WT INPUT)

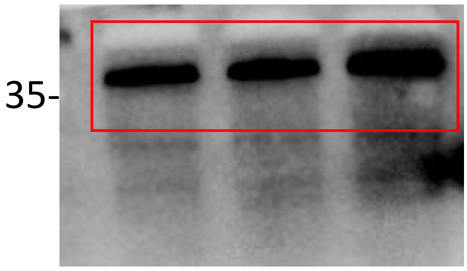

(FIg. S4C SNRPA 8988 WT INPUT)

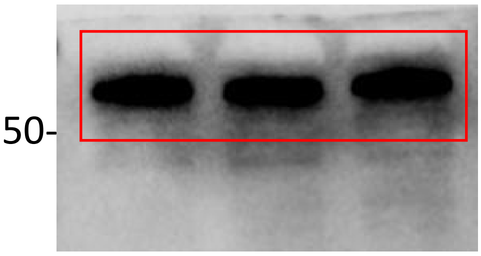

(FIg. S4C TUBULIN 8988 WT INPUT)

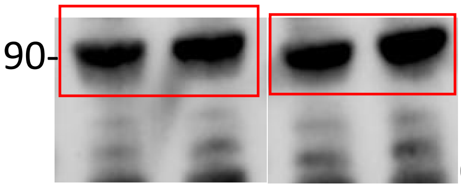

(FIg. 5A A20)

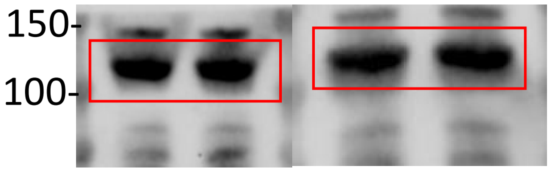

(FIg. 5A HAUSP)

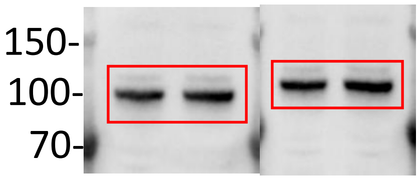

(FIg. 5A p-CYLD)

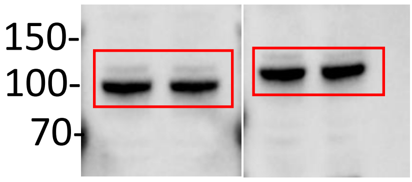

(FIg. 5A CYLD)

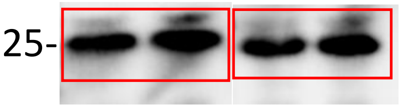

(FIg. 5A UCHL1)

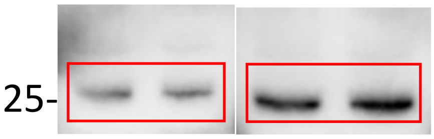

(FIg. 5A UCHL3)

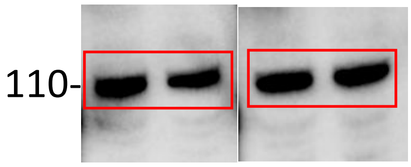

(FIg. 5A USP1)

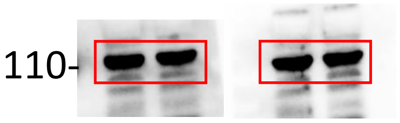

(FIg. 5A USP10)

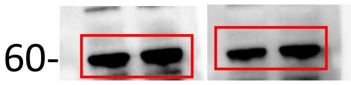

(FIg. 5A USP14)

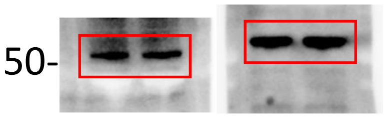

(FIg. 5A TUBULIN)

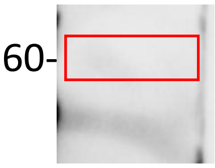

(FIg. 5B FBXW11 IP)

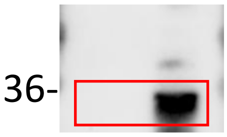

(FIg. 5B SNRPA IP)

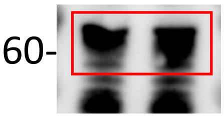

(FIg. 5B FBXW11 INPUT)

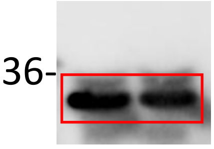

(FIg. 5B SNRPA INPUT)

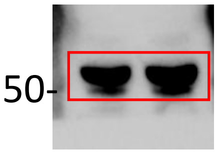

(FIg. 5B TUBULIN INPUT)

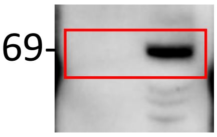

(FIg. 5B BTRC IP)

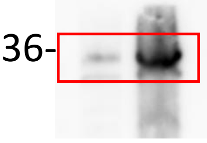

(FIg. 5B SNRPA IP)

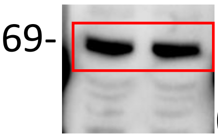

(FIg. 5B BTRC INPUT)

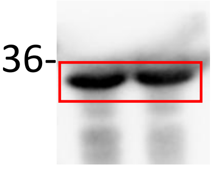

(FIg. 5B SNRPA INPUT)

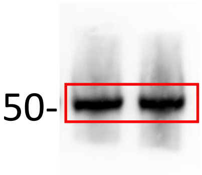

(FIg. 5B TUBULIN INPUT)

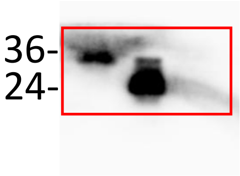

(FIg. 5C FLAG-SNRPA IP)

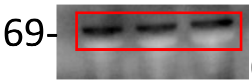

(FIg. 5C MYC-BTRC IP)

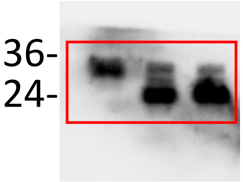

(FIg. 5C FLAG-SNRPA INPUT)

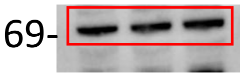

(FIg. 5C MYC-BTRC INPUT)

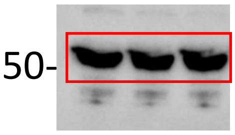

(FIg. 5C TUBULIN INPUT)

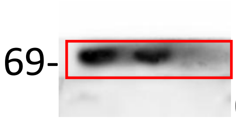

(FIg. 5C MYC-BTRC IP)

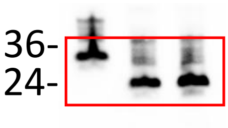

(FIg. 5C FLAG-SNRPA IP)

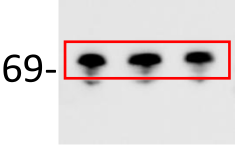

(FIg. 5C MYC-BTRC INPUT)

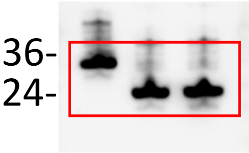

(FIg. 5C FLAG-SNRPA INPUT)

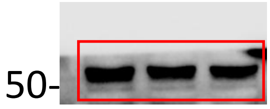

(FIg. 5C TUBULIN INPUT)

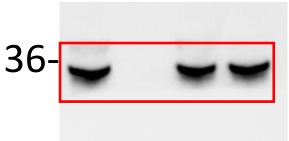

(FIg. 5D FLAG IP)

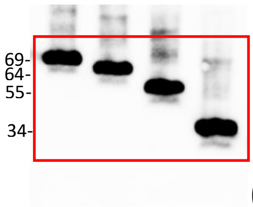

(FIg. 5D MYC IP)

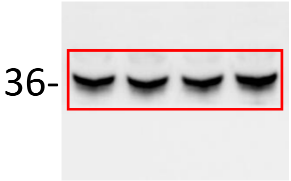

(FIg. 5D FLAG INPUT)

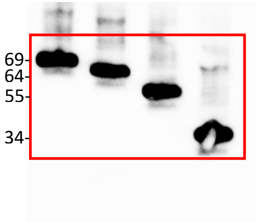

(FIg. 5D MYC INPUT)

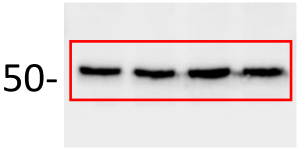

(FIg. 5D TUBULIN INPUT)

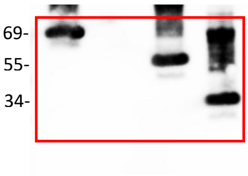

(FIg. 5D MYC IP)

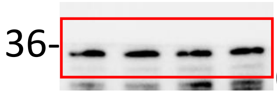

(FIg. 5D FLAG IP)

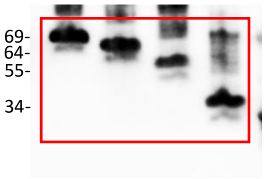

(FIg. 5D MYC INPUT)

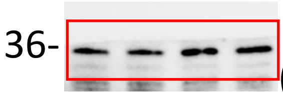

(FIg. 5D FLAG INPUT)

50-
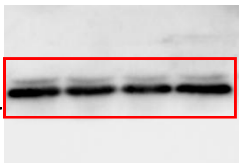
(FIg. 5D TUBULIN INPUT)

36-
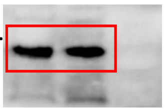
(FIg. 5E SNRPA PULLDOWN)

36-
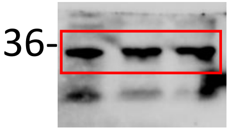
(FIg. 5E SNRPA INPUT)

36-
24-
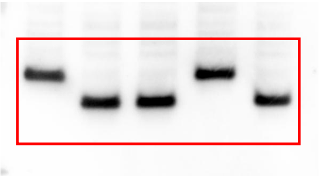
(FIg. 5F FLAG)

69-
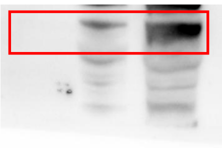
(FIg. 5G BTRC IP)

36-
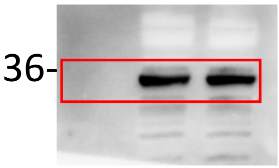
(FIg. 5G SNRPA IP)

69-
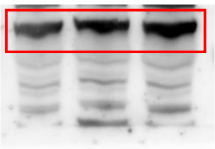
(FIg. 5G BTRC INPUT)

36-
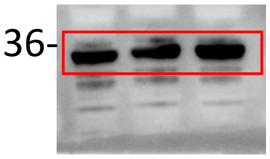
(FIg. 5G SNRPA INPUT)

50-
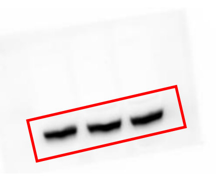
(FIg. 5G TUBULIN INPUT)

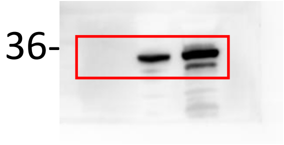

(FIg. 5G SNRPA IP)

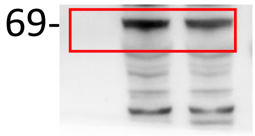

(FIg. 5G BTRC IP)

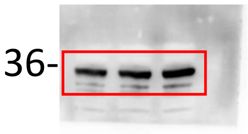

(FIg. 5G SNRPA INPUT)

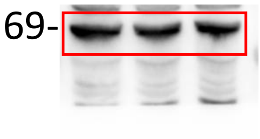

(FIg. 5G BTRC INPUT)

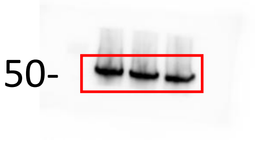

(FIg. 5G TUBULIN INPUT)

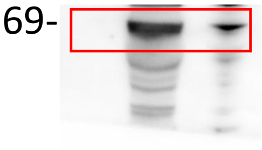

(FIg. 5G BTRC IP)

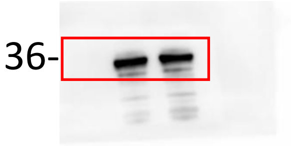

(FIg. 5G SNRPA IP)

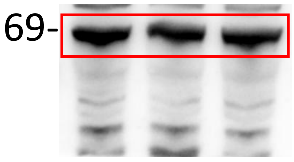

(FIg. 5G BTRC INPUT)

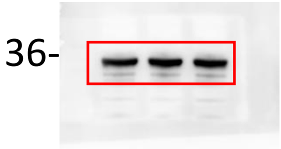

(FIg. 5G SNRPA INPUT)

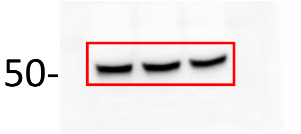

(FIg. 5G TUBULIN INPUT)

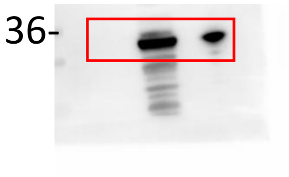

(FIg. 5G SNRPA IP)

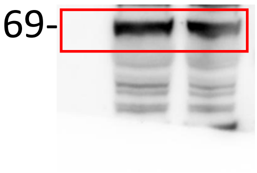

(FIg. 5G BTRC IP)

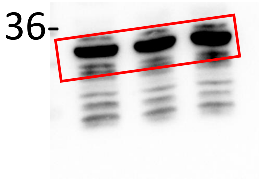

(FIg. 5G SNRPA INPUT)

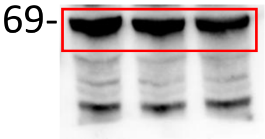

(FIg. 5G BTRC INPUT)

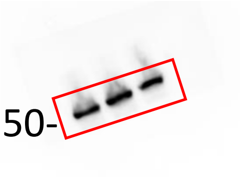

(FIg. 5G TUBULIN INPUT)

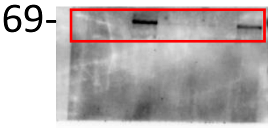

(FIg. 5H BTRC IP)

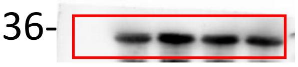

(FIg. 5H SNRPA IP)

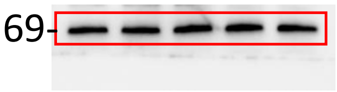

(FIg. 5H BTRC INPUT)

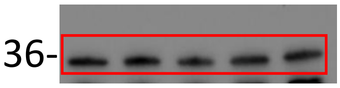

(FIg. 5H SNRPA INPUT)

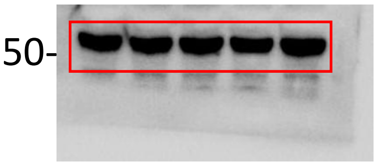

(FIg. 5H TUBULIN)

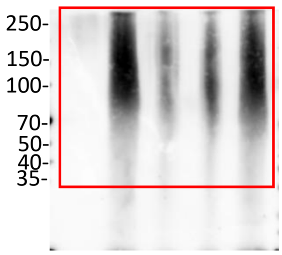

(FIg. 5I UB-K48 IP)

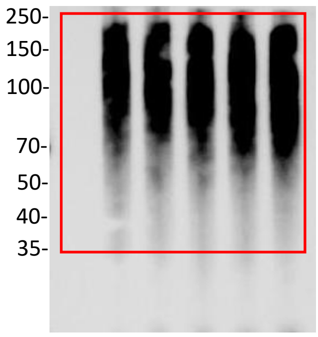

(FIg. 5I UB-K48 INPUT)

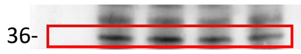

(FIg. 5I SNRPA INPUT)

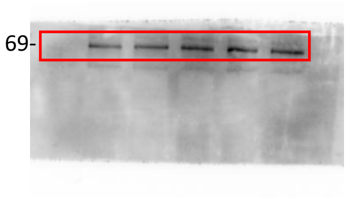

(FIg. 5I BTRC INPUT)

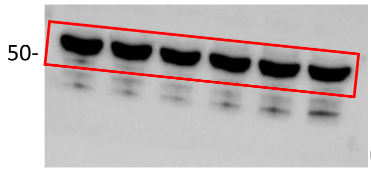

(FIg. 5I TUBULIN)

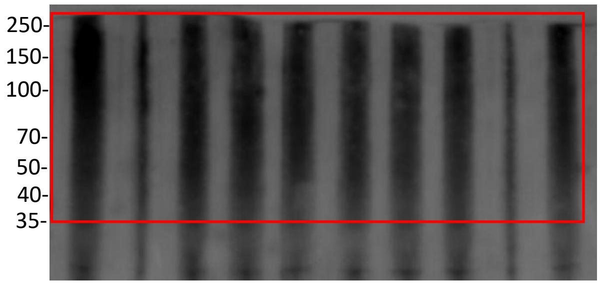

(FIg. 5J UB-K48 IP)

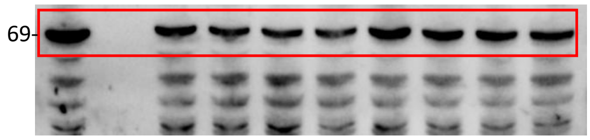

(FIg. 5J MYC-BTRC INPUT)

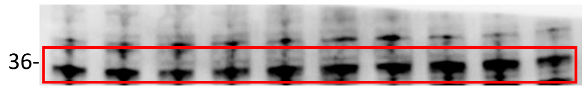

(FIg. 5J FLAG-SNRPA INPUT)

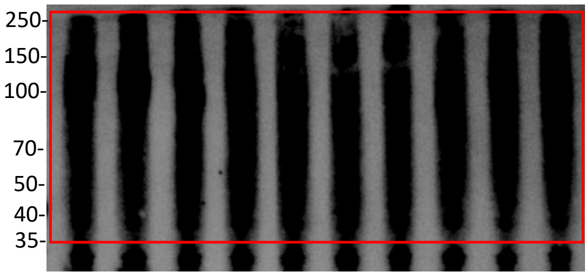

(FIg. 5J UB-K48 INPUT)

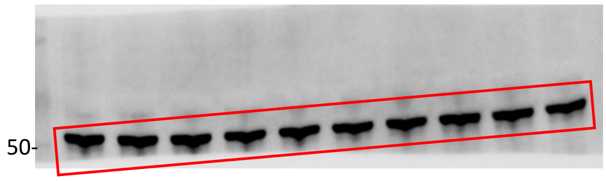

(FIg. 5J TUBULIN)

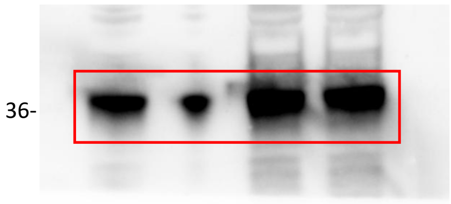

(FIg. 6B PP2Ac)

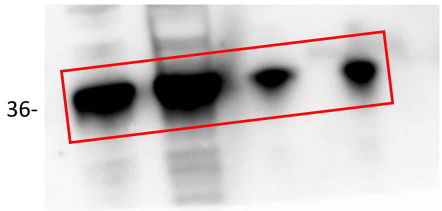

(FIg. 6B PP2Ac)

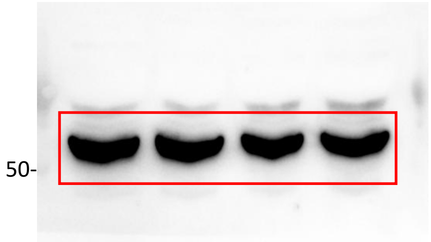

(FIg. 6B TUBULIN)

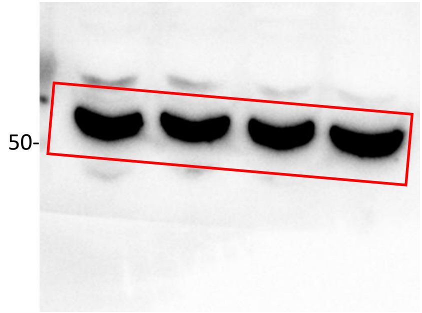

(FIg. 6B TUBULIN)

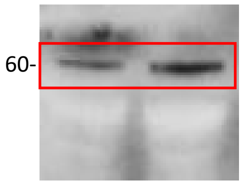

(FIg. 6C p-S6K1)

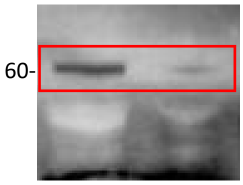

(FIg. 6C p-S6K1)

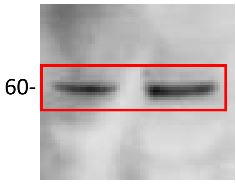

(FIg. 6C p-S6K1)

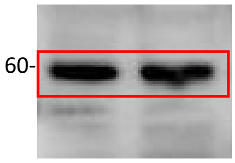

(FIg. 6C S6K1)

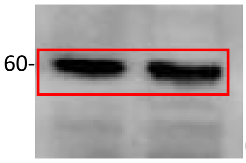

(FIg. 6C S6K1)

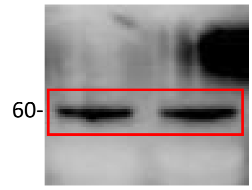

(FIg. 6C S6K1)

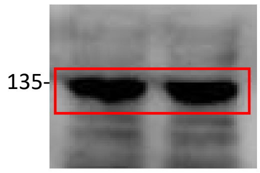

(FIg. 6C Raptor)

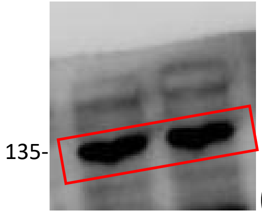

(FIg. 6C Raptor)

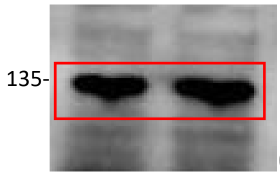

(FIg. 6C Raptor)

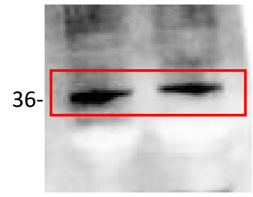

(FIg. 6C PP2Ac)

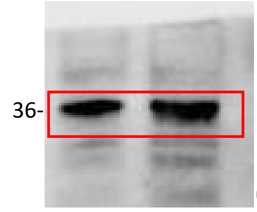

(FIg. 6C PP2Ac)

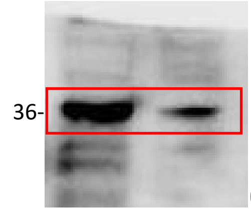

(FIg. 6C PP2Ac)

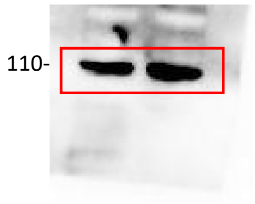

(FIg. 6C HIFa)

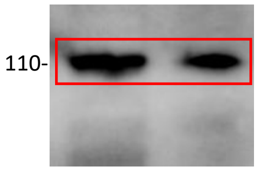

(FIg. 6C HIFa)

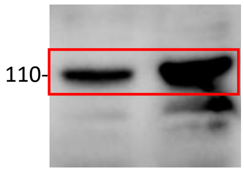

(FIg. 6C HIFa)

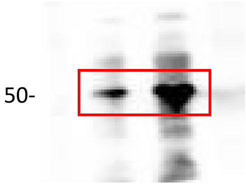

(FIg. 6C Myc)

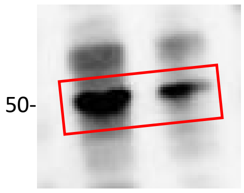

(FIg. 6C Myc)

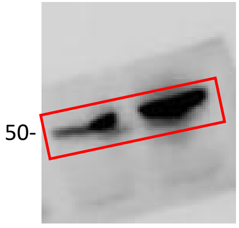

(FIg. 6C Myc)

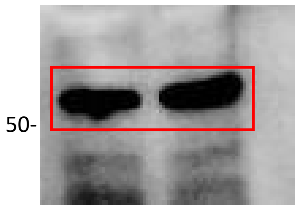

(FIg. 6C TUBULIN)

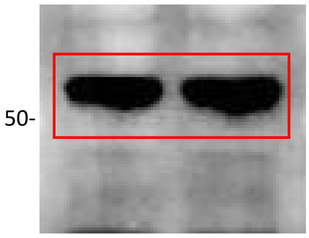

(FIg. 6C TUBULIN)

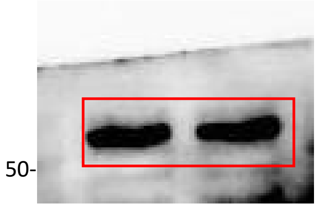

(FIg. 6C TUBULIN)

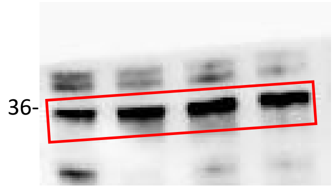

(FIg. 6D SNRPA)

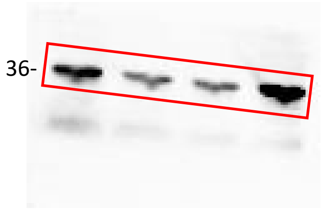

(FIg. 6D PP2Ac)

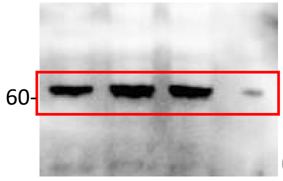

(FIg. 6D p-S6K1)

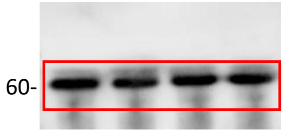

(FIg. 6D S6K1)

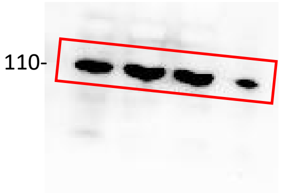

(FIg. 6D HIFa)

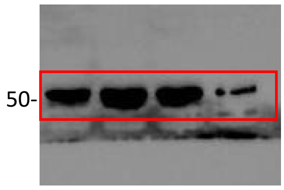

(FIg. 6D c-Myc)

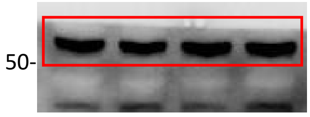

(FIg. 6D TUBULIN)

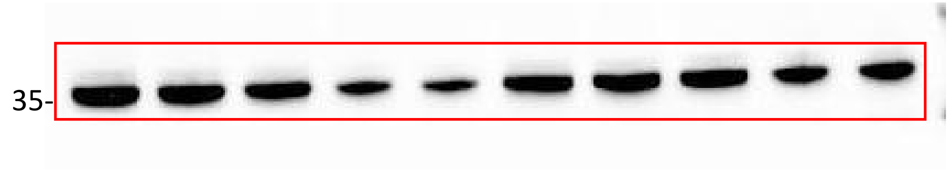

(FIg. 6E PP2Ac PANC-1 WT)

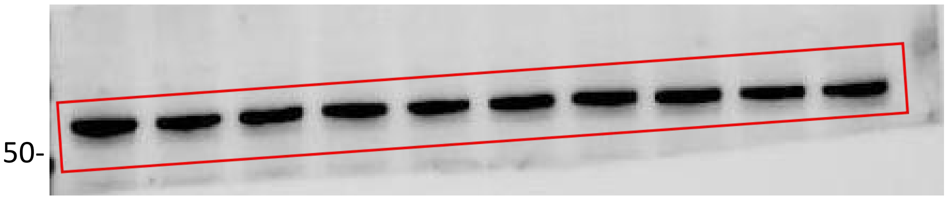

(FIg. 6E TUBULIN PANC-1 WT)

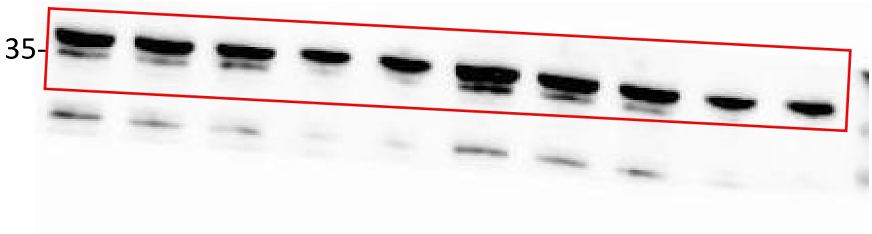

(FIg. 6E PP2Ac PANC-1 GR)

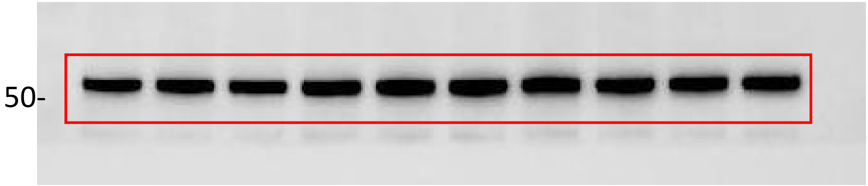

(FIg. 6E TUBULIN PANC-1 GR)

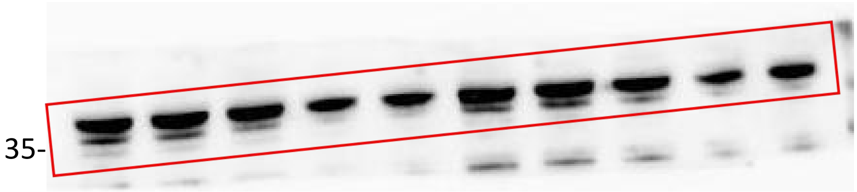

(FIg. 6F PP2Ac PANC-1 WT)

50-

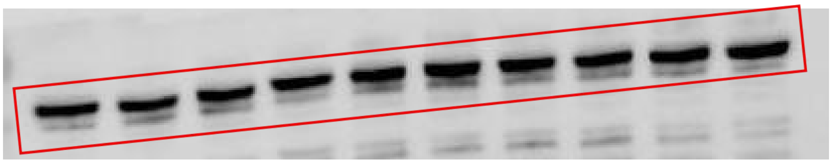

(FIg. 6F TUBULIN PANC-1 WT)

35-

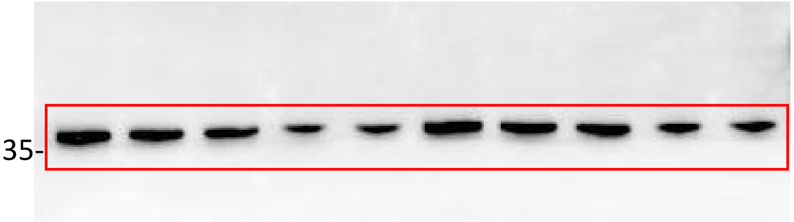

(FIg. 6F PP2Ac PANC-1 GR)

50-

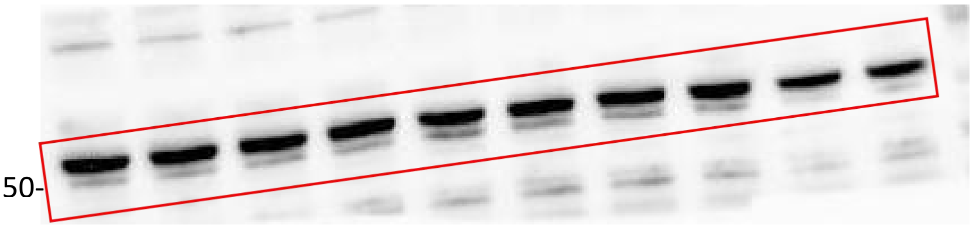

(FIg. 6F TUBULIN PANC-1 GR)

35-

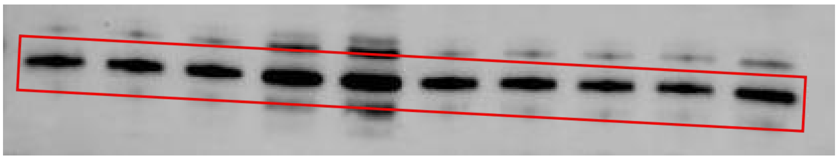

(FIg. 6G PP2Ac PANC-1 WT)

50-

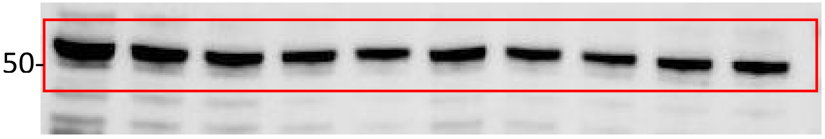

(FIg. 6G TUBULIN PANC-1 WT)

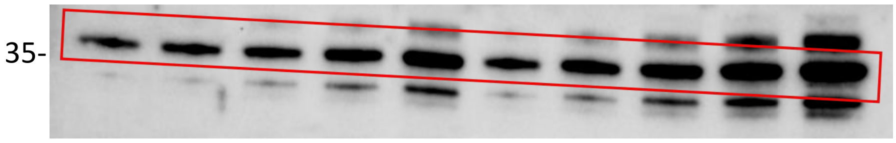

(FIg. 6G PP2Ac PANC-1 GR)

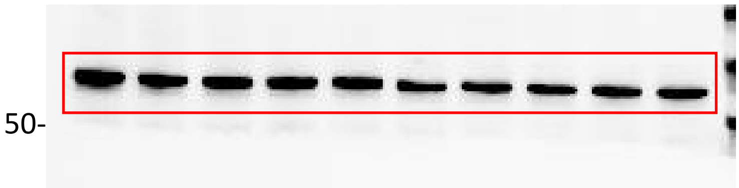

(FIg. 6G TUBULIN PANC-1 GR)

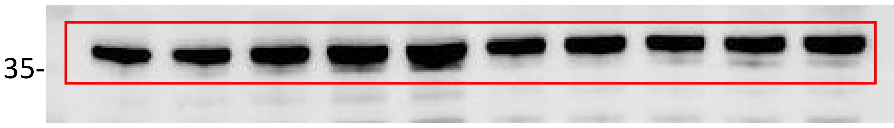

(FIg. 6H PP2Ac PANC-1 WT)

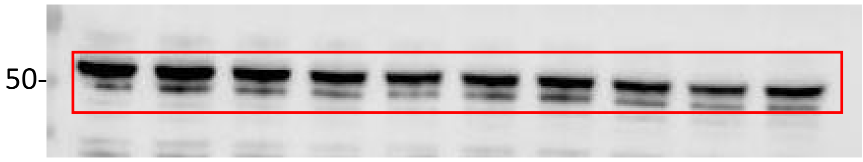

(FIg. 6H TUBULIN PANC-1 WT)

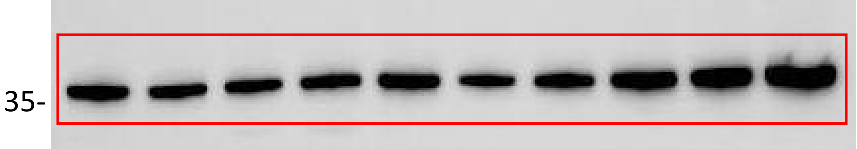

(FIg. 6H PP2Ac PANC-1 GR sh-NC)

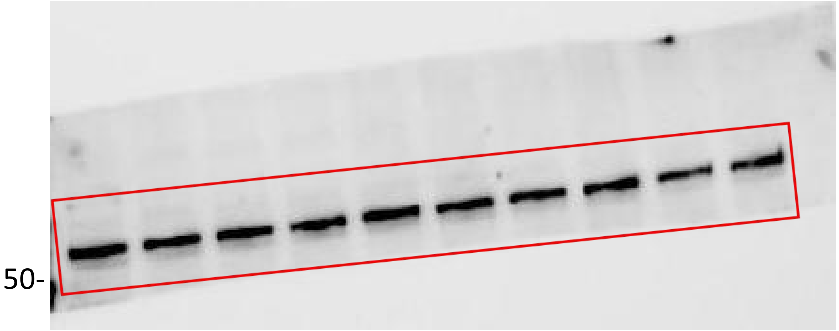

(FIg. 6H TUBULIN PANC-1 GR)

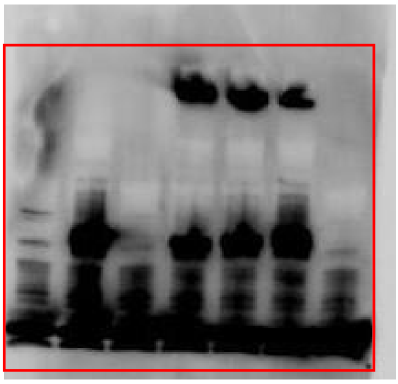

(FIg. 6I)

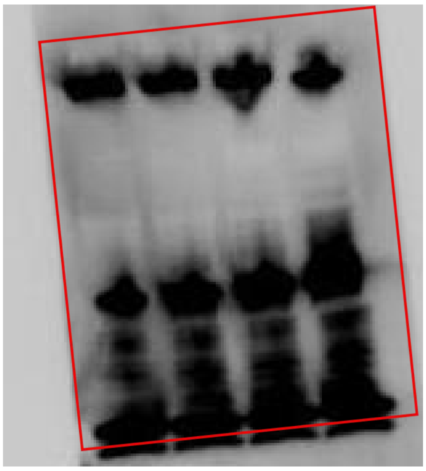

(FIg. 6J)

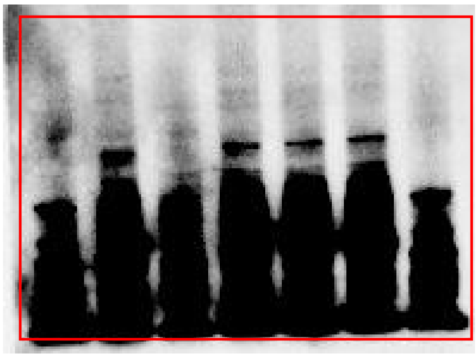
(FIg. 6K)

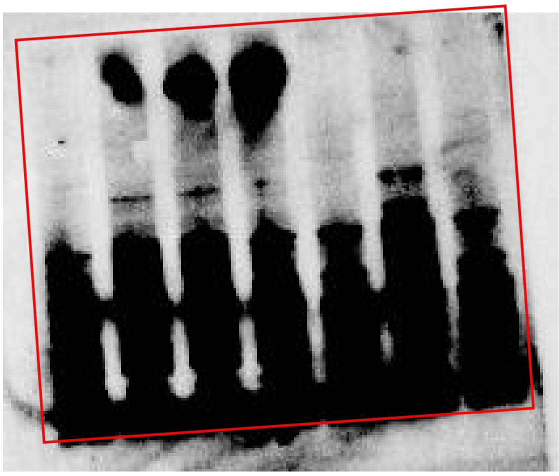
(FIg. 6L)

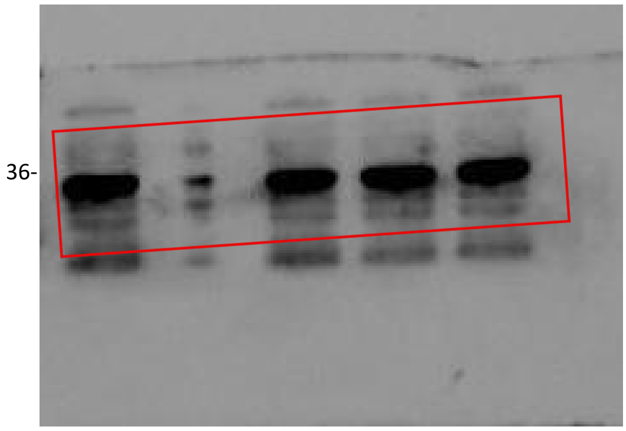

(FIg. 6M SNRPA)

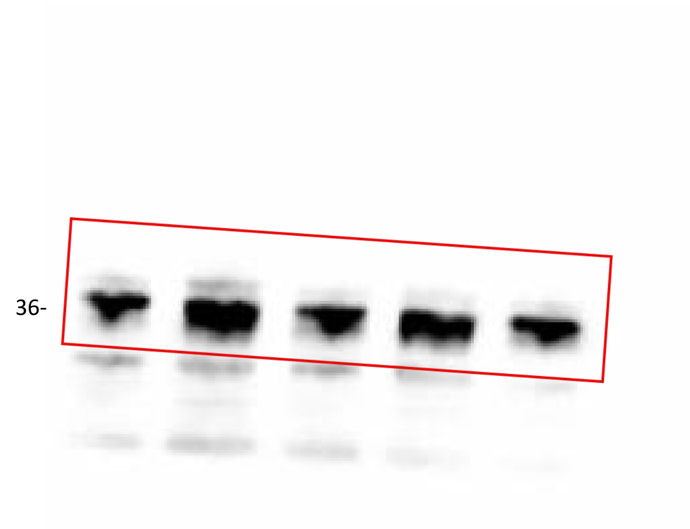

(Fig. 6M PP2Ac)

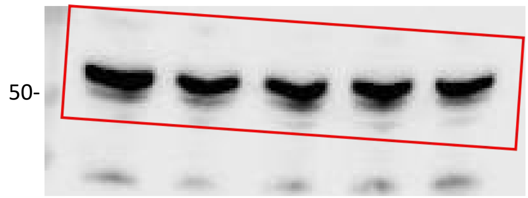

(Fig. 6M TUBULIN)

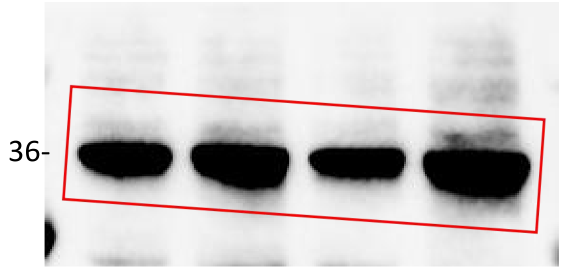

(Fig. S7F SNRPA)

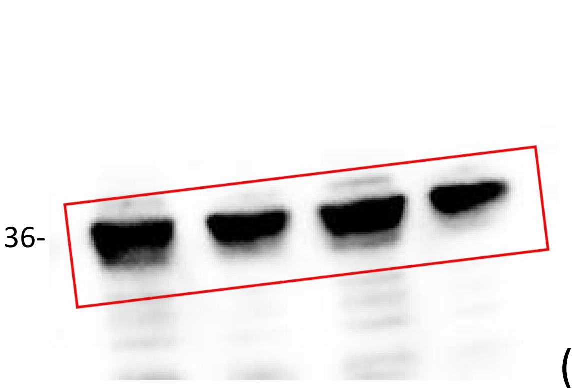

(Fig. S7F PP2Ac)

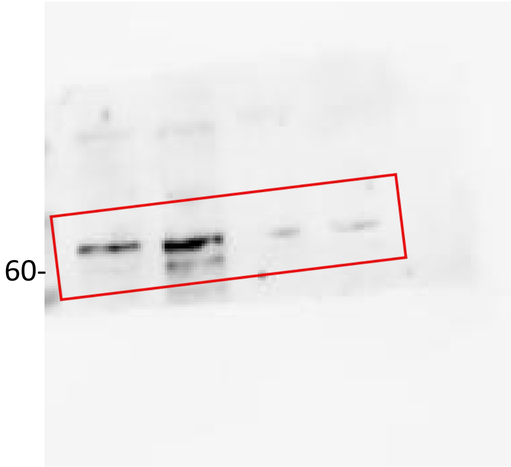

(Fig. S7F p-S6K1)

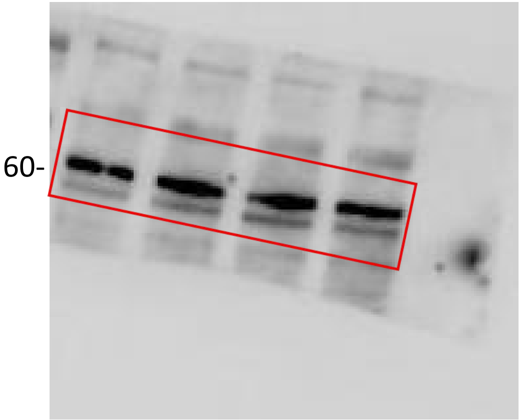

(Fig. S7F S6K1)

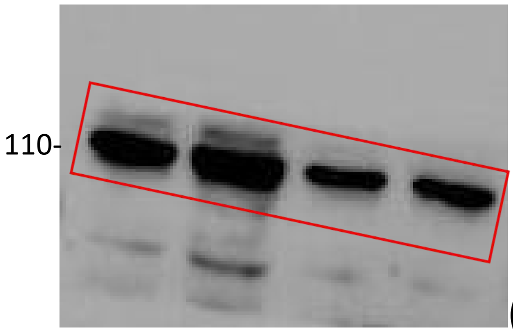

(Fig. S7F HIF1a)

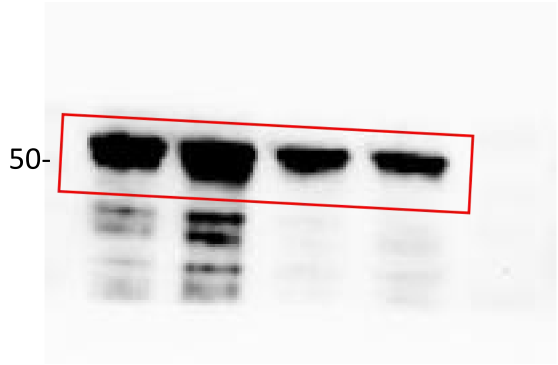

(Fig. S7F c-Myc)

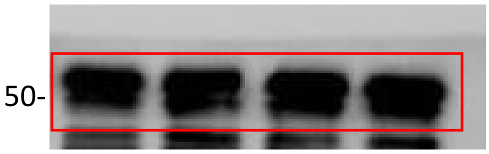

(Fig. S7F TUBULIN)

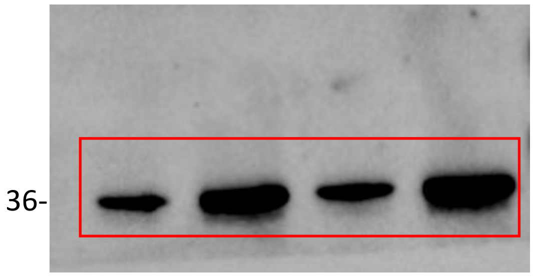

(Fig. S7G SNRPA)

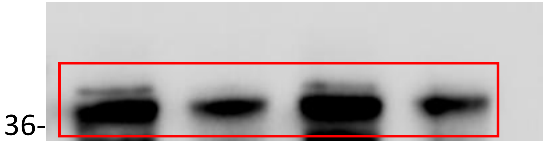

(Fig. S7G PP2Ac)

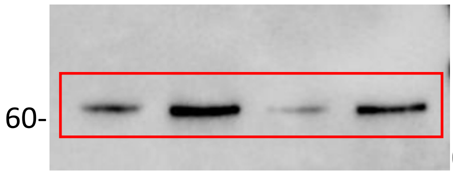

(Fig. S7G p-S6K1)

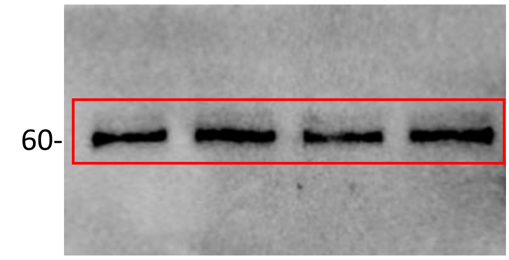

(Fig. S7G S6K1)

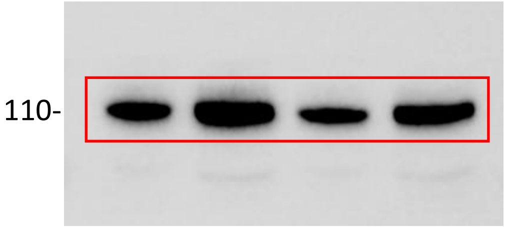

(Fig. S7G HIF1a)

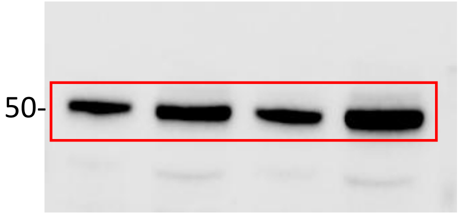

(Fig. S7G c-Myc)

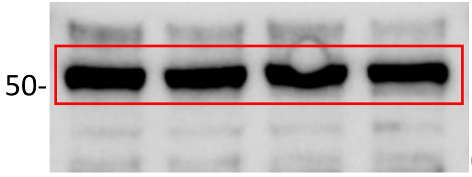

(Fig. S7G TUBULIN)

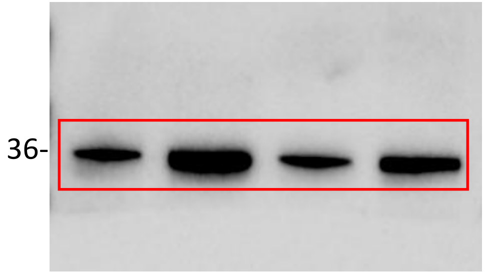

(Fig. S7H SNRPA)

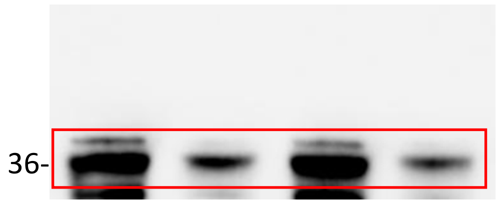

(Fig. S7H PP2Ac)

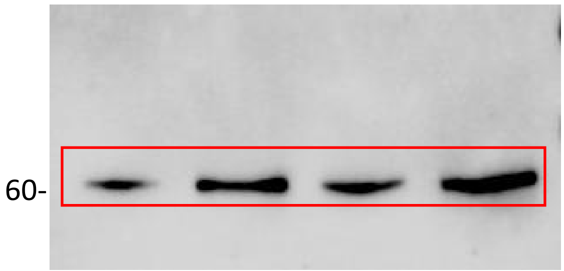

(Fig. S7H p-S6K1)

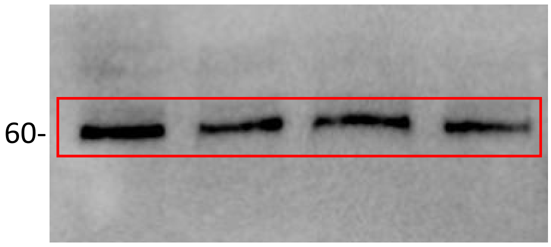

(Fig. S7H S6K1)

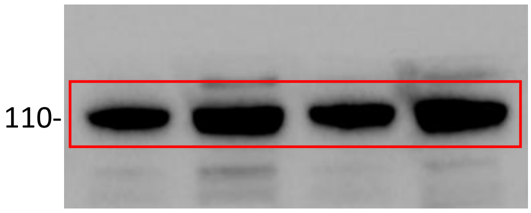

(Fig. S7H HIF1a)

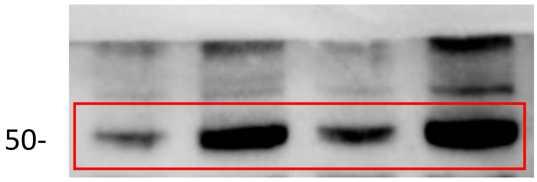

(Fig. S7H c-Myc)

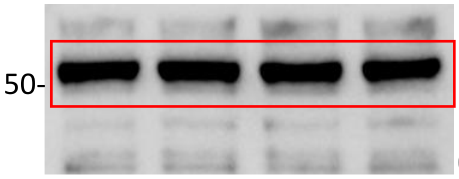

(Fig. S7H TUBULIN)
